# Dynamics of T cell subpopulations and plasma cytokines during the first year of antineoplastic therapy in patients with breast cancer: the BEGYN-1 study

**DOI:** 10.1186/s13058-025-01997-9

**Published:** 2025-04-01

**Authors:** Elisabeth Kaiser, Regine Weber, Melanie Hirschstein, Hala Mazid, Emilie Marie Suzanne Kapps, Muriel Charlotte Hans, Michelle Bous, Sybelle Goedicke-Fritz, Gudrun Wagenpfeil, Michael Zemlin, Erich-Franz Solomayer, Carolin Müller, Cosima Zemlin

**Affiliations:** 1 Department of General Pediatrics and Neonatology, Saarland University, Campus Homburg, 66421 Homburg/Saar, Germany; 2 Institute for Medical Biometry, Epidemiology and Medical Informatics (IMBEI), Saarland University, Campus Homburg, 66421 Homburg/Saar, Germany; 3 Department of Gynecology, Obstetrics & Reproductive Medicine, Saarland University, Campus Homburg, 66421 Homburg/Saar, Germany; 4 Outcomes Research Consortium, Department of Anesthesiology, Cleveland Clinic, Cleveland, OH USA; 5 Department of Gynecology and Obstetrics, Comprehensive Cancer Center Erlangen-EMN (CCC ER-EMN), Universitätsklinikum Erlangen, Friedrich-Alexander-Universität Erlangen-Nürnberg (FAU), Universitätsstraße 21-23, 91054 Erlangen, Germany

**Keywords:** Breast cancer, T cells, Cytokines, Chemotherapy, Endocrine therapy, Biomarkers

## Abstract

**Background:**

The role of T cell immunity during antineoplastic therapy is poorly understood. In the BEGYN-1 study, patients with breast cancer underwent quarterly assessments prior to and during antineoplastic therapy over a period of 12 months.

**Methods:**

We used flow cytometry and multiplex immunoassays to quantify 25 T cell subpopulations and seven T cell associated plasma cytokines in peripheral blood from 92 non-metastatic breast cancer patients, respectively. In addition, the association between T cell dynamics and the outcome of patients undergoing neoadjuvant chemotherapy was investigated.

**Results:**

In patients undergoing chemotherapy, a significant reduction in T helper (Th) cells, particularly naïve central and effector cells and thymus positive Th cells, was observed over time. Interestingly, Th1 immune response-associated cytokines (IL-12, TNF, IFN-γ) declined while Th2 cells and cytotoxic T cells increased over time.

**Conclusions:**

We conclude that in breast cancer patients, chemotherapy is associated with a transition from a Th1 immune response towards Th2 and an increase in cytotoxic T cells, whereas in patients without chemotherapy, these alterations were less pronounced. Future studies should clarify whether patterns of T cell subsets or plasma cytokines can be used as biomarkers to monitor or even improve therapeutic interventions.

**Graphical abstract:**

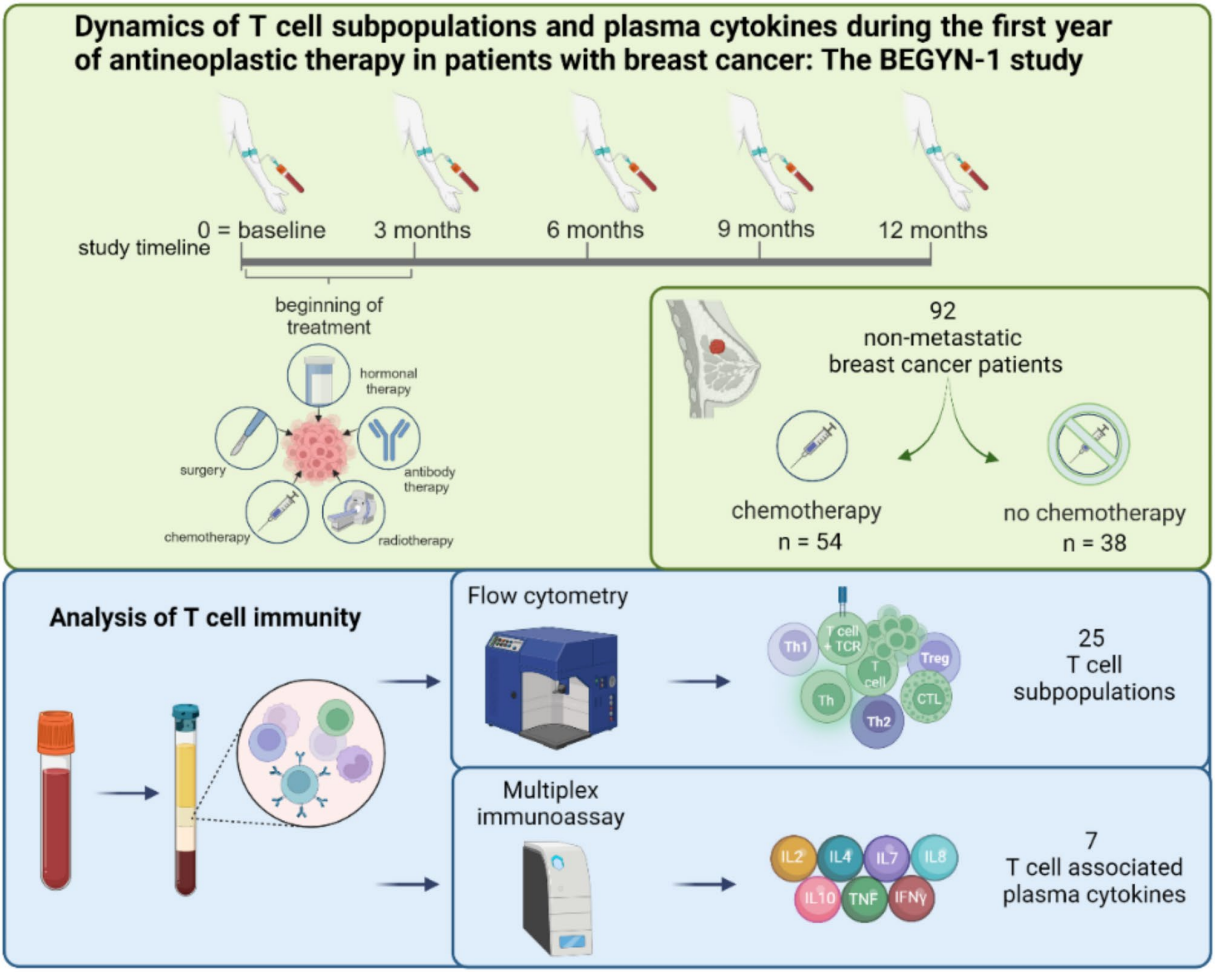

**Supplementary Information:**

The online version contains supplementary material available at 10.1186/s13058-025-01997-9.

## Introduction

Breast cancer is the most common malignancy in women, accounting for almost 25% of new cancers in women and over 680,000 deaths per year worldwide [1, 2].

Oncological therapy for breast cancer, encompassing chemotherapy, endocrine therapy, surgery, and radiation therapy, aims to eliminate cancer cells and control and stop their further spread and metastasis. These therapeutic interventions also affect the immune system. The immune status of cancer patients is constantly changing during the course of the disease and treatment, making the analysis of immunological components as potential biomarkers a valuable diagnostic and prognostic tool [3–8].

In the development and growth of breast cancer, immune cells can play a crucial, dual role: on the one hand, they can create a tumor-friendly inflammatory environment and, on the other hand, they can cause tumor rejection. Understanding the interaction between tumor cells and cells of the immune system is of great importance as the effect of an immune response largely depends on the nature of the stimulated immune response. T cells represent key cells in the tumor microenvironment and are classified into different subtypes based on their transcription factors, markers and functions [9, 10].

Th1 cells are often considered to be critical components of the anti-tumor immune response in tumor immunology, as they possess the ability to produce IFN-γ, activate macrophages and enhance CD8 + T killer cells. Th2 cells, on the other hand, have been linked to the promotion of cancer development and metastasis [11]. CTLs are traditionally considered to be a key component of effective anti-tumor immunity, and their high infiltrtion into the tumor tissue is associated with better prognosis and survival [12, 13]. Memory T cells play a crucial role in anti-tumor immunity as they can induce a rapid and sustained immune response to tumors [14].

The role of individual T-cell subsets in the breast tumor microenvironment and their association with breast cancer outcome is the subject of many current studies, with no clear consensus to date [13, 15–20]. In addition, the role of T cells in breast cancer immunity depends on a variety of extrinsic factors, including tumor type or subtype, disease stage, immunogenicity of the tumor, localization of cells in tumor tissue, and interaction with other cells or cytokines [9].

Given the complexity of both the immune system and the tumor process, extensive research is required to understand their interactions. The analysis of T-cell subpopulations and associated cytokines in peripheral blood is often performed to determine the immune status and correlation of various parameters such as disease, age or lifestyle [21–23]. For instance, evidence suggests that certain subpopulations of cells from peripheral blood might contribute to the pool of tumor infiltrating regulatory T cells and could serve as potential targets for immunotherapeutic interventions in breast cancer [24–26]. However, current studies have only examined selective connections between breast cancer, antineoplastic therapies, and specific components of the immune system, often in small cohorts or over brief periods [25, 27–31]. Consequently, the potential interconnections among these factors remain poorly understood.

The need for prospective studies investigating factors influencing the immune system in cancer patients is substantial, particularly to develop potential individualized rehabilitation approaches [32–35].

The BEGYN-1 study adopts a holistic approach to investigate the impact of the disease and anti-cancer therapies on the immune system. Additionally, it examines the effects of supportive therapies that may positively influence the disease course and the immune system, including physical activity, nutrition, and body composition, alongside other factors. We previously published results of the BEGYN-1 study focusing on the physical activity during the first year after breast cancer diagnosis and during different treatments (e.g., chemotherapy, radiotherapy) [36]. We showed that patients were able to maintain or even improve their fitness during oncological treatment. Moreover, data on vitamins and supplements (vitamin D and selenium) was monitored within the BEGYN-1 study and we saw a high rate of vitamin D deficiency, whereas selenium deficiency was rare [37–39].

In the present study, we employed validated methods for the first time to closely monitor T-cell immune status at the level of multiple T-cell subpopulations and their associated cytokines in peripheral blood during the first year of antineoplastic therapy and immediately after diagnosis in a large cohort of non-metastatic breast cancer patients. This study includes baseline data collection before any therapeutic intervention and subsequent data collection at quarterly intervals [35].

Here we present data from the BEGYN-1 cohort, focusing on the interactions between various oncological therapies and immunological responses in peripheral blood. We aim to examine both the immediate effects and the potential long-term consequences on the immune system. Although the importance of a functioning immune system is undisputed, there is a lack of comprehensive knowledge about the nature of these changes. The inclusion of the immune status in diagnostics and therapy decisions in breast cancer patients still plays a subordinate role.

## Materials and methods

### Study design and data collection

For the BEGYN-1 study, 110 patients were recruited at the Saarland University Medical Center between September 2019 and January 2021 after informed consent according to the previously published study protocol [35]. The inclusion criteria of the BEGYN-1 study were age ≥ 18 years, ability to fill questionnaires in German language and to use a smartphone and fitness tracker, as well as a physical condition permitting ergospirometry on a treadmill. Exclusion criteria were pregnancy, metastasized disease or secondary carcinomas, and patients with a life expectancy of less than one year. Patients were encouraged to exercise but it was not mandatory or an inclusion criterion to participate in the study. The BEGYN-1 study encompassed different outcomes, including the determination of T cell subpopulations and plasma cytokines in newly diagnosed breast cancer patients which is presented in the present manuscript. Accordingly, the baseline assessment was performed after diagnosis of non-metastasized breast cancer and before initiation of any therapeutic intervention. The same applied for the blood samples. Baseline (0 months) blood samples were drawn prior to any therapeutic intervention. Follow-up visits were performed quarterly until 12 months after diagnosis. Each study visit included clinical assessments, medical history reviews, and blood sampling [35].

**Fig. 1:**
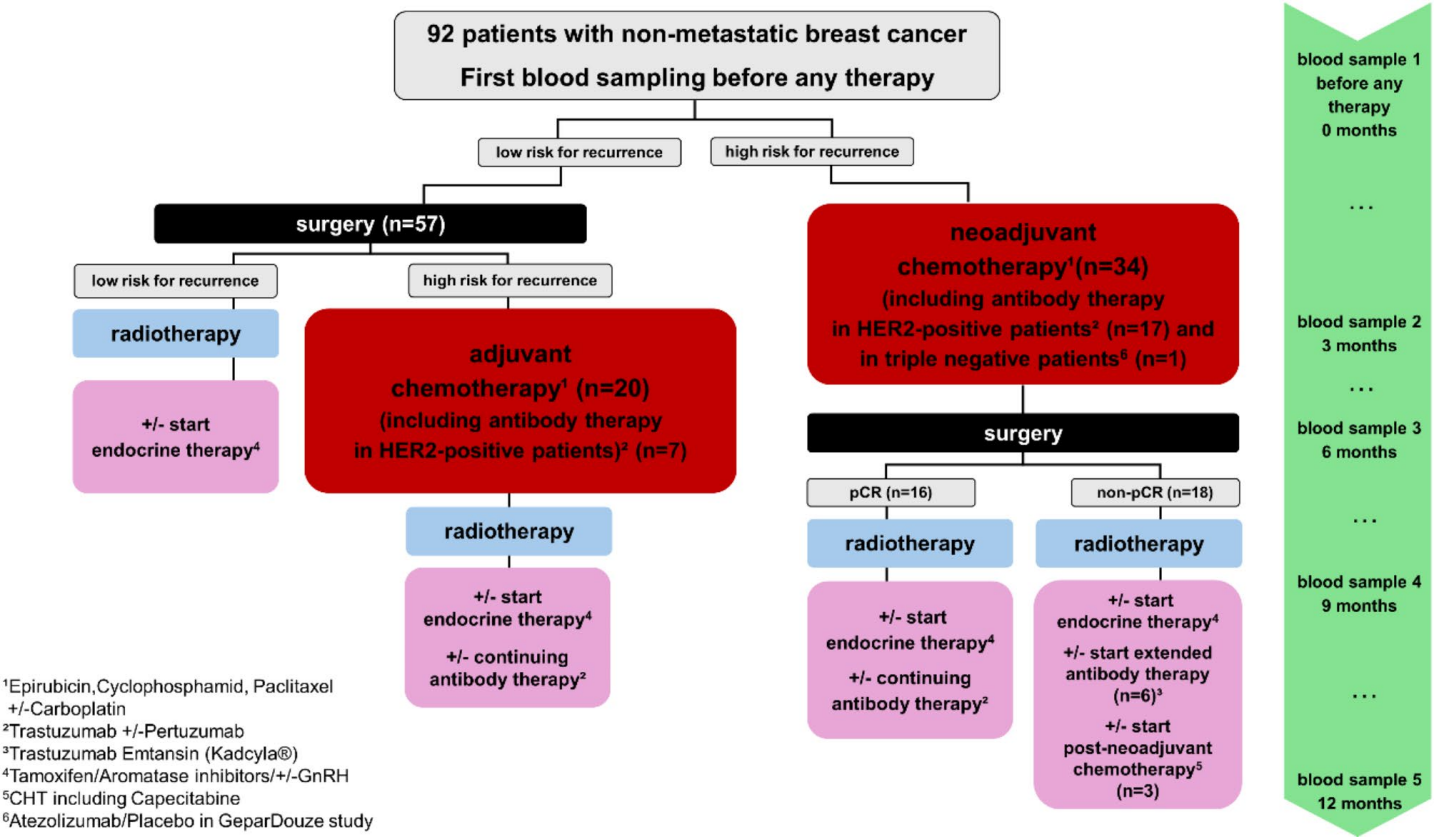
Treatment pattern of the patients. The individual therapy interventions (red: adjuvant and neoadjuvant chemotherapy; blue: radiotherapy; black: surgery; pink: maintenance therapy like endocrine therapy, antibody therapy or post- neoadjuvant chemotherapy). Green: blood samples for the BEGYN-1 study (first blood sample/baseline measurement at 0 weeks, green: blood samples after 3, 6, 9 and 12 months) over time. The basis for this is provided by the German S3 guideline. The individual treatment of the patients is shown in Figures S3 and S4

Patients were divided in two groups: (1) patients who received chemotherapy (CHT) and (2) patients who did not receive any chemotherapy (NCHT). All patients received standardized chemotherapy according to international and national guidelines [40, 41]. Chemotherapy regimens included anthracyclines and taxanes. According to the guidelines [40, 41], triple negative carcinomas received additional carboplatin and/or immunotherapy and HER2-positive patients received anti-HER2 agents. An overview of the treatment pattern is shown in Fig. 1, individual patient treatment patterns are shown in Supplementary Figure S3 and S4.

### Methods

#### Peripheral blood samples

Plasma and peripheral blood mononuclear cells (PBMCs) were obtained from EDTA blood samples by routine procedures. Briefly, plasma was obtained by centrifugation at 400 g for 30 min prior to cryopreservation at -80 °C. PBMCs were isolated by density gradient centrifugation using the Lymphocyte Separation Medium 1.077, FicoLite-H (#GTF1511KYA, Linaris biological products, Dossenheim, Germany). Resulting interphase, the PBMC fraction were washed twice using Dulbecco’s phosphate-buffered saline (D-PBS, #D8537, Sigma Aldrich, Steinheim, Germany) and were centrifuged at 400 g for 10 min. Subsequently, cells were resuspended in fetal bovine serum (FBS, #11573397, Thermo Fisher Scientific, Waltham, MA, USA) + 10% dimethylsufoxid (DSMO, #D8418, Sigma Aldrich, Steinheim, Germany). The samples were prepared for cryopreservation at − 80 °C using a CellCamper^®^.

#### Flow cytometry

Cryopreserved cells were thawed on the day of the experiment, washed twice with D-PBS and centrifuged at 400 g for 10 min. The number and viability of cells were determined using acridine orange and propidium iodide (AO/PI, #F23001, Logos Biosystems, Dongan-gu Anyang-si, Gyeonggi-do, South Korea) and LUNA-FL™ Automated Fluorescence Cell Counter (Logos Biosystems, Dongan-gu Anyang-si, Gyeonggi-do, South Korea). Staining of dead cells was performed with BD Horizon Fixable Viability Stain 780 (#565388, BD Biosciences, Heidelberg, Germany), followed by washing with BD CellWASH™ (#349524, BD Biosciences, Heidelberg, Germany) and centrifugation (10 min, 500 g). Cell pellets were resuspended in a panel-specific antibody mix (Supplementary Table 1) containing BD Horizon™ Brilliant Stain Buffer (#563794, BD Biosciences, Heidelberg, Germany) and BD Pharmingen™ Stain buffer BSA (#554657, BD Biosciences, Heidelberg, Germany). After 30 min of light protected incubation, the remaining erythrocytes were lysed with BD FACS™ Lysing Solution (#349202, BD Biosciences, Heidelberg, Germany) according to the manufacturer’s protocol. Thereafter, the cell suspension was diluted with BD CellWASH™ and centrifuged at 500 g for 10 min. Cell pellets resuspended in D-PBS were analyzed using a 3-laser 12-color FACS Celesta flow cytometer (Becton, Dickinson and Company, Heidelberg, Germany). All cell samples from a patient were analyzed on the same day using the same antibody master mix that was prepared on the day of measurement. Whenever possible, the same batch of reagents was used for all study samples. The cell samples were thawed for the first time for flow cytometric analysis. For the quality control, the following approaches were used: The Cytometer Setup and Tracking Module (CS&T, #655051, BD Biosciences, Heidelberg, Germany) was used to check and maintain the flow cytometer performance, stability, fluorescence calibrations and reproducibility of the data on a daily basis. To check the reproducibility of the data over time, Sphero™ Rainbow Calibration Particles (8 Peaks, #559123, BD Biosciences, Heidelberg, Germany) were used and the application settings were used for each acquisition. Compensation settings were calculated using BD™ CompBeads (#552843, BD Biosciences, Heidelberg, Germany). Based on the comparison of the immunostained sample with an unstained sample, a sample subjected to all procedures except antibody staining, and the isotype control to determine the non-specific binding of the antibodies, the positive staining and gating strategy was determined (Supplementary Figs. 1 & 2). Cell aggregates were removed from the analysis (FSC-A/FSC-H) and dead cells were excluded from the analysis by staining with BD Horizon Fixable Viability Stain 780. The classification of lymphocytes according to morphological parameters (FSC-A/SSC-A) was confirmed at the end. Data were acquired with FACSDiva (BD Biosciences, Heidelberg, Germany) and analyzed using FlowJo v10 (BD Biosciences).

#### Multiplex immunoassay

Plasma samples were thawed at room temperature, vortexed and centrifuged at 1000 g for 10 min. A MILLIPLEX^®^ Human High Sensitivity T Cell Mag Panel (#HSTCMAG-28SK, Merck KGaA, Darmstadt, Germany) multiplex immunoassay with customizable selection of analytes was used for simultaneous determination of multiple cytokines. The following analytes were included in the panel (the abbreviations used consecutively, and the catalog numbers of the antibody-immobilized magnetic microspheres selected for the immunoassay are in brackets): Tumor necrosis factor (TNF, #HCYTNFA-MAG), interferon-γ (IFN–γ, #HCYIFNG-MAG), interleukin-4 (IL-4. #HIL4-MAG), interleukin-7 (IL-7, #HIL7-MAG), interleukin-8 (IL-8, #HCYIL8-MAG), interleukin-10 (IL-10, #HCYIL10-MAG), interleukin-12 subunit p70 (IL-12, #HIL12P70-MAG). A total of 18 kits of the assay with the same batch number of included consumables were used. Samples from different time points of a patient were always analyzed with one batch, while the distribution of the patient samples among the 18 HSTCMAG-28SK assays was random. Samples, standards (in seven defined concentration levels by serial dilution), and quality controls (in two defined concentrations) were transferred in technical triplicates to a 96-well microtiter plate. Antibody-immobilized magnetic microspheres were sonicated and vortexed, pooled, completed with included microsphere diluent. The microsphere mix was pipetted into all wells. The 96-well microtiter plate was incubated overnight at + 4 °C. After repeated washing steps, a biotinylated detection antibody cocktail was added and incubated for an hour, followed by streptavidin-phycoerythrin (PE) incubation for 30 min to label multiplexes, both steps at room temperature and with shaking. After final washing steps, MAGPIX™ Drive Fluid Plus (Luminex Corp., Texas, United States) was added prior to data acquisition on MAGPIX^®^ instruments with xPONENT^®^ Software (Luminex Corp., Texas, United States). When cytokines of interest were present in the sample, they were captured by the cytokine-specific antibodies coupled to color-coded magnetic microspheres and formed a sandwich with the biotinylated detection antibodies. The addition of PE-conjugated streptavidin enabled subsequent detection and quantification: A magnet in the MAGPIX^®^ instrument captures the magnetic microspheres in a monolayer when the sample is presented, while two different light-emitting diodes (LEDs) illuminate the microspheres. One LED (621 nm) is used to identify the cytokine to be detected by the color-code and the second LED (511 nm) is used to determine the magnitude of the signal derived from PE. MAGPIX^®^ instrument was calibrated and validated weekly as it is described in the manufacturer’s instructions using the MAGPIX^®^ calibration kit (MPX-CAL-K25, Luminex Corp., Texas, United States) and MAGPIX^®^ performance verification kit (#MPX-PVER-K25, Luminex Corp., Texas, United States). Sample values for each cytokine were determined following standard curve calculation by using 4 and 5 parameter logistics weighted curve fitting algorithms with Belysa^®^ version 1.2 (Millipore by Merck, Massachusetts, United States). Sample values below limits of detection were set to half of the minimal detectable concentration specified by the manufacturer of the immunoassay.

Quality controls were within the manufacturer’s specifications for all analytes and showed a maximum inter-assay coefficient of variation of 12.3% (mean CV quality control 1: 10.1%; mean CV quality control 2: 6.4%.).

#### Statistical analysis

Statistical analysis was performed using Prism 10.1.2 for Windows (GraphPad Software LLC, Boston, MA, USA) and SPSS (IBM Corp. Released 2023. IBM SPSS Statistics for Windows, Version 29.0.2.0 Armonk, NY: IBM Corp.). Quantitative data derived from protein multiplex immunoassay (all patients) and flow cytometric analyses of cell populations were subjected to the Shapiro Wilk test for normal distribution. Therapy group differences (CHT vs. NCHT and pCR vs. non-pCR) of cell population and cytokine data were assessed using two-tailed Mann Whitney U test, differences over time within one group were assessed using two-tailed Wilcoxon matched-paired signed rank test without p value adjustment for multiple comparisons. Therapy group differences (CHT vs. NCHT and pCR vs. non-pCR) of patient’s characteristics were assessed using two-tailed Fisher’s exact test or Fisher-Freeman-Halton’s exact test. Confidence level for statistical significance was set at 95%. Number of asterisks indicate p values as follows: * *p* < 0.05, ** *p* < 0.01, *** *p* < 0.005, **** *p* < 0.001.

## Results

### Patient characteristics

During the recruitment period, 110 patients were recruited to the BEGYN-1 study. 18 patients were excluded from further analyes because they dropt out of the study due to the following reasons: distress (*n* = 9), technical problems with the fitness tracker (*n* = 3), stress to fill out the diary and/or questionnaires (*n* = 2), further treatment in another clinic (*n* = 2), secondary metastasis (*n* = 1), secondary paralysis (*n* = 1). Characteristics of the 92 patients that completed the study with complete data sets are given in Table 1. Treatment groups are listed in Table 2.

The patients were divided into a chemotherapy group that had received chemotherapy at any time (CHT group) and a non-chemotherapy group (NCHT group). Longitudinal dynamics were studied by comparing the time points of assessment at 3, 6, 9 and 12 months compared to the baseline visit (0 months). We focused on the comparisons of time points 3 and 12 with each other and against the baseline, because the aim of our longitudinal analysis is to characterize to what extent and how long antineoplastic therapy alters the immune status and whether the immune status approaches baseline during the first year after diagnosis of breast cancer. Thus, the 3-month follow-up serves as a reference for the dynamics beyond the initial steps of therapy. At each time point (0, 3, 6, 9, 12 months), *n* = 72 to 82 samples passed all quality control standards (blood volume, constant number of thawing cycles, concentration of dead cells) for flow cytometry and cytokine assays and were included in the statistical analyses. Sample sizes per time point are given in Table S2 and Table S5.

As different systemic therapies could have an impact on immune cell populations, we also performed a subgroup analysis excluding patients receiving anti-HER2 agents, Atezolizumab, Cepecitabine or Abemaciclib (Figure S5-Figure S9).

**Table 1:** Patient characteristics: age, tumor entity, grading, tumor stage (TNM-classification) and type of therapy

		All patients		CHT		NCHT		
Age (median, min/max)		55.0 years (26 / 78 years)		50.5 years (27 / 75 years)		59.0 years (26 / 78 years)		p-value
		n	percentage	n	percentage	n	percentage	
Total		92	100.0%	54	58.7%	38	41.3%	-
**Tumor** **entity**	NST	77	83.7%	44	81.5%	33	86.8%	0.383^#^
	invasiv lobular	9	9.8%	7	13.0%	2	5.3%	
	others	6	6.5%	3	5.5%	3	7.9%	
**cT**	cT0*	3	3.3%	2	3.7%	1	2.6%	< 0.0001^#^
	cT1	64	69.5%	29	53.6%	35	92.1%	
	cT2	21	22.8%	19	35.2%	2	5.3%	
	cT3	1	1.1%	1	1.9%	0	0.0%	
	cT4	3	3.3%	3	5.6%	0	0.0%	
**cN**	cN0	74	80.4%	38	70.4%	36	94.7%	0.003^‡^
	cN+	18	19.6%	16	29.6%	2	5.3%	
**M**	M0	92	100.0%	54	100.0%	38	100.0%	-
Grading**	G1	10	10.9%	0	0.0%	10	26.3%	< 0.0001^#^
	G2	43	46.7%	20	37.0%	23	60.5%	
	G3	38	41.3%	34	63.0%	4	10.5%	
**Receptor**	Luminal A	40	43.4%	8	14.8%	32	84.2%	< 0.0001^#^
	Luminal B	18	19.6%	15	27.8%	3	7.9%	
	HER2 positive	26	28.3%	25	46.3%	1	2.6%	
	Triple negative	8	8.7%	6	11.1%	2	5.3%	

Patients were divided in two groups: (1) patients who received chemotherapy (CHT) and (2) patients who did not receive any chemotherapy (NCHT). “NST“= No special type. *Axillary local recurrence without evidence of tumor in the breast **Grading not available in one NCHT patient. ^#^Fisher-Freeman-Halton’s exact test. ^‡^Fisher’s exact test

**Table 2:** Treatments according to time quartiles

		All patients		CHT		NCHT	
	Time of assessment (months)	n	percentage	n	percentage	n	percentage
surgery	3	56	60.9%	19	35.2%	37	97.4%
	6	15	16.3%	15	27.8%	0	0.0%
	9	21	22.8%	20	37.0%	1	2.6%
	12	0	0.0%	0	0.0%	0	0.0%
radiotherapy	3	24	26.1%	0	0.0%	24	63.2%
	6	17	18.5%	2	3.7%	15	39.5%
	9	44	47.8%	42	77.8%	2	5.3%
	12	15	16.3%	15	27.8%	0	0.0%
chemotherapy	3	54	58.7%	54	100.0%	0	0.0%
	6	53	57.6%	53	98.1%	0	0.0%
	9	19	20.7%	19	35.2%	0	0.0%
	12	0	0.0%	0	0.0%	0	0.0%
endocrine therapy^1^	3	41	44.6%	8	14.8%	33	86.8%
	6	49	53.3%	15	27.8%	34	89.5%
	9	67	72.8%	33	61.1%	34	89.5%
	12	71	77.2%	37	68.5%	34	89.5%
antibody therapy ^2^	3	17	18.5%	17	30.4%	0	0.0%
	6	25	27.2%	25	44.6%	0	0.0%
	9	25	27.2%	25	44.6%	0	0.0%
	12	25	27.2%	25	44.6%	0	0.0%
	total	92	100.0%	54	100.0%	38	100.0%

Table 2 indicates when patients received a given category of therapy during the BEGYN-1 study. Patients were divided in two groups: (1) patients who received chemotherapy (CHT) and (2) patients who did not receive any chemotherapy (NCHT). Individual data is illustrated in Supplementary Figure S3 (CHT) and Supplementary Figure S4 (NCHT). In summary, *n* = 54 patients underwent chemotherapy, all (*n* = 92) patients had surgery, *n* = 81 patients received radiotherapy, and *n* = 72 patients received endocrine therapy (Tamoxifen/Aromatase inhibitor +/- GnRH) [1]. *n* = 21 patients received chemotherapy with anthracyclines and taxanes, *n* = 9 patients received additionally platinum, *n* = 25 patients received chemotherapy in combination with antibody therapy [2]: *n* = 1 patient received chemotherapy in combination with Atezolizumab (checkpoint inhibitor)/placebo according to the GeparDouze study, *n* = 24 patients received chemotherapy in combination with anti-HER2 agents (Trastuzumab +/**-** Pertuzumab) including a maintanence of the HER2 therapy with *n* = 6 patients who received trastuzumab emtansin after surgery because of a non-pCR. Values are given as n = number of patients and percentage (%).

### Changes in T cell subpopulations over time

**Fig. 2:**
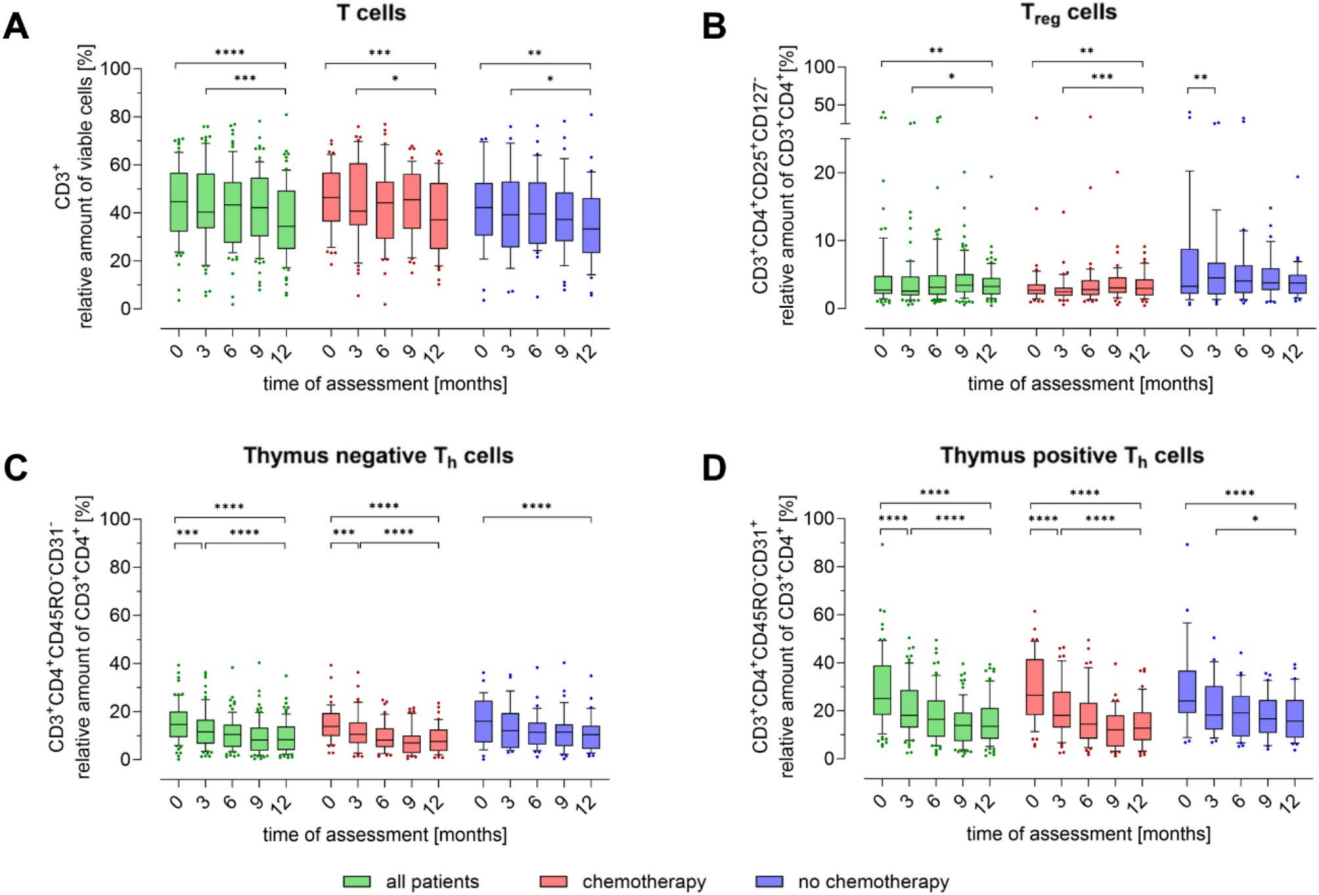
Peripheral blood T cell populations in breast cancer patients over the course of one year. The proportion of (**A**) T cells (CD3+), (**B**) regulatory T cells (Treg CD3 + CD4 + CD25 + CD127-), (**C**) thymus negative T cells (CD3 + CD4 + CD45RO-CD31-), (**D**) thymus positive T cells (CD3 + CD4 + CD45RO-CD31+) in relation to the parent cell population was determined quarterly by flow cytometry. The first measurement (baseline, 0 months) was performed after diagnosis and before initiation of therapy. Boxes extend from the 10th to the 90th percentiles. Points below and above the whiskers are drawn as individual data points. * *p* < 0.05, ** *p* < 0.01, *** *p* < 0.005, **** *p* < 0.001, therapy group differences were assessed using two-tailed Mann Whitney U test, differences over time within one group were assessed using two-tailed Wilcoxon matched-paired signed rank test

Throughout the first year following diagnosis, the number of T-cells (CD3+) declined in all patients (Fig. 2A). This trend was more pronounced in the CHT group (*p* < 0.005) than in the NCHT group (*p* < 0.01). Interestingly, the T cell subpopulations were not equally affected by the decline in overall T cell numbers.

The proportion of regulatory T cells (Treg) increased significantly over 12 months, primarily driven by changes in the CHT group, particularly when comparing the time points 3 to 12 months within the CHT group (*p* < 0.005, Fig. 2B). The NCHT group showed a significant increase in Treg after 3 months (*p* < 0.01), with a trend towards normalization by 12 months. Thymus negative Th cells (CD3 + CD4 + CD45RO-CD31-, Fig. 2C) and thymus positive (CD3 + CD4 + CD45RO-CD31+, Fig. 2D) were significantly reduced in both treatment groups after 12 months. Notably, this significant decrease in the CHT group was observed as early as 3 months in both thymus negative (*p* < 0.005) and thymus positive (*p* < 0.001) Th cells. Within the both treatment groups, CHT and NCHT, a significant reduction in both cell populations, thymus negative and thymus positive Th cells, was also observed when comparing baseline and 12-month measurements (*p* < 0.001 respectively).

**Fig. 3:**
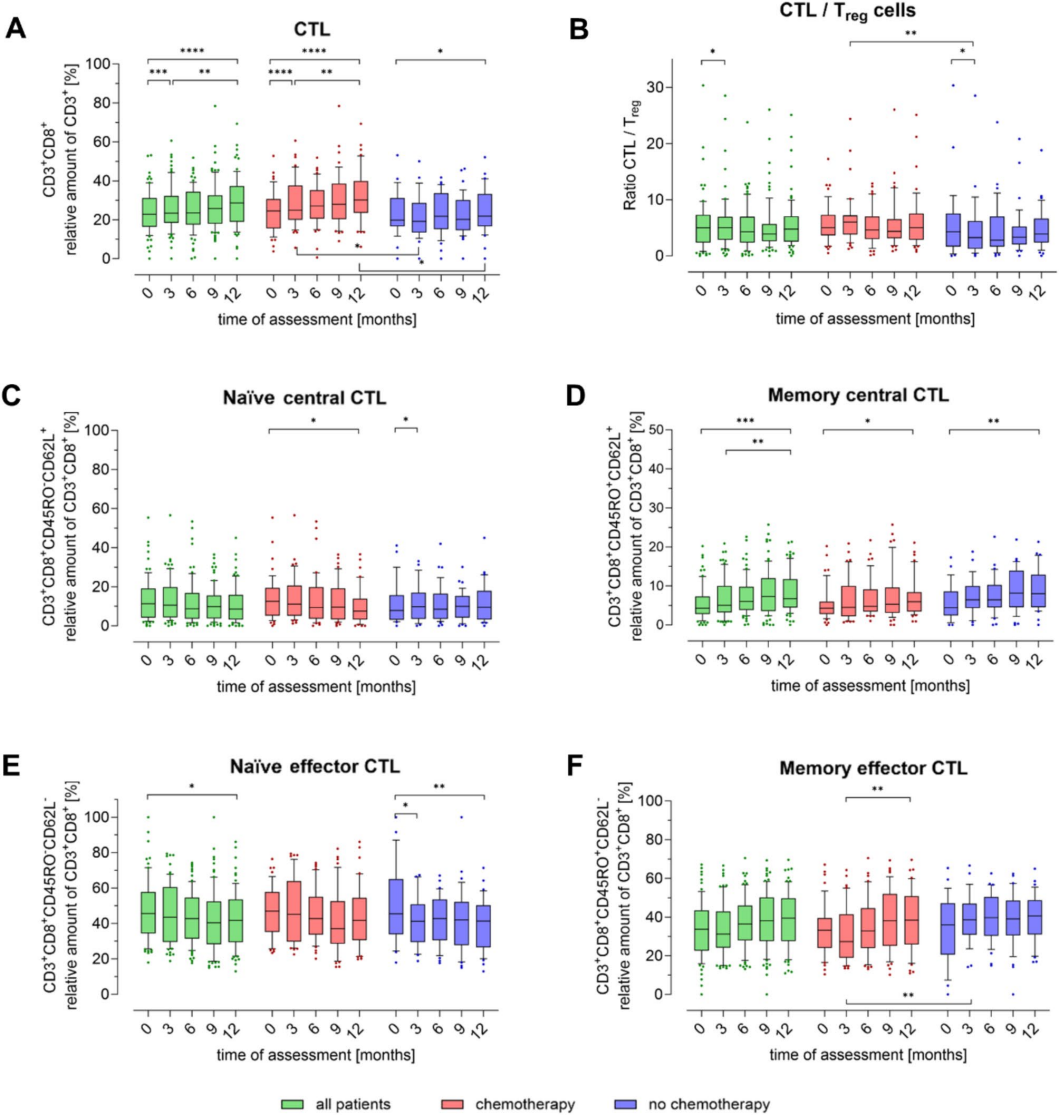
Peripheral blood subpopulations of cytotoxic T cells in breast cancer patients during one year. The proportion of T cell subpopulations (**A**) cytotoxic T cells (CTL, CD3 + CD8+), (**B**) shows CTL/Treg ratio over time (**C**) naïve central CTL (CD3 + CD8 + CD45RO-CD62L+), (**D**) memory central CTL (CD3 + CD8 + CD45RO + CD62L+), (**E**) naïve effector CTL (CD3 + CD8 + CD45RO + CD62L-) and (**F**) memory effector CTL (CD3 + CD8 + CD45RO + CD62L-) in relation to the parent cell population was determined quarterly by flow cytometry. The first measurement (baseline, 0 months) was performed after diagnosis and before initiation of therapy. Boxes extend from the 10th to the 90th percentiles. Points below and above the whiskers are drawn as individual data points. * *p* < 0.05, ** *p* < 0.01, *** *p* < 0.005, **** *p* < 0.001, therapy group differences were assessed using two-tailed Mann Whitney U test, differences over time within one group were assessed using two-tailed Wilcoxon matched-paired signed rank test

As shown in Fig. 3A, a contrasting pattern was observed in cytotoxic T lymphocytes (CTLs, CD3 + CD8+), particularly in the CHT group, where the number of CTLs increased continuously over 12 months. In the NCHT group, there was an initial slight decrease at 3 months, followed by a significant increase at 12 months (*p* < 0.05). However, the CTL levels were significantly lower in the NCHT group compared to the CHT group at both 3 and 12 months. Notably, there are no significant changes in the CTL/Treg ratio within the CHT group during the observation period (Fig. 3B). At three months, the CTL/Treg ratio in the NCHT group is significantly reduced compared to both the baseline (*p* < 0.05) and the CHT group at three months (*p* < 0.01). In the CTL subgroups, naïve central CTLs (CD3 + CD8 + CD45RO-CD62L+, Fig. 3C) were significantly reduced after 12 months in the CHT group, whereas they showed an increase in the NCHT group after 3 months. There was also a significant increase in memory central CTLs (CD3 + CD8 + CD45RO + CD62L+, Fig. 3D) at 12 months in the NCHT group, which was accompanied by a decrease in naïve effector CTLs (CD3 + CD8 + CD45RO-CD62L-, Fig. 3E, *p* < 0.01 respectively). The notable reduction in naïve effector CTLs was accompanied by an increase in the proportion of memory effector CTLs (CD3 + CD8 + CD45RO + CD62L-, Fig. 3F), which was most pronounced within the CHT group. Consequently, similar to the overall CTL population, significant differences between the CHT and NCHT groups were observed at both the 3-month and 12-month time points (Fig. 3A, *p* < 0.05).

**Fig. 4:**
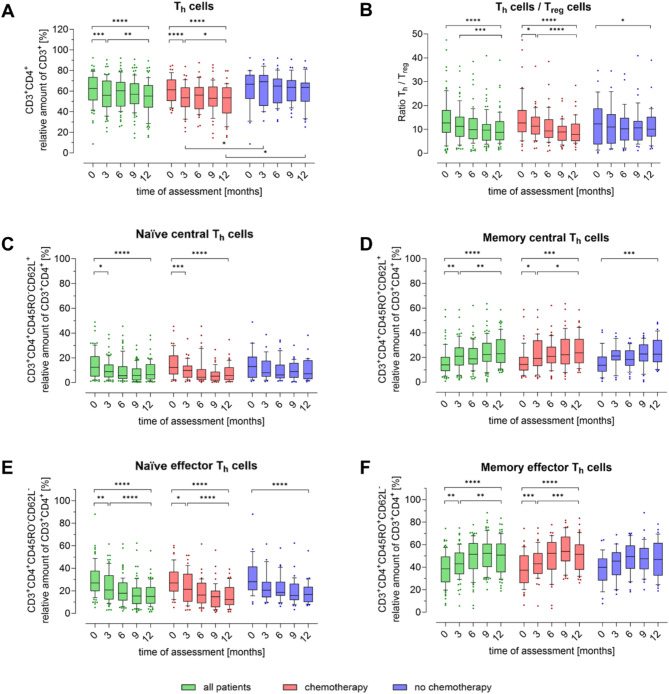
Peripheral blood subpopulations of T helper cells in breast cancer patients during one year. The proportion of T cell subpopulations (**A**) T helper cells (Th, CD3 + CD4+), (**B**) shows the Th/Treg ratio, (**C**) naïve central Th cells (CD3 + CD4 + CD45RO-CD62L+), (**D**) memory central Th cells (CD3 + CD4 + CD45RO + CD62L+), (**E**) naïve effector Th cells (CD3 + CD4 + CD45RO + CD62L-) and (**F**) memory effector Th cells (CD3 + CD4 + CD45RO + CD62L-) in relation to the parent cell population was determined quarterly by flow cytometry. The first measurement (baseline, 0 months) was performed after diagnosis and before initiation of therapy. Boxes extend from the 10th to the 90th percentiles. Points below and above the whiskers are drawn as individual data points. * *p* < 0.05, ** *p* < 0.01, *** *p* < 0.005, **** *p* < 0.001, therapy group differences were assessed using two-tailed Mann Whitney U test, differences over time within one group were assessed using two-tailed Wilcoxon matched-paired signed rank test

A similar finding emerged regarding the differences between CHT and NCHT for Th cells (CD3 + CD4+, Fig. 4A): a significant reduction in Th cells over time in all patients was primarily driven by the CHT group. The Th cell count was significantly higher in the NCHT group compared to the CHT group at the 3-month and 12-month time points (*p* < 0.05). In addition, the ratio of Th to Treg cells decreased significantly more in the CHT group (*p* < 0.001) than in the NCHT group (*p* < 0.05) at 12 months (Fig. 4B). The changes over time in the Th cell subpopulations (Fig. 4), with respect to the maturation markers CD45RO and CD62L, were more pronounced than in the CTL subpopulations (Fig. 3). Despite the stability of the parental Th cell population in the NCHT group, changes in Th subpopulations over time were evident: There was a significant increase in memory central Th cells (CD3 + CD4 + CD45RO + CD62L+, Fig. 4D) accompanied by a reduction in naïve effector Th cells (CD3 + CD4 + CD45RO-CD62L-, Fig. 4E). Similar trends were observed in the CHT group, with a significant reduction in naïve central Th cells (CD3 + CD4 + CD45RO-CD62L+, Fig. 4C) and an increase in memory effector Th cells (CD3 + CD4 + CD45RO + CD62L-, Fig. 4F) over time. While the NCHT group showed similar trends, their overall stability was greater.

**Fig. 5:**
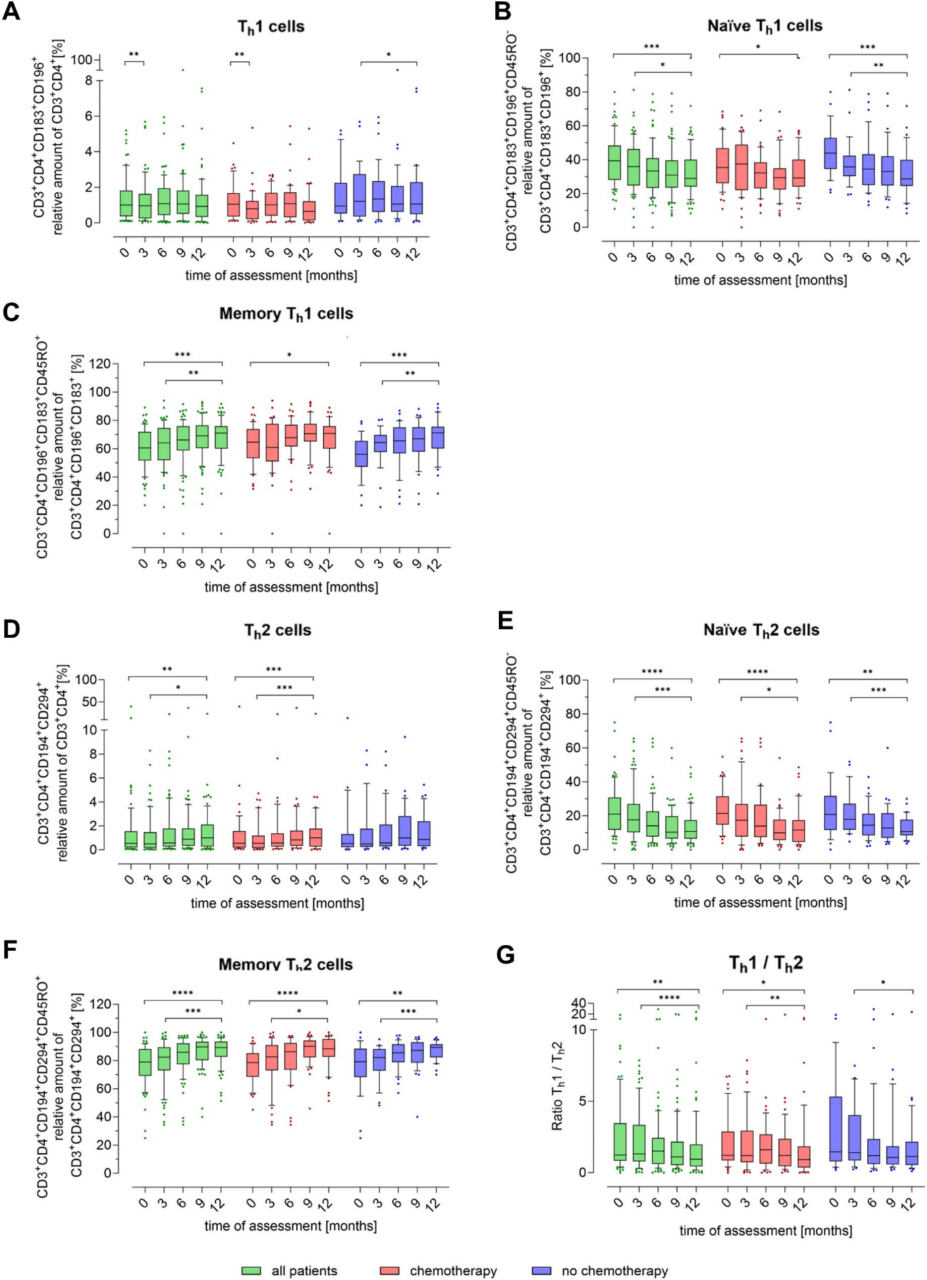
Peripheral blood subpopulations of Th1 and Th2 cells in breast cancer patients during one year. The proportion of T cell subpopulations (**A**) T helper cells 1 (Th1, CD3 + CD4 + CD183 + CD196+), (**B**) naïve Th1 (CD3 + CD4 + CD183 + CD196 + CD45RO-), (**C**) memory Th1 (CD3 + CD4 + CD183 + CD196 + CD45RO+), (**D**) T helper cells 2 (Th2, CD3 + CD4 + CD194 + CD294+), (**E**) naïve Th2 (CD3 + CD4 + CD194 + CD294 + CD45RO-) and (**F**) memory Th2 (CD3 + CD4 + CD194 + CD294 + CD45RO+) in relation to the parent cell population was determined quarterly by flow cytometry. The first measurement (baseline, 0 months) was performed after diagnosis and before initiation of therapy. (**G**) shows the Th1/Th2 ratio over time. Boxes extend from the 10th to the 90th percentiles. Points below and above the whiskers are drawn as individual data points. * *p* < 0.05, ** *p* < 0.01, *** *p* < 0.005, **** *p* < 0.001, therapy group differences were assessed using two-tailed Mann Whitney U test, differences over time within one group were assessed using two-tailed Wilcoxon matched-paired signed rank test

After three months, the concentration of Th1 cells decreased significantly, primarily in the CHT group, but stabilized at 6 months, although it did not return to baseline levels (Fig. 5A). The subpopulations of naïve Th1 cells (Fig. 5B) decreased significantly over time in all groups, while memory Th1 cells increase significantly (Fig. 5C). In the NCHT group, there were notable differences between 0 and 12 months and between 3 and 12 months in both subpopulations.

Th2 cells increased significantly over time in CHT patients, whereas their levels remained comparatively stable in NCHT patients (Fig. 5D). In both patient groups, there was a decrease in the subpopulation of naïve Th2 cells (Fig. 5E), which is inversely related to the number of memory Th2 cells (Fig. 5F).

Additionally, the Th1/Th2 ratio shows a significant decrease in all patients over time, particularly between 3 and 12 months (Fig. 5G). Descriptive statistics of flow cytometry data are shown in the Supplementary Table S2.

### Changes in cytokine levels over time

Detectable cytokine levels were found in 97.3% (IL-8) to 100% (TNF, IFN-γ, IL-7, IL-12) of all analyzed samples. The cytokine levels for IFN-γ, TNF, IL-7 and IL-12 mostly remained in the one to two-digit pg/mL range, while results for IL-4, IL-8 and IL-10 were more heterogeneous. Similar to the T cell subpopulations, measurable effects on cytokine levels over the course of the first year were generally more pronounced in the CHT group.

**Fig. 6:**
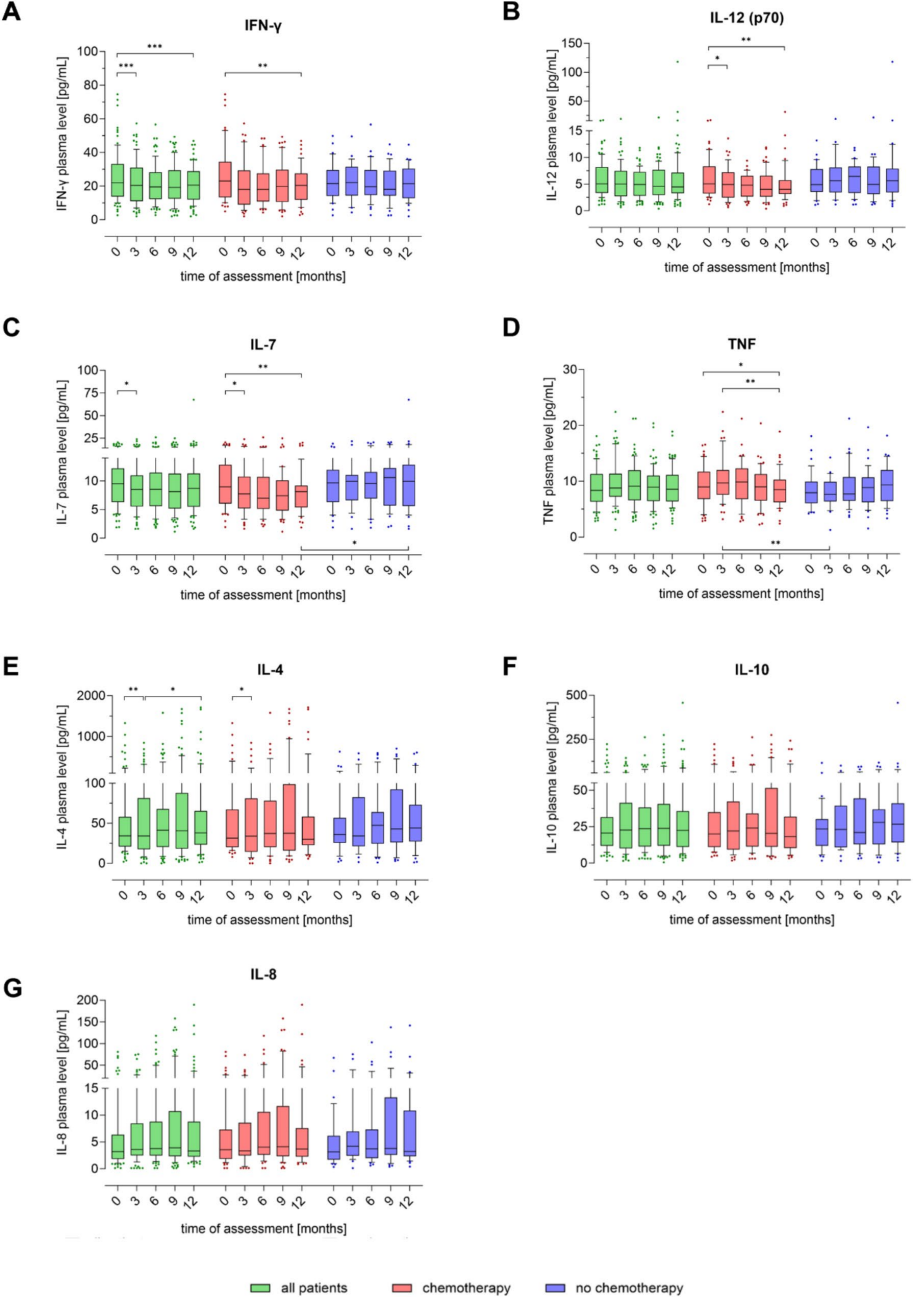
Peripheral blood plasma cytokine levels in breast cancer patients during one year. The amount of (**A**) IFN-γ, (**B**) IL-12, (**C**) IL-7, (**D**) TNF, (**E**) IL-4, (**F**) IL-10 and (**G**) IL-8, was determined quarterly by multiplex cytokine assay (MAGPIX^®^). The first measurement (baseline, 0 months) was performed after diagnosis and before initiation of therapy. Boxes extend from the 10th to the 90th percentiles. Points below and above the whiskers are drawn as individual data points. * *p* < 0.05, ** *p* < 0.01, *** *p* < 0.005, therapy group differences were assessed using two-tailed Mann Whitney U test, differences over time within one group were assessed using two-tailed Wilcoxon matched-paired signed rank test

Pro-inflammatory IFN-γ levels were higher at baseline in all patients compared to levels 3 and 12 months post-diagnosis (*p* < 0.005), a trend also evident in the CHT group (*p* < 0.01, Fig. 6A). In patients receiving chemotherapy, IL-12 levels decreased over the first year after breast cancer diagnosis (*p* < 0.01), while an opposite trend was observed in the NCHT group in the first 6 months post-diagnosis (Fig. 6B). IL-7 levels declined steadily over time, primarily driven by the CHT group (Fig. 6C). In the second half of the first year, IL-7 levels of the CHT group were significantly lower than those in the NCHT group, which showed a more consistent IL-7 trajectory.

While the aforementioned cytokines tended to decrease after the baseline measurement, the median pro-inflammatory TNF levels peaked in the CHT group after 3 months (Fig. 6D). At this time point, TNF were also significantly higher in the CHT group than in the NCHT group (*p* < 0.01). Subsequently, the TNF level in chemotherapy patients decreased again and was lower than baseline measurement after 12 months (*p* < 0.05).

It is notable that IL-4 which is associated with Th2 cells, showed the most heterogeneous effects both inter-individually and temporally (Fig. 6E). From baseline to 3 months post-diagnosis, the 10-90th percentile generally increased, although the median IL-4 level remained almost constant. However, there was a significant decline in IL-4 levels in the CHT group (*p* < 0.05), but not in the NCHT group, over the same period.

For most patients, across both groups and over the entire time course, the plasma levels for anti-inflammatory interleukin IL-10 (Fig. 6F) and chemokine IL-8 remained at basal levels (Fig. 6G). Nevertheless, upper values one or two powers of ten above the median IL-10 and IL-8 levels were observed, although there were no statistically significant differences in terms of the time course or the use of chemotherapy.

Descriptive statistics of Multiplex Immunoassay data are shown in the Supplementary Table S3.

### Conjunction of T cell dynamics with the outcome of the patients undergoing neoadjuvant chemotherapy

In patients undergoing neoadjuvant chemotherapy it is possible to measure therapeutic responses. The response to neoadjuvant chemotherapy gives valuable prognostic information, as pathologic complete response (pCR, ypT0/is, ypN0) is associated with better recurrence-free and overall survival [42, 43]. In the BEGYN-1 study, a total of 35 patients underwent neoadjuvant chemotherapy, and 12 patients obtained a pathologic complete response (pCR) (Supplementary Table S4). We analyzed whether there were differences in the levels of cell populations or cytokines at any time point of the study comparing the pCR group to the non-pCR group.

**Fig. 7:**
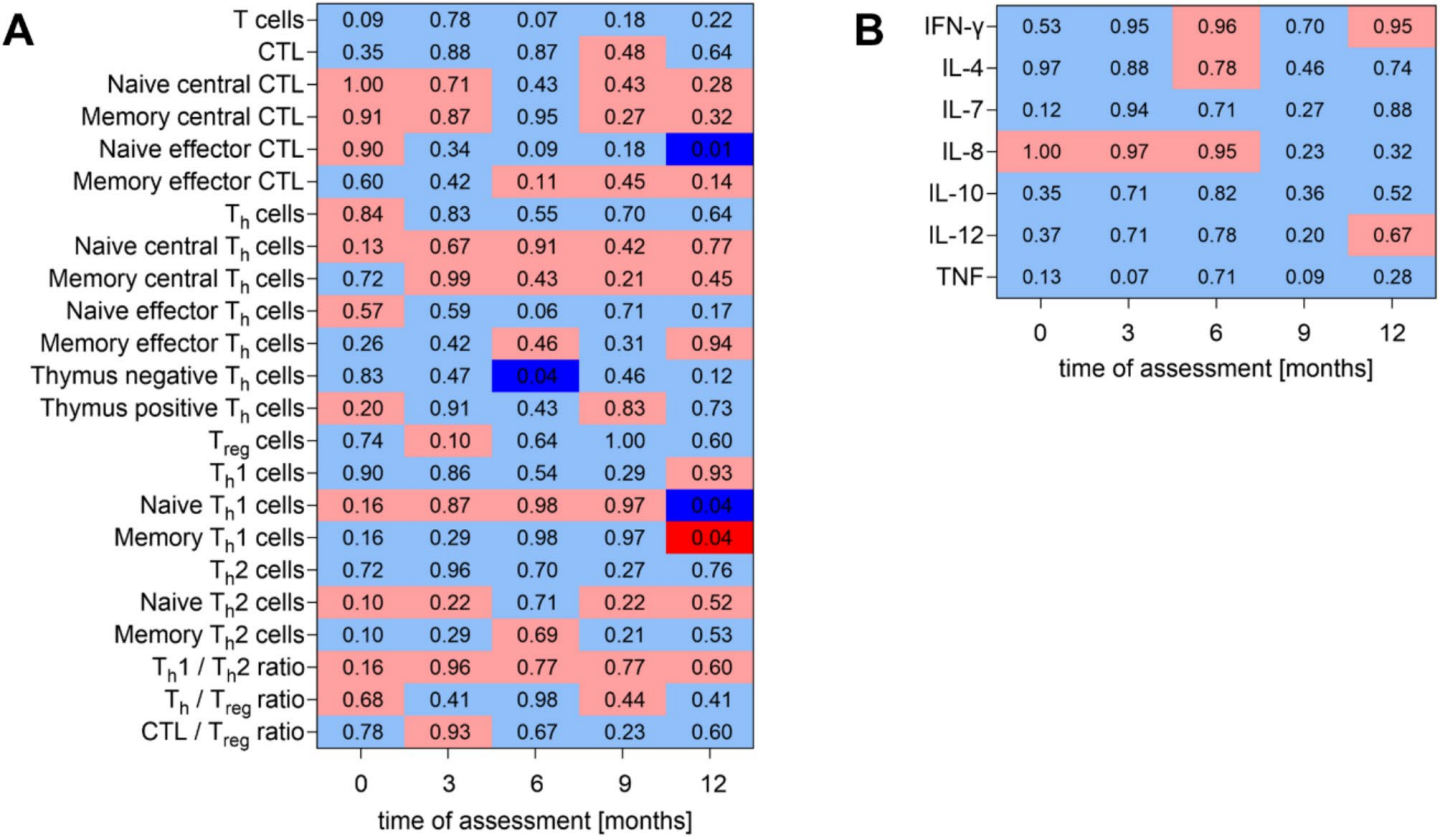
Dynamics of T cell subpopulations and plasma cytokines: pCR vs. non-pCR. A total of 35 breast cancer patients underwent neoadjuvant chemotherapy during BEGYN-1 study. 12 patients obtained a pathologic complete response (pCR) which was not obtained by 23 patients (non-pCR). The differences in the dynamics of (**A**) T cell subpopulations (relative amount of parent population) and (**B**) plasma cytokines were assessed between pCR and non-pCR using two-tailed Mann Whitney U test. Individual p-value are given in corresponding cells of the matrix, which is connected to the categorical color code (light red: median & rank higher in the pCR group with *p* > 0.05, dark red: median & rank higher in the pCR group with *p* < 0.05, light blue: median & rank lower in the pCR group with *p* > 0.05, dark blue: median & rank lower in the pCR group with *p* < 0.05). Descriptive statistics in given in Table S5

Patients who obtained a pCR tend to possess a lower number of T cells (CD3+) than non-pCR patients. This applies to the entire study period with the exception of 3 months after study initiation (Fig. 7A). Although no significant differences in the amount of Th1 and Th2 cells were found between the pCR and non-pCR patient groups, there were clear differences found among their subpopulations: Before any intervention, there is a tendency for pCR patients to have both predominant naïve Th1 and naïve Th2 cells compared to their memory partners. At the end of the observation period, the pCR group displayed significantly more Th1 memory cells compared to the non-pCR patients, whereas the ratio of naïve to memory Th2 cells harmonized. The lack of expression of the memory marker CD45RO in the group of pCR patients also appears to be apparent for the cell populations of thymus negative Th cells but also for both naïve effector Th and naïve effector CTL, mainly at 6 and 12 months after the start of the study.

Both IL-7 and TNF tend to be reduced in pCR patients at the beginning of the observation period (Fig. 7B). While the expression of IL-7 harmonized between the pCR and non-pCR groups over the course of the year, the tendency for TNF persisted.

Descriptive statistics, grouped by outcome (pCR and non-pCR), of flow cytometry data are shown in the Supplementary Table S5 and of Multiplex Immunoassay data in the Supplementary Table S6.

Since different systemic therapies could have an impact on the immune cell populations, we also performed a subgroup analysis in which all patients receiving anti-HER2 agents, Atezolizumab, Cepecitabine or Abemaciclib were excluded. This resulted in a reduction of CHT patients from *n* = 54 to *n* = 25. However, the analyses (Figure S5 - Figure S9) show essentially the same picture as when the entire CHT patient population is included.

## Discussion

The BEGYN-1 study [35] has a holistic approach to investigate correlations between physical activity [36], body composition, quality of life and different laboratory values as e.g. Vitamin D [37, 38], and Selenium [39], and the immune system. The aim of the BEGYN-1 study was to develop personalized recommendations to improve the quality of life, lifestyle and prognosis of breast cancer patients and to identify potential prognostic biomarkers [36–38]. Some results have already been published; others still have to be evaluated. For example, we were able to show that wearing a fitness tracker and using a diary to document physical activity can help mobilize patients [36]. Notwithstanding the intention to conduct additional evaluations in the future, including those pertaining to B cells, it is imperative to establish further correlations between T cells/interleukins and patient activity, as well as between patient age and serum Vitamin D levels. In the present study, we characterize the T cell immune response on the cellular and cytokine level in non-metastasized breast cancer patients from the BEGYN-1 study over a 12-month period in relation to the therapy applied. Compared to other studies known to us, the BEGYN-1 study includes the highest number of non-metastatic breast cancer patients whose T cell immune response was analyzed on a cellular and cytokine level during one year after diagnosis [44–49].

We found that the number of Th cells decreased significantly in patients after chemotherapy and was still below the baseline level after 12 months. This reflects the cytotoxic effect on these cells, which include thymus negative Th cells, thymus positive Th cells, naïve central Th cells, memory central Th cells and naïve effector Th cells. This could temporarily hamper the immune defense and lead to e.g. poor vaccine responses, increased risks of infections and malignancies [50, 51]. Furthermore, we found an increase in Th memory cells after chemotherapy. These results confirm and extend results that were previously published by Verma et al. [21]. In this study T cell subsets were assessed in 88 patients with primary breast cancer before chemotherapy and at time points ranging from two weeks to nine months after completion of chemotherapy. The number of circulating T cells reached a nadir two weeks after chemotherapy and remained below baseline nine months after chemotherapy. Moreover, we found that Th cell subsets showed divergent dynamics in the CHT and NCHT groups: While the numbers of naïve central and naïve effector Th cells declined only in the CHT group, naïve effector CTL declined both in the CHT and the NCHT groups. The reduction in naïve T cells can be attributed to chemotherapy-induced thymus atrophy, which is linked to decreased production of these cells [52, 53]. However, in younger patients, there may be a transient enlargement of the thymus following treatment, which is associated with faster recolonization by naïve Th cells, although this should be further investigated in the context of age based on data from the BEGYN-1 study [54]. The decline in naïve Th cells might also result from mitochondrial damage induced by chemotherapeutic agents, leading to increased apoptosis in rapidly proliferating cells like activated naïve T cells. Additionally, naïve T cells are particularly vulnerable to the cytotoxic effects of chemotherapy [55, 56]. The observed increase in average memory Th cells, indicated by the CD45RO marker, could be due to chemotherapy-induced tumor cell death, which releases tumor antigens [3]. These antigens, when presented by APCs, activate naïve Th cells into effector cells, some of which differentiate into memory Th cells. The observed decrease in CD31 + thymus positive and CD31- thymus negative naïve Th cells in patients undergoing chemotherapy aligns with previous findings showing a reduction in CD31 + Th cells after two weeks of treatment [21]. These results suggest that chemotherapy may impair the frequency of recent thymic emigrants, explaining the decline in both CD31 + thymus positive and CD31- thymus negative Th cells. Given that full immune recovery post-chemotherapy depends on the thymic production of new CD31 + thymus positive Th cells to replenish peripheral cells, it is crucial to understand how cancer-related factors and lifestyle choices impact these cells to optimize treatment outcomes [57].

Interestingly, within the group undergoing neoadjuvant chemotherapy, the dynamics in the reduction of the two cell populations differed when comparing pCR and non-pCR patients especially in CD31- thymus negative Th cells with a tendency towards lower frequencies in pCR patients. Further research is needed to examine these dynamics in detail. Notably, the cytokine milieu also affects CD31 + and CD31- naïve T cells and thymopoiesis. In example, the marker cytokine IL-7 plays a critical role in the early stages of T cell development and thymopoiesis [58, 59]. Accordingly, we found a decrease in IL-7 levels and CD31 + naïve Th cells in CHT patients. The lower IL-7 levels may originate from effects on its numerous production sites in stromal, epithelial, and fibroblastic cells of both lymphoid and non-lymphoid tissues [60, 61].

Verma et al. also monitored changes in circulating immune cells over time in breast cancer patients as part of their study, and observed a transient decrease in CTL over approximately two weeks after chemotherapy [21]. In our study, the first blood sample was taken three months after baseline, which does not allow direct comparison with the dynamics observed by Verma et al. CTL numbers were increased after three months in non-chemotherapy patients but unaltered in patients that received chemotherapy. Intriguingly, the number of CTL was greater after one year than at the baseline, suggesting that breast cancer and antineoplastic therapy might have a long-term impact on the immune system. Moreover, we extended these findings by studying CTL subpopulations: We found that this increase is primarily due to the proliferation of memory CTL, which slightly offsets the decline in naïve CTL. In addition, the study revealed naïve effector CTL to be significantly reduced in pCR patients compared to non-pCR patients. Possibly, naïve CTL were exposed to tumor-derived antigen during chemotherapy, leading to activation towards CD45RO positive memory cells [62].

In addition, the concentration of Treg cells and the change in the ratio of CTLs to Tregs or Th cells to Tregs in the surrounding tumor or PBMC was described as a possible biomarker for evaluating the efficacy of therapy or the prognosis of cancer patients [66–70]. In our study, we found that the Th/Treg ratio decreased significantly more in the CHT group at 12 months (*p* < 0.001) than in the NCHT group (*p* < 0.05). The CTL/Treg ratio was significantly reduced in the NCHT group only after 3 months compared to the CHT group. No correlation was observed between Treg levels or CTL/Treg or Th/Treg ratios and prognosis in the context of pCR group allocation.

We also studied the plasma cytokine signatures that are associated with the T cell response during the one year period of the BEGYN-1 study. In particular, soon after strong type 1 immune responses are triggered, e.g. by tissue necrosis followed by secretion of IFN-y and IL-12, a number of T cell inhibitory mechanisms are activated, e.g. by secretion of IL-4 and IL-9 to avoid an overshooting response [71, 72].

In our BEGYN-1 study patients, the number of Th1 cells decreased after 3 months, i.e. after the majority of CHT patients had received their first cycles of therapy. Subsequently Th1 cells are subject to some variations and eventually remain below the baseline after 12 months. In harmony with this finding, the decrease in Th1 cell numbers was paralleled by a decline in the plasma concentrations of IL-12 levels, a key mediator of the Th1-type immune response and of IFN-y, the pro-inflammatory marker cytokine which is preferentially secreted by Th1 cells. Reductions in the Th1/Th2 ratio during the 12 months of the BEGYN-1 study are not only due to the reduction in Th1 cells but also to the increase in Th2 cells both in the CHT and NCHT groups. Probably, chemotherapy primarily affects the development of Th1 cells and the differentiation of naïve Th2 cells into memory cells. The reduction of antitumor mediators after 3 months occurs at a time when in many cases the tumor has been resected and endocrine and/or antibody therapy is ongoing.

To better understand the underlying mechanisms contributing to these changes in Th1 and Th2 cells, plasma concentrations of various cytokines associated with Th1 and Th2 responses were measured over the 12-month period of the study. The pro-inflammatory cytokines IFN-γ and TNF are commonly associated with Th1 responses, while the anti-inflammatory cytokines IL-4 and IL-10 are predominantly produced by Th2 cells. This is of crucial importance as the Th1/Th2 balance in breast cancer shifts towards Th2 dominance, leading to more tumor-promoting immune status [73]. However, the shift in the Th1/Th2 balance during the course of the study could also be attributable to the resolution of the initial acute inflammation caused by the tumor, antineoplastic medication, radiation and surgery. Notably, various definitions were proposed for Th1 and Th2 cells and other Th cell subsets, each of which is associated with diseases, infections, or similarity to other T cells due to specific properties [74–79]. Since the majority of T cells in this study are not classified as Th1 or Th2 cells, it would be interesting to investigate the direct properties and functions of defined Th1 and Th2 cells at the molecular level.

Both TNF and IFN-γ are secreted by natural killer (NK) cells, while IL-10 can increase the metabolism of NK cells and thus their effectiveness [80, 81]. TNF as a pro-inflammatory cytokine, is mainly produced by activated macrophages, T lymphocytes and natural killer cells, but is also found in the microenvironment of tumors and showed level modulations especially in the group of CHT patients. Interestingly, through exogenous administration, TNF can also play a dual role in anti-cancer therapy [82, 83]. For an improved interpretation of the cytokine data, the secreting cell levels would be important.

To our knowledge, this is the largest cohort of patients that were followed up over course of one year after the study of non-metastasized breast cancer. The strengths of the study include the first regular, thorough and very detailed investigation of T-cell subpopulations and T-cell-associated cytokines in a large cohort of breast cancer patients in association with therapeutic interventions.

However, some limitations of our study must be taken into account: Although this is one of the largest cohorts of breast cancer patients followed over one year after diagnosis, the study is underpowered to give detailed insights into the influences of individual chemotherapy agents, side medication, or individual factors, such as intercurrent infections, genetic disposition, microbiota, lifestyle, and others [5, 50]. Due to the personalized approach in breast cancer treatment increases, patients may have undergone different therapies at different time points. Moreover, some patients might have undergone discontinuation or dose reduction of their medications due to individual factors. We illustrate this complexity of treatment patterns in Fig. 1, Figure S3 and Figure S4. However, the number of patients receiving surgery and radiotherapy, as well as endocrine therapy, was similar between the two groups (chemotherapy versus no chemotherapy). It can be concluded that other than the variable of chemotherapy (yes/no), there should be no substantial influence on the differences observed in T cell subpopulations and plasma cytokines.

In the future, it would be valuable to carry out additional experiments, such as gene expression analyses or single cell analyses, in order to reveal molecular mechanisms of antineoplastic therapies. It would also be interesting to evaluate patient’s outcome several years later and to correlate the immunological data in order to identify prognostic biomarkers.

## Hypothesis

The BEGYN-1 study provides insights into the dynamics of T cell responses during the first year after the diagnosis of non-metastasized breast cancer. Ongoing modulations in the T-cell mediated immune response might remain detectable 12 months after diagnosis and initiation of anti-cancer therapies. The type of treatment (chemotherapy versus no chemotherapy) might induce divergent changes in the immune profile, particularly affecting a shift from Th1 towards Th2 bias, an increase in circulating CTL during the 12 months study period and the expression of memory markers. Plasma cytokine expressions appear to be in harmony with the observed dynamics of T cell subpopulations. To identify further potentially prognostic biomarkers, it would be valuable to investigate B cell immunity and innate immunity as well as to evaluate the patient’s longterm outcome, and to explore correlations with potential immunomodulating factors.

## Electronic supplementary material

Below is the link to the electronic supplementary material.

Supplementary Material 1

Supplementary Material 2: Additional file 1: Table S1 Flow cytometry antibodies.

Supplementary Material 3: Table S2. Descriptive statistics of flow cytometry data (treatment).

Supplementary Material 4: Table S3. Descriptive statistics of multiplex immunoassay data (treatment).

Supplementary Material 5: Table S4: Patient undergoing neoadjuvant chemotherapy characteristics.

Supplementary Material 6: Table S5. Descriptive statistics of flow cytometry data (outcome).

Supplementary Material 7: Table S6. Descriptive statistics of multiplex immunoassay data (outcome).

Supplementary Material 8: Figure S1. Gating strategy of T cell subpopulations (panel 1).

Supplementary Material 9: Figure S2. Gating strategy of T cell subpopulations (panel 2).

Supplementary Material 10: Figure S3: Individual treatment patterns of CHT patients.

Supplementary Material 11: Figure S4: Individual treatment patterns of NCHT patients.

Supplementary Material 12: Figure S5: Peripheral blood T cell populations in breast cancer patients receiving chemotherapy without potentially immunomodulatory therapy during one year.

Supplementary Material 13: Figure S6: Peripheral blood subpopulations of cytotoxic T cells in breast cancer patients receiving chemotherapy without potentially immunomodulatory therapy during one year

Supplementary Material 14: Figure S7: Peripheral blood subpopulations of T helper cells in breast cancer patients receiving chemotherapy without potentially immunomodulatory therapy during one year.

Supplementary Material 15: Figure S8: Peripheral blood subpopulations of Th1 and Th2 cells in breast cancer patients receiving chemotherapy without potentially immunomodulatory therapy during one year.

Supplementary Material 16: Figure S9: Peripheral blood plasma cytokine levels in breast cancer patients receiving chemotherapy without potentially immunomodulatory therapy during one year.

## Data Availability

The datasets generated during the current study are available from the corresponding author on reasonable request.

## Abbreviations

APC: Antigen Presenting Cell
AO/PI: Acridine Orange and Propidium Iodide
BEGYN: Influence of Physical Activity in Breast Cancer Patients on Physiological and Psychological Parameters and on Biomarkers
CAR: Chimeric Antigen Receptor
CHT: Chemotherapy
CS&T: Cytometer Setup and Tracking Tool
CTL: Cytotoxic T Lymphocyte
D-PBS: Dulbecco’s Phosphate-Buffered Saline
DMSO: Dimethylsulfoxid
EDTA: Ethylenediamine Tetraacetic Acid
FBS: Fetal Bovine Serum
IFN: Interferon
IL: Interleukin
NCHT: No Chemotherapy
Non-pCR: No Pathologic Complete Response
PBMC: Peripheral Blood Mononuclear Cell
pCR: pathologic Complete Response
TCR: T Cell Receptor
Th: T Helper
Treg: Regulatory T cell
TIL: Tumor-Infiltrating Lymphocyte
TNF: Tumor Necrosis Factor

## References

[1] Sung H, et al. Global cancer statistics 2020: GLOBOCAN estimates of incidence and mortality worldwide for 36 cancers in 185 countries. CA Cancer J Clin. 2021;71:209–49. 33538338 10.3322/caac.21660

[2] Arnold M, et al. Current and future burden of breast cancer: global statistics for 2020 and 2040. Breast Edinb Scotl. 2022;66:15–23. 10.1016/j.breast.2022.08.010 PMC9465273 36084384

[3] Emens LA. Chemotherapy and tumor immunity: an unexpected collaboration. Front Biosci J Virtual Libr. 2008;13:249–57. 10.2741/2675 PMC3086378 17981543

[4] Huang H, et al. The Immunomodulatory effects of endocrine therapy in breast cancer. J Exp Clin Cancer Res. 2021;40:19. 33413549 10.1186/s13046-020-01788-4 PMC7792133

[5] Garner H, de Visser KE. Immune crosstalk in cancer progression and metastatic spread: a complex conversation. Nat Rev Immunol. 2020;20:483–97. 32024984 10.1038/s41577-019-0271-z

[6] Schreiber RD, Old LJ, Smyth MJ. Cancer immunoediting: integrating immunity’s roles in cancer suppression and promotion. Science. 2011;331:1565–70. 21436444 10.1126/science.1203486

[7] Smyth MJ, Dunn GP, Schreiber RD. Cancer immunosurveillance and immunoediting: the roles of immunity in suppressing tumor development and shaping tumor immunogenicity. Adv Immunol. 2006;90:1–50. 16730260 10.1016/S0065-2776(06)90001-7

[8] Zikos TA, Donnenberg AD, Landreneau RJ, Luketich JD, Donnenberg VS. Lung T-cell subset composition at the time of surgical resection is a prognostic indicator in non-small cell lung cancer. Cancer Immunol Immunother. 2011;60:819–27. 21373990 10.1007/s00262-011-0996-4 PMC4154502

[9] Zareinejad M, Mehdipour F, Roshan-Zamir M, Faghih Z, Ghaderi A. Dual functions of T lymphocytes in breast carcinoma: from immune protection to orchestrating tumor progression and metastasis. Cancers. 2023;15:4771. 37835465 10.3390/cancers15194771 PMC10571747

[10] Tietscher S, et al. A comprehensive single-cell map of T cell exhaustion-associated immune environments in human breast cancer. Nat Commun. 2023;14:98. 36609566 10.1038/s41467-022-35238-w PMC9822999

[11] Knutson KL, Disis ML, Augmenting T. Helper cell immunity in cancer. Curr Drug Targets - Immune Endocr Metab Disord. 2005;5:365–71. 10.2174/156800805774913006 16375690

[12] Leong PP, et al. Phenotyping of lymphocytes expressing regulatory and effector markers in infiltrating ductal carcinoma of the breast. Immunol Lett. 2006;102:229–36. 16246429 10.1016/j.imlet.2005.09.006

[13] Sun Y-P, Ke Y-L, Li X. Prognostic value of CD8^+^ tumor–infiltrating T cells in patients with breast cancer: A systematic review and meta–analysis. Oncol Lett. 2023;25:1–11. 36589661 10.3892/ol.2022.13625 PMC9773320

[14] Liu Q, Sun Z, Chen L. Memory T cells: strategies for optimizing tumor immunotherapy. Protein Cell. 2020;11:549–64. 32221812 10.1007/s13238-020-00707-9 PMC7381543

[15] Georgiannos SN, Renaut A, Goode AW, Sheaff M. The immunophenotype and activation status of the lymphocytic infiltrate in human breast cancers, the role of the major histocompatibility complex in cell-mediated immune mechanisms, and their association with prognostic indicators. Surgery. 2003;134:827–34. 14639362 10.1016/s0039-6060(03)00292-7

[16] Wong PY, Staren ED, Tereshkova N, Braun DP. Functional analysis of Tumor-Infiltrating leukocytes in breast cancer patients. J Surg Res. 1998;76:95–103. 9695747 10.1006/jsre.1998.5301

[17] Helal TEA, Ibrahim EAA, Alloub A. I. A. Immunohistochemical analysis of tumor-infiltrating lymphocytes in breast carcinoma: relation to prognostic variables. Indian J Pathol Microbiol. 2013;56:89. 24056641 10.4103/0377-4929.118676

[18] Ruffell B, et al. Leukocyte composition of human breast cancer. Proc Natl Acad Sci. 2012;109:2796–801. 21825174 10.1073/pnas.1104303108 PMC3287000

[19] Reome JB, Hylind JC, Dutton RW, Dobrzanski MJ. Type 1 and type 2 tumor infiltrating effector cell subpopulations in progressive breast cancer. Clin Immunol. 2004;111:69–81. 15093554 10.1016/j.clim.2003.11.013

[20] Faghih Z, Deihimi S, Talei A, Ghaderi A, Erfani N. Analysis of T cell receptor repertoire based on Vβ chain in patients with breast cancer. Cancer Biomark. 2018;22:733–45. 29945345 10.3233/CBM-181295 PMC13078503

[21] Verma R, et al. Lymphocyte depletion and repopulation after chemotherapy for primary breast cancer. Breast Cancer Res BCR. 2016;18:10. 26810608 10.1186/s13058-015-0669-x PMC4727393

[22] Jiang M, et al. T-Cell subset counts in peripheral blood can be used as discriminatory biomarkers for diagnosis and severity prediction of coronavirus disease 2019. J Infect Dis. 2020;222:198–202. 32379887 10.1093/infdis/jiaa252 PMC7239156

[23] O’Rourke RW, et al. Alterations in T-Cell subset frequency in peripheral blood in obesity. Obes Surg. 2005;15:1463–8. 16354528 10.1381/096089205774859308

[24] Bates GJ, et al. Quantification of regulatory T cells enables the identification of high-risk breast cancer patients and those at risk of late relapse. J Clin Oncol Off J Am Soc Clin Oncol. 2006;24:5373–80. 10.1200/JCO.2006.05.9584 17135638

[25] Wang L, et al. Connecting blood and intratumoral Treg cell activity in predicting future relapse in breast cancer. Nat Immunol. 2019;20:1220–30. 31285626 10.1038/s41590-019-0429-7 PMC8802768

[26] Oshi M, et al. Abundance of regulatory T cell (Treg) as a predictive biomarker for neoadjuvant chemotherapy in Triple-Negative breast cancer. Cancers. 2020;12:3038. 33086518 10.3390/cancers12103038 PMC7603157

[27] Zamarron BF, Chen W. Dual roles of immune cells and their factors in cancer development and progression. Int J Biol Sci. 2011;7:651–8. 21647333 10.7150/ijbs.7.651 PMC3107473

[28] Tsuda B, et al. B-cell populations are expanded in breast cancer patients compared with healthy controls. Breast Cancer. 2018;25:284–91. 29204848 10.1007/s12282-017-0824-6 PMC5906508

[29] Gonzalez H, Hagerling C, Werb Z. Roles of the immune system in cancer: from tumor initiation to metastatic progression. Genes Dev. 2018;32:1267–84. 30275043 10.1101/gad.314617.118 PMC6169832

[30] Wang Z, et al. Targeting glutaminolysis: new perspectives to understand cancer development and novel strategies for potential target therapies. Front Oncol. 2020;10:589508. 33194749 10.3389/fonc.2020.589508 PMC7649373

[31] Vang AR, et al. Plasma cytokines/chemokines as predictive biomarkers for lymphedema in breast cancer patients. Cancers. 2023;15:676. 36765631 10.3390/cancers15030676 PMC9913278

[32] Invernizzi M, et al. Quality of life interventions in breast cancer survivors: state of the Art in targeted rehabilitation strategies. Anticancer Agents Med Chem. 2022;22:801–10. 34151769 10.2174/1871520621666210609095602

[33] Schmidt T, et al. Influence of physical activity on the immune system in breast cancer patients during chemotherapy. J Cancer Res Clin Oncol. 2018;144:579–86. 29305709 10.1007/s00432-017-2573-5 PMC11813291

[34] van der Leeden M, et al. Tailoring exercise interventions to comorbidities and treatment-induced adverse effects in patients with early stage breast cancer undergoing chemotherapy: a framework to support clinical decisions. Disabil Rehabil. 2018;40:486–96. 28054496 10.1080/09638288.2016.1260647

[35] Zemlin C et al. Longitudinal Assessment of Physical Activity, Fitness, Body Composition, Immunological Biomarkers, and Psychological Parameters During the First Year After Diagnosis in Women With Non-Metastatic Breast Cancer: The BEGYN Study Protocol. *Front. Oncol.* 11, 762709 (2021). 10.3389/fonc.2021.762709 PMC8560964 34737966

[36] Zemlin C, et al. Improved awareness of physical activities is associated with a gain of fitness and a stable body weight in breast cancer patients during the first year of antineoplastic therapy: the BEGYN-1 study. Front Oncol. 2023;13:1198157. 37637039 10.3389/fonc.2023.1198157 PMC10456044

[37] Zemlin C, et al. Course of vitamin D levels in newly diagnosed Non-Metastatic breast cancer patients over one year with quarterly controls and substitution. Nutrients. 2024;16:854. 38542765 10.3390/nu16060854 PMC10975236

[38] Zemlin C, et al. Prevalence and relevance of vitamin D deficiency in newly diagnosed breast cancer patients: A pilot study. Nutrients. 2023;15:1450. 36986179 10.3390/nu15061450 PMC10056197

[39] Altmayer LA, et al. A plea for monitoring serum selenium levels in breast cancer patients: selenium deficiency is rare during the first year of therapy, and selenium supplementation is associated with elevated risk of overdosing. Nutrients. 2024;16:2134. 38999881 10.3390/nu16132134 PMC11243168

[40] S3-Leitlinie Mammakarzinom. (2021).

[41] Loibl S, et al. Early breast cancer: ESMO clinical practice guideline for diagnosis, treatment and follow-up. Ann Oncol Off J Eur Soc Med Oncol. 2024;35:159–82. 10.1016/j.annonc.2023.11.016 38101773

[42] Davey MG, Browne F, Miller N, Lowery AJ, Kerin MJ. Pathological complete response as a surrogate to improved survival in human epidermal growth factor receptor-2-positive breast cancer: systematic review and meta-analysis. BJS Open. 2022;6:zrac028. 35512244 10.1093/bjsopen/zrac028 PMC9071230

[43] Cortazar P, et al. Pathological complete response and long-term clinical benefit in breast cancer: the CTNeoBC pooled analysis. Lancet. 2014;384:164–72. 24529560 10.1016/S0140-6736(13)62422-8

[44] Rodríguez IJ, Bernal-Estévez DA, Llano-León M, Bonilla CE. Parra-López, C. A. Neoadjuvant chemotherapy modulates exhaustion of T cells in breast cancer patients. PLoS ONE. 2023;18:e0280851. 36763585 10.1371/journal.pone.0280851 PMC9916600

[45] Kresovich JK, et al. Circulating leukocyte subsets before and after a breast cancer diagnosis and therapy. JAMA Netw Open. 2024;7:e2356113. 38358741 10.1001/jamanetworkopen.2023.56113 PMC10870180

[46] Lu Y, Zhang Q, Wang J, Zhang L. Characteristics and postoperative dynamic changes in Circulating CD4 + helper T lymphocytes in patients with breast cancer. Front Oncol 13, (2023). 10.3389/fonc.2023.1118346 PMC10011473 36925914

[47] Sánchez-Margalet V, et al. Circulating regulatory T cells from breast cancer patients in response to neoadjuvant chemotherapy. Transl Cancer Res. 2019;8:59–65. 35116734 10.21037/tcr.2018.12.30 PMC8798280

[48] Cattin S, et al. Circulating immune cell populations related to primary breast cancer, surgical removal, and radiotherapy revealed by flow cytometry analysis. Breast Cancer Res BCR. 2021;23:64. 34090509 10.1186/s13058-021-01441-8 PMC8180078

[49] Gruber I, et al. Relationship between Circulating tumor cells and peripheral T-Cells in patients with primary breast cancer. Anticancer Res. 2013;33:2233–8. 23645781

[50] Speiser DE, Ho P-C, Verdeil G. Regulatory circuits of T cell function in cancer. Nat Rev Immunol. 2016;16:599–611. 27526640 10.1038/nri.2016.80

[51] Velardi E, Tsai JJ, van den Brink M. R. M. T cell regeneration after immunological injury. Nat Rev Immunol. 2021;21:277–91. 33097917 10.1038/s41577-020-00457-z PMC7583557

[52] Choyke PL, et al. Thymic atrophy and regrowth in response to chemotherapy: CT evaluation. AJR Am J Roentgenol. 1987;149:269–72. 3496749 10.2214/ajr.149.2.269

[53] Qiu L, et al. Thymic rebound hyperplasia post-chemotherapy mistaken as disease progression in a patient with lymphoma involving mediastinum: a case report and reflection. BMC Surg. 2021;21:38. 33446156 10.1186/s12893-021-01048-y PMC7809830

[54] Sun D-P, et al. Thymic hyperplasia after chemotherapy in adults with mature B cell lymphoma and its influence on thymic output and CD4 + T cells repopulation. Oncoimmunology. 2016;5:e1137417. 27467956 10.1080/2162402X.2015.1137417 PMC4910735

[55] Das RK, O’Connor RS, Grupp SA, Barrett DM. Lingering effects of chemotherapy on mature T cells impair proliferation. Blood Adv. 2020;4:4653–64. 33002133 10.1182/bloodadvances.2020001797 PMC7556159

[56] van den Broek T, Borghans JAM, van Wijk F. The full spectrum of human Naive T cells. Nat Rev Immunol. 2018;18:363–73. 29520044 10.1038/s41577-018-0001-y

[57] Mackall CL, et al. Age, thymopoiesis, and CD4 + T-Lymphocyte regeneration after intensive chemotherapy. N Engl J Med. 1995;332:143–9. 7800006 10.1056/NEJM199501193320303

[58] Chen D, Tang T-X, Deng H, Yang X-P, Tang Z-H. Interleukin-7 biology and its effects on immune cells: mediator of generation, differentiation, survival, and homeostasis. Front Immunol. 2021;12:747324. 34925323 10.3389/fimmu.2021.747324 PMC8674869

[59] Azevedo RI, et al. IL-7 sustains CD31 expression in human Naive CD4 + T cells and preferentially expands the CD31 + subset in a PI3K-dependent manner. Blood. 2009;113:2999–3007. 19008454 10.1182/blood-2008-07-166223

[60] Winer H, et al. IL-7: comprehensive review. Cytokine. 2022;160:156049. 36201890 10.1016/j.cyto.2022.156049

[61] Rochman Y, Spolski R, Leonard WJ. New insights into the regulation of T cells by Γc family cytokines. Nat Rev Immunol. 2009;9:480–90. 19543225 10.1038/nri2580 PMC2814538

[62] Devi M, Vijayalakshmi D, Dhivya K, Janane M, Memory T. Cells (CD45RO) role and evaluation in pathogenesis of lichen planus and lichenoid mucositis. J Clin Diagn Res JCDR. 2017;11:ZC84–6. 28658915 10.7860/JCDR/2017/26866.9930 PMC5483817

[63] Jochems C, Schlom J. Tumor-infiltrating immune cells and prognosis: the potential link between conventional cancer therapy and immunity. Exp Biol Med Maywood NJ. 2011;236:567–79. 10.1258/ebm.2011.011007 PMC3229261 21486861

[64] Pagès F, et al. Effector memory T cells, early metastasis, and survival in colorectal cancer. N Engl J Med. 2005;353:2654–66. 16371631 10.1056/NEJMoa051424

[65] Rudqvist N-P, et al. Immunotherapy targeting different immune compartments in combination with radiation therapy induces regression of resistant tumors. Nat Commun. 2023;14:5146. 37620372 10.1038/s41467-023-40844-3 PMC10449830

[66] Sasatomi T, Oochi T, Ogata Y, Akagi Y, Shirouzu K. CTLs/regulatory T-cells ratio as a prediction marker of chemotherapy in metastatic colorectal cancer. J Clin Oncol. 2013;31:e14684–14684.

[67] Xu T, Lu J, An H. The relative change in regulatory T cells / T helper lymphocytes ratio as parameter for prediction of therapy efficacy in metastatic colorectal cancer patients. Oncotarget. 2017;8:109079. 29312592 10.18632/oncotarget.22606 PMC5752505

[68] Liu F, et al. CD8^+^ cytotoxic T cell and FOXP3^+^ regulatory T cell infiltration in relation to breast cancer survival and molecular subtypes. Breast Cancer Res Treat. 2011;130:645–55. 21717105 10.1007/s10549-011-1647-3

[69] Goda N, et al. The ratio of CD8 + lymphocytes to tumor-infiltrating suppressive FOXP3 + effector regulatory T cells is associated with treatment response in invasive breast cancer. Discov Oncol. 2022;13:27. 35438346 10.1007/s12672-022-00482-5 PMC9018954

[70] Baras AS, et al. The ratio of CD8 to Treg tumor-infiltrating lymphocytes is associated with response to cisplatin-based neoadjuvant chemotherapy in patients with muscle invasive urothelial carcinoma of the bladder. OncoImmunology. 2016;5:e1134412. 27467953 10.1080/2162402X.2015.1134412 PMC4910705

[71] Duan S, Thomas PG. Balancing immune protection and immune pathology by CD8(+) T-Cell responses to influenza infection. Front Immunol. 2016;7:25. 26904022 10.3389/fimmu.2016.00025 PMC4742794

[72] Briukhovetska D, et al. Interleukins in cancer: from biology to therapy. Nat Rev Cancer. 2021;21:481–99. 34083781 10.1038/s41568-021-00363-z PMC8173513

[73] Xiao Y, Huang Y, Jiang J, Chen Y, Wei C. Identification of the prognostic value of Th1/Th2 ratio and a novel prognostic signature in basal-like breast cancer. Hereditas. 2023;160:2. 36694223 10.1186/s41065-023-00265-0 PMC9875389

[74] Nagata K, et al. Selective expression of a novel surface molecule by human Th2 cells in vivo. J Immunol. 1999;162:1278–86. 9973380

[75] Rudulier CD, Tonti E, James E, Kwok WW, Larché M. Modulation of CRTh2 expression on allergen-specific T cells following peptide immunotherapy. Allergy. 2019;74:2157–66. 31077596 10.1111/all.13867 PMC6817377

[76] Mousset CM, et al. Comprehensive phenotyping of T cells using flow cytometry. Cytometry A. 2019;95:647–54. 30714682 10.1002/cyto.a.23724

[77] Snyder JD et al. Protein kinase D1 in myeloid lineage cells contributes to the accumulation of CXCR3 + CCR6 + nonconventional Th1 cells in the lungs and potentiates hypersensitivity pneumonitis caused by S. rectivirgula. Front Immunol 15, (2024). 10.3389/fimmu.2024.1403155 PMC11502317 39464896

[78] Acosta-Rodriguez EV, et al. Surface phenotype and antigenic specificity of human Interleukin 17–producing T helper memory cells. Nat Immunol. 2007;8:639–46. 17486092 10.1038/ni1467

[79] Becattini S, et al. Functional heterogeneity of human memory CD4 + T cell clones primed by pathogens or vaccines. Science. 2015;347:400–6. 25477212 10.1126/science.1260668

[80] Wang R, Jaw JJ, Stutzman NC, Zou Z, Sun PD. Natural killer cell-produced IFN-γ and TNF-α induce target cell cytolysis through up-regulation of ICAM-1. J Leukoc Biol. 2012;91:299–309. 22045868 10.1189/jlb.0611308 PMC3290424

[81] Wang Z et al. IL-10 enhances human natural killer cell effector functions via metabolic reprogramming regulated by mTORC1 signaling. Front Immunol 12, (2021). 10.3389/fimmu.2021.619195 PMC7940510 33708210

[82] Cruceriu D, Baldasici O, Balacescu O, Berindan-Neagoe I. The dual role of tumor necrosis factor-alpha (TNF-α) in breast cancer: molecular insights and therapeutic approaches. Cell Oncol. 2020;43:1–18. 10.1007/s13402-019-00489-1 PMC12990688 31900901

[83] Ruffell B, Coussens LM. Macrophages and therapeutic resistance in cancer. Cancer Cell. 2015;27:462–72. 25858805 10.1016/j.ccell.2015.02.015 PMC4400235

