# Supplementary material for: Dynamics of T cell subpopulations and plasma cytokines during the first year of antineoplastic therapy in patients with breast cancer: the BEGYN-1 study
Source: Breast Cancer Res. 2025 Apr 1;27:50. doi: 10.1186/s13058-025-01997-9 (PMC11963634; doi:10.1186/s13058-025-01997-9)
Supplement: Supplementary file 1 — Supplementary Material 1 [file 13058_2025_1997_MOESM1_ESM.docx]

**Table S1.** Flow cytometry antibodies

| **marker** | **fluorophore** | **clone** | **vendor** | **catalog** | **RRID** |
| --- | --- | --- | --- | --- | --- |
|  |  |  |  |  |  |
| CD127 | Alexa-Fluor 647 | HIL-7R-M21 | BD | 558598 | AB_647113 |
| CD183 | BV480 | CXCR3 | BD | 746283 | AB_2743613 |
| CD194 | PE | 1G1 | BD | 551120 | AB_394054 |
| CD196 | APC-R700 | CCR6 | BD | 565173 | AB_2739092 |
| CD25 | BV421 | M-A251 | BD | 562442 | AB_11154578 |
| CD294 | BV650 | BM16 | BD | 740616 | AB_2740314 |
| CD3 | BV786 | SK7 | BD | 563800 | AB_2744384 |
| CD31 | BV421 | L133.1 | BD | 744801 | AB_2742490 |
| CD4 | PE-CF594 | SK3 | BD | 566317 | AB_2739679 |
| CD45RO | BV605 | UCHL1 | BD | 562791 | AB_2744411 |
| CD62L | BB700 | SK11 | BD | 745995 | AB_2743396 |
| CD8 | APC-R700 | SK1 | BD | 565192 | AB_2739104 |

**Table S2.** Descriptive statistics of flow cytometry data (treatment)

| cell  population | group | time of  assessment  [months] | minimum | Q1 | median | Q3 | maximum | n |
| --- | --- | --- | --- | --- | --- | --- | --- | --- |
| T cells CD3+ relative amount of viable cells [%] | all | 0 | 3.55 | 32.10 | 44.60 | 56.70 | 70.80 | 76 |
|  |  | 3 | 5.45 | 33.60 | 40.30 | 56.40 | 75.90 | 72 |
|  |  | 6 | 1.90 | 27.50 | 43.30 | 52.80 | 76.90 | 80 |
|  |  | 9 | 7.90 | 30.20 | 42.10 | 54.65 | 78.20 | 83 |
|  |  | 12 | 5.68 | 24.95 | 34.35 | 49.28 | 80.90 | 74 |
|  | CHT | 0 | 18.50 | 36.30 | 46.40 | 56.80 | 70.10 | 48 |
|  |  | 3 | 5.45 | 34.80 | 40.70 | 60.70 | 75.90 | 44 |
|  |  | 6 | 1.90 | 29.25 | 44.20 | 53.03 | 76.90 | 43 |
|  |  | 9 | 15.00 | 33.40 | 45.40 | 56.25 | 67.80 | 46 |
|  |  | 12 | 10.20 | 24.95 | 37.10 | 52.43 | 65.70 | 42 |
|  | NCHT | 0 | 3.55 | 30.55 | 42.20 | 52.48 | 70.80 | 28 |
|  |  | 3 | 6.98 | 25.63 | 39.15 | 53.08 | 75.90 | 28 |
|  |  | 6 | 4.96 | 27.10 | 39.60 | 52.70 | 76.30 | 37 |
|  |  | 9 | 7.90 | 28.20 | 37.20 | 48.45 | 78.20 | 37 |
|  |  | 12 | 5.68 | 23.25 | 33.30 | 46.08 | 80.90 | 32 |
| Th  CD3+CD4+ relative amount of CD3+ [%] | all | 0 | 8.72 | 51.10 | 62.60 | 73.60 | 92.30 | 76 |
|  |  | 3 | 20.70 | 44.60 | 56.00 | 70.10 | 90.40 | 72 |
|  |  | 6 | 25.70 | 45.20 | 60.40 | 68.80 | 92.10 | 80 |
|  |  | 9 | 14.60 | 47.53 | 57.10 | 67.50 | 90.90 | 83 |
|  |  | 12 | 15.30 | 44.10 | 55.25 | 65.68 | 89.40 | 74 |
|  | CHT | 0 | 38.90 | 50.50 | 61.20 | 71.20 | 85.00 | 48 |
|  |  | 3 | 20.70 | 44.60 | 53.60 | 63.60 | 83.90 | 44 |
|  |  | 6 | 25.70 | 42.53 | 56.15 | 63.85 | 87.60 | 43 |
|  |  | 9 | 14.60 | 45.15 | 53.10 | 64.35 | 85.40 | 46 |
|  |  | 12 | 15.30 | 38.50 | 53.55 | 63.63 | 79.70 | 42 |
|  | NCHT | 0 | 8.72 | 52.55 | 66.70 | 75.78 | 92.30 | 28 |
|  |  | 3 | 31.00 | 45.75 | 69.15 | 75.60 | 90.40 | 28 |
|  |  | 6 | 30.60 | 48.30 | 64.90 | 72.00 | 92.10 | 37 |
|  |  | 9 | 34.70 | 50.20 | 63.60 | 70.55 | 90.90 | 37 |
|  |  | 12 | 25.20 | 49.90 | 63.50 | 68.15 | 89.40 | 32 |
| CTL CD3+CD8+ relative amount of CD3+ [%] | all | 0 | 0.01 | 16.40 | 22.80 | 31.10 | 53.10 | 76 |
|  |  | 3 | 0.02 | 18.50 | 23.40 | 32.20 | 60.60 | 72 |
|  |  | 6 | 0.01 | 17.50 | 23.50 | 34.30 | 51.80 | 80 |
|  |  | 9 | 0.01 | 18.00 | 25.70 | 32.58 | 78.40 | 83 |
|  |  | 12 | 0.01 | 18.98 | 28.60 | 37.28 | 69.30 | 74 |
|  | CHT | 0 | 3.72 | 15.50 | 24.50 | 30.60 | 52.80 | 48 |
|  |  | 3 | 5.60 | 20.00 | 24.90 | 37.60 | 60.60 | 44 |
|  |  | 6 | 0.65 | 20.65 | 27.10 | 35.18 | 51.80 | 43 |
|  |  | 9 | 9.07 | 20.25 | 27.90 | 38.60 | 78.40 | 46 |
|  |  | 12 | 6.10 | 23.53 | 30.10 | 39.88 | 69.30 | 42 |
|  | NCHT | 0 | 0.01 | 16.65 | 19.80 | 31.10 | 53.10 | 28 |
|  |  | 3 | 0.02 | 13.45 | 19.20 | 28.63 | 50.00 | 28 |
|  |  | 6 | 0.01 | 15.20 | 21.70 | 33.55 | 41.70 | 37 |
|  |  | 9 | 0.01 | 14.65 | 20.20 | 30.15 | 46.20 | 37 |
|  |  | 12 | 0.01 | 16.65 | 21.85 | 33.25 | 52.10 | 32 |

**Table S2 (continued).** Descriptive statistics of flow cytometry data (treatment)

| cell population | group | time of assessment  [months] | minimum | Q1 | median | Q3 | maximum | n |
| --- | --- | --- | --- | --- | --- | --- | --- | --- |
| naive effector Th CD3+CD4+CD45RO-CD62L- relative amount of CD3+CD4+ [%] | all | 0 | 5.26 | 19.80 | 27.00 | 37.80 | 88.10 | 76 |
|  |  | 3 | 2.88 | 12.30 | 20.90 | 33.80 | 61.60 | 72 |
|  |  | 6 | 2.25 | 11.50 | 17.90 | 27.40 | 61.60 | 80 |
|  |  | 9 | 1.05 | 8.37 | 15.45 | 22.83 | 62.40 | 83 |
|  |  | 12 | 1.40 | 8.78 | 15.15 | 23.33 | 55.60 | 74 |
|  | CHT | 0 | 5.26 | 19.50 | 27.00 | 37.00 | 60.10 | 48 |
|  |  | 3 | 2.88 | 10.40 | 21.40 | 35.00 | 51.50 | 44 |
|  |  | 6 | 2.25 | 9.01 | 16.35 | 27.10 | 61.60 | 43 |
|  |  | 9 | 1.05 | 5.56 | 14.80 | 20.15 | 56.10 | 46 |
|  |  | 12 | 1.40 | 7.45 | 12.30 | 23.33 | 39.30 | 42 |
|  | NCHT | 0 | 8.46 | 20.70 | 28.10 | 41.55 | 88.10 | 28 |
|  |  | 3 | 8.86 | 14.25 | 20.60 | 27.90 | 61.60 | 28 |
|  |  | 6 | 6.36 | 15.70 | 18.70 | 28.10 | 58.00 | 37 |
|  |  | 9 | 3.37 | 11.85 | 15.90 | 26.30 | 62.40 | 37 |
|  |  | 12 | 6.95 | 10.10 | 16.80 | 23.53 | 55.60 | 32 |
| memory effector Th CD3+CD4+CD45RO+CD62L- relative amount of CD3+CD4+ [%] | all | 0 | 6.30 | 26.70 | 38.50 | 49.50 | 74.40 | 76 |
|  |  | 3 | 5.61 | 33.80 | 43.10 | 53.00 | 74.80 | 72 |
|  |  | 6 | 3.44 | 38.40 | 51.50 | 61.30 | 77.80 | 80 |
|  |  | 9 | 20.10 | 40.25 | 52.30 | 60.78 | 88.40 | 83 |
|  |  | 12 | 19.00 | 35.53 | 50.80 | 61.05 | 83.50 | 74 |
|  | CHT | 0 | 6.30 | 25.90 | 37.40 | 50.50 | 74.40 | 48 |
|  |  | 3 | 5.61 | 34.20 | 43.10 | 52.50 | 74.80 | 44 |
|  |  | 6 | 3.44 | 38.08 | 52.30 | 62.23 | 77.80 | 43 |
|  |  | 9 | 29.40 | 45.30 | 54.00 | 67.10 | 81.60 | 46 |
|  |  | 12 | 26.20 | 37.75 | 51.45 | 60.20 | 83.50 | 42 |
|  | NCHT | 0 | 7.69 | 28.20 | 39.90 | 47.78 | 64.30 | 28 |
|  |  | 3 | 16.00 | 33.10 | 45.45 | 53.20 | 68.80 | 28 |
|  |  | 6 | 18.20 | 38.70 | 49.40 | 59.25 | 70.10 | 37 |
|  |  | 9 | 20.10 | 38.20 | 47.50 | 57.10 | 88.40 | 37 |
|  |  | 12 | 19.00 | 32.55 | 46.85 | 61.55 | 78.30 | 32 |
| naive central Th CD3+CD4+CD45RO-CD62L+ relative amount of CD3+CD4+ [%] | all | 0 | 1.04 | 4.97 | 12.40 | 21.20 | 48.90 | 76 |
|  |  | 3 | 0.72 | 4.27 | 8.82 | 14.60 | 38.80 | 72 |
|  |  | 6 | 0.33 | 3.12 | 5.63 | 13.10 | 45.50 | 80 |
|  |  | 9 | 0.29 | 2.02 | 5.90 | 11.43 | 30.10 | 83 |
|  |  | 12 | 0.47 | 2.62 | 6.42 | 14.93 | 38.20 | 74 |
|  | CHT | 0 | 1.14 | 6.71 | 12.20 | 21.90 | 45.40 | 48 |
|  |  | 3 | 0.72 | 4.08 | 9.86 | 13.70 | 35.40 | 44 |
|  |  | 6 | 0.33 | 2.30 | 4.35 | 10.75 | 45.50 | 43 |
|  |  | 9 | 0.29 | 1.62 | 5.18 | 8.87 | 26.70 | 46 |
|  |  | 12 | 0.47 | 2.20 | 5.84 | 12.33 | 34.70 | 42 |
|  | NCHT | 0 | 1.04 | 4.06 | 13.00 | 21.00 | 48.90 | 28 |
|  |  | 3 | 0.90 | 4.84 | 7.98 | 17.68 | 38.80 | 28 |
|  |  | 6 | 1.28 | 3.52 | 6.19 | 14.45 | 34.20 | 37 |
|  |  | 9 | 0.51 | 4.05 | 8.83 | 15.85 | 30.10 | 37 |
|  |  | 12 | 0.75 | 2.93 | 7.13 | 18.08 | 38.20 | 32 |

**Table S2 (continued).** Descriptive statistics of flow cytometry data (treatment)

| cell population | group | time of assessment  [months] | minimum | Q1 | median | Q3 | maximum | n |
| --- | --- | --- | --- | --- | --- | --- | --- | --- |
| memory central Th CD3+CD4+CD45RO+CD62L+ relative amount of CD3+CD4+ [%] | all | 0 | 0.77 | 9.08 | 14.00 | 20.40 | 58.70 | 76 |
|  |  | 3 | 1.99 | 13.50 | 21.00 | 28.00 | 58.60 | 72 |
|  |  | 6 | 2.71 | 14.40 | 18.90 | 27.50 | 62.10 | 80 |
|  |  | 9 | 0.46 | 16.60 | 22.35 | 31.78 | 63.50 | 83 |
|  |  | 12 | 7.81 | 15.73 | 22.95 | 34.65 | 58.60 | 74 |
|  | CHT | 0 | 3.48 | 9.53 | 14.30 | 20.40 | 58.70 | 48 |
|  |  | 3 | 3.02 | 13.10 | 19.20 | 33.80 | 58.60 | 44 |
|  |  | 6 | 2.78 | 15.35 | 20.90 | 28.68 | 62.10 | 43 |
|  |  | 9 | 2.49 | 14.50 | 22.20 | 34.85 | 63.50 | 46 |
|  |  | 12 | 7.81 | 15.38 | 23.70 | 34.88 | 58.60 | 42 |
|  | NCHT | 0 | 0.77 | 8.61 | 13.65 | 20.63 | 42.10 | 28 |
|  |  | 3 | 1.99 | 17.65 | 21.15 | 25.70 | 39.70 | 28 |
|  |  | 6 | 2.71 | 13.30 | 18.30 | 25.65 | 35.50 | 37 |
|  |  | 9 | 0.46 | 16.85 | 22.80 | 30.50 | 39.60 | 37 |
|  |  | 12 | 8.74 | 16.20 | 22.60 | 34.08 | 48.40 | 32 |
| naive effector CTL CD3+CD8+CD45RO-CD62L- relative amount of CD3+CD4+ [%] | all | 0 | 18.00 | 34.40 | 45.60 | 57.80 | 100.00 | 76 |
|  |  | 3 | 18.80 | 29.60 | 43.60 | 60.60 | 79.40 | 72 |
|  |  | 6 | 17.70 | 31.50 | 42.80 | 54.60 | 74.20 | 80 |
|  |  | 9 | 15.00 | 28.35 | 40.40 | 52.48 | 100.00 | 83 |
|  |  | 12 | 13.00 | 29.43 | 41.80 | 53.58 | 86.10 | 74 |
|  | CHT | 0 | 23.40 | 35.30 | 46.90 | 57.80 | 76.40 | 48 |
|  |  | 3 | 22.50 | 29.70 | 45.10 | 63.90 | 79.40 | 44 |
|  |  | 6 | 20.10 | 33.78 | 42.80 | 55.00 | 74.20 | 43 |
|  |  | 9 | 15.50 | 28.55 | 37.10 | 52.65 | 82.20 | 46 |
|  |  | 12 | 19.80 | 30.68 | 41.80 | 54.48 | 86.10 | 42 |
|  | NCHT | 0 | 18.00 | 33.88 | 45.45 | 65.03 | 100.00 | 28 |
|  |  | 3 | 18.80 | 29.53 | 41.30 | 50.88 | 67.30 | 28 |
|  |  | 6 | 17.70 | 30.55 | 42.80 | 53.60 | 67.10 | 37 |
|  |  | 9 | 15.00 | 27.75 | 42.00 | 52.25 | 100.00 | 37 |
|  |  | 12 | 13.00 | 26.58 | 41.40 | 50.25 | 71.40 | 32 |
| memory effector CTL CD3+CD8+CD45RO+CD62L- relative amount of CD3+CD8+ [%] | all | 0 | 0.00 | 22.60 | 33.70 | 43.50 | 67.10 | 76 |
|  |  | 3 | 13.50 | 24.20 | 31.20 | 42.90 | 66.60 | 72 |
|  |  | 6 | 13.10 | 28.00 | 36.50 | 46.00 | 70.50 | 80 |
|  |  | 9 | 0.00 | 27.60 | 38.10 | 50.18 | 69.30 | 83 |
|  |  | 12 | 11.00 | 27.55 | 39.45 | 49.68 | 69.60 | 74 |
|  | CHT | 0 | 10.40 | 24.10 | 33.20 | 39.50 | 67.10 | 48 |
|  |  | 3 | 13.50 | 19.00 | 27.30 | 41.40 | 64.30 | 44 |
|  |  | 6 | 13.10 | 24.00 | 32.85 | 44.58 | 70.50 | 43 |
|  |  | 9 | 10.20 | 25.20 | 38.10 | 51.95 | 69.30 | 46 |
|  |  | 12 | 11.00 | 25.88 | 38.40 | 50.83 | 69.60 | 42 |
|  | NCHT | 0 | 0.00 | 20.63 | 35.95 | 47.13 | 65.30 | 28 |
|  |  | 3 | 14.20 | 30.90 | 38.60 | 47.00 | 66.60 | 28 |
|  |  | 6 | 15.00 | 30.35 | 39.70 | 50.40 | 62.60 | 37 |
|  |  | 9 | 0.00 | 30.55 | 39.00 | 48.50 | 62.40 | 37 |
|  |  | 12 | 16.80 | 31.03 | 40.60 | 48.73 | 65.00 | 32 |

**Table S2 (continued).** Descriptive statistics of flow cytometry data (treatment)

| cell population | group | time of assessment  [months] | minimum | Q1 | median | Q3 | maximum | n |
| --- | --- | --- | --- | --- | --- | --- | --- | --- |
| naive central CTL CD3+CD8+CD45RO-CD62L+ relative amount of CD3+CD8+ [%] | all | 0 | 0.00 | 4.21 | 11.30 | 19.10 | 55.40 | 76 |
|  |  | 3 | 0.00 | 4.37 | 10.60 | 19.80 | 56.60 | 72 |
|  |  | 6 | 0.00 | 3.68 | 8.75 | 16.80 | 53.40 | 80 |
|  |  | 9 | 0.00 | 3.84 | 9.85 | 15.55 | 36.50 | 83 |
|  |  | 12 | 0.00 | 3.20 | 8.55 | 15.80 | 45.00 | 74 |
|  | CHT | 0 | 0.00 | 5.18 | 12.50 | 19.40 | 55.40 | 48 |
|  |  | 3 | 1.20 | 5.14 | 11.10 | 20.60 | 56.60 | 44 |
|  |  | 6 | 0.00 | 3.68 | 9.31 | 19.83 | 53.40 | 43 |
|  |  | 9 | 0.00 | 3.36 | 9.53 | 19.15 | 36.50 | 46 |
|  |  | 12 | 0.00 | 3.18 | 7.55 | 13.85 | 36.60 | 42 |
|  | NCHT | 0 | 0.00 | 3.04 | 7.84 | 15.65 | 41.10 | 28 |
|  |  | 3 | 0.00 | 3.53 | 9.75 | 17.00 | 33.10 | 28 |
|  |  | 6 | 0.00 | 3.55 | 8.49 | 16.45 | 42.00 | 37 |
|  |  | 9 | 0.00 | 4.26 | 9.92 | 15.25 | 30.10 | 37 |
|  |  | 12 | 0.00 | 3.22 | 9.45 | 17.88 | 45.00 | 32 |
| memory central CTL CD3+CD8+CD45RO+CD62L+ relative amount of CD3+CD8+ [%] | all | 0 | 0.00 | 2.81 | 4.30 | 7.30 | 20.20 | 76 |
|  |  | 3 | 0.00 | 3.21 | 5.06 | 9.99 | 20.90 | 72 |
|  |  | 6 | 0.00 | 3.85 | 6.06 | 9.76 | 22.60 | 80 |
|  |  | 9 | 0.00 | 3.57 | 7.29 | 12.00 | 25.70 | 83 |
|  |  | 12 | 0.00 | 4.43 | 6.75 | 11.73 | 21.30 | 74 |
|  | CHT | 0 | 0.00 | 2.81 | 4.30 | 5.98 | 20.20 | 48 |
|  |  | 3 | 0.68 | 2.22 | 4.51 | 10.00 | 20.90 | 44 |
|  |  | 6 | 1.01 | 3.39 | 4.75 | 9.10 | 21.70 | 43 |
|  |  | 9 | 0.00 | 3.13 | 5.29 | 9.60 | 25.70 | 46 |
|  |  | 12 | 0.91 | 3.42 | 5.96 | 8.40 | 21.10 | 42 |
|  | NCHT | 0 | 0.00 | 2.42 | 4.46 | 8.58 | 17.30 | 28 |
|  |  | 3 | 0.00 | 4.34 | 6.47 | 9.97 | 18.80 | 28 |
|  |  | 6 | 0.00 | 4.51 | 6.47 | 10.25 | 22.60 | 37 |
|  |  | 9 | 0.00 | 4.57 | 8.13 | 13.90 | 21.90 | 37 |
|  |  | 12 | 0.00 | 4.58 | 8.02 | 12.85 | 21.30 | 32 |
| thymus negative Th CD3+CD4+CD45RO-CD31- relative amount of CD3+CD4+ [%] | all | 0 | 0.18 | 9.41 | 14.70 | 20.00 | 39.30 | 76 |
|  |  | 3 | 1.33 | 6.71 | 11.60 | 16.60 | 36.30 | 72 |
|  |  | 6 | 1.18 | 5.36 | 10.50 | 14.60 | 38.30 | 80 |
|  |  | 9 | 0.39 | 3.73 | 8.24 | 13.38 | 40.30 | 83 |
|  |  | 12 | 0.85 | 4.02 | 8.35 | 13.83 | 34.90 | 74 |
|  | CHT | 0 | 2.83 | 9.85 | 13.90 | 19.40 | 39.30 | 48 |
|  |  | 3 | 1.33 | 6.90 | 10.60 | 15.50 | 36.30 | 44 |
|  |  | 6 | 1.38 | 5.29 | 8.15 | 13.05 | 24.80 | 43 |
|  |  | 9 | 0.43 | 2.81 | 7.05 | 10.10 | 21.50 | 46 |
|  |  | 12 | 0.85 | 3.71 | 7.59 | 12.58 | 23.50 | 42 |
|  | NCHT | 0 | 0.18 | 7.25 | 16.00 | 24.53 | 36.20 | 28 |
|  |  | 3 | 3.08 | 4.94 | 12.10 | 19.38 | 35.30 | 28 |
|  |  | 6 | 1.18 | 6.37 | 11.40 | 15.45 | 38.30 | 37 |
|  |  | 9 | 0.39 | 5.66 | 11.50 | 14.70 | 40.30 | 37 |
|  |  | 12 | 1.51 | 4.52 | 10.44 | 14.18 | 34.90 | 32 |

**Table S2 (continued).** Descriptive statistics of flow cytometry data (treatment)

| cell population | group | time of assessment  [months] | minimum | Q1 | median | Q3 | maximum | n |
| --- | --- | --- | --- | --- | --- | --- | --- | --- |
| thymus positive Th CD3+CD4+CD45RO-CD31+ relative amount of CD3+CD4+ [%] | all | 0 | 5.23 | 18.30 | 25.10 | 38.90 | 89.20 | 76 |
|  |  | 3 | 2.42 | 13.00 | 18.10 | 28.60 | 50.40 | 72 |
|  |  | 6 | 1.70 | 9.18 | 16.40 | 24.30 | 49.40 | 80 |
|  |  | 9 | 1.16 | 7.34 | 13.90 | 19.28 | 39.60 | 83 |
|  |  | 12 | 1.26 | 8.31 | 13.50 | 21.15 | 39.30 | 74 |
|  | CHT | 0 | 5.23 | 18.20 | 26.50 | 41.50 | 61.40 | 48 |
|  |  | 3 | 2.42 | 13.00 | 18.10 | 27.90 | 46.40 | 44 |
|  |  | 6 | 1.70 | 8.37 | 14.40 | 23.33 | 49.40 | 43 |
|  |  | 9 | 1.16 | 5.16 | 12.10 | 18.10 | 39.60 | 46 |
|  |  | 12 | 1.26 | 7.73 | 12.80 | 19.33 | 37.40 | 42 |
|  | NCHT | 0 | 6.77 | 19.03 | 24.05 | 36.65 | 89.20 | 28 |
|  |  | 3 | 6.85 | 12.18 | 18.25 | 30.28 | 50.40 | 28 |
|  |  | 6 | 5.11 | 9.33 | 19.10 | 26.15 | 44.10 | 37 |
|  |  | 9 | 3.94 | 10.80 | 16.60 | 24.50 | 35.60 | 37 |
|  |  | 12 | 3.64 | 8.76 | 15.65 | 24.50 | 39.30 | 32 |
| Th1 CD3+CD4+CD183+CD196+ relative amount of CD3+CD4+ [%] | all | 0 | 0.14 | 0.62 | 1.67 | 3.20 | 7.12 | 74 |
|  |  | 3 | 0.00 | 0.54 | 1.64 | 2.83 | 7.31 | 72 |
|  |  | 6 | 0.00 | 0.79 | 2.15 | 3.45 | 10.30 | 80 |
|  |  | 9 | 0.10 | 0.84 | 2.00 | 3.18 | 21.00 | 81 |
|  |  | 12 | 0.02 | 0.66 | 1.62 | 3.35 | 12.80 | 75 |
|  | CHT | 0 | 0.14 | 0.61 | 1.67 | 3.20 | 7.06 | 46 |
|  |  | 3 | 0.00 | 0.47 | 1.37 | 2.38 | 7.18 | 44 |
|  |  | 6 | 0.05 | 0.53 | 2.16 | 3.47 | 6.07 | 43 |
|  |  | 9 | 0.10 | 0.62 | 2.05 | 3.18 | 7.53 | 45 |
|  |  | 12 | 0.02 | 0.35 | 1.38 | 3.10 | 7.33 | 43 |
|  | NCHT | 0 | 0.15 | 0.96 | 1.69 | 3.79 | 7.12 | 28 |
|  |  | 3 | 0.14 | 0.61 | 1.92 | 3.99 | 7.31 | 28 |
|  |  | 6 | 0.09 | 1.28 | 2.14 | 3.58 | 10.30 | 37 |
|  |  | 9 | 0.16 | 0.97 | 1.99 | 3.39 | 21.00 | 36 |
|  |  | 12 | 0.10 | 0.75 | 1.82 | 3.43 | 12.80 | 32 |
| naive Th1 CD3+CD4+CD183+CD196+CD45RO- relative amount of CD3+CD4+CD183+CD196+ [%] | all | 0 | 10.90 | 28.08 | 39.35 | 48.33 | 80.00 | 74 |
|  |  | 3 | 0.00 | 24.83 | 35.90 | 46.28 | 81.20 | 72 |
|  |  | 6 | 0.00 | 23.33 | 33.40 | 40.88 | 78.80 | 80 |
|  |  | 9 | 7.02 | 23.55 | 30.90 | 39.55 | 79.10 | 81 |
|  |  | 12 | 8.33 | 24.10 | 28.90 | 40.00 | 100.00 | 75 |
|  | CHT | 0 | 10.90 | 26.10 | 35.35 | 46.70 | 68.40 | 46 |
|  |  | 3 | 0.00 | 21.98 | 37.45 | 48.88 | 66.00 | 44 |
|  |  | 6 | 8.51 | 23.10 | 32.20 | 38.40 | 69.00 | 43 |
|  |  | 9 | 7.02 | 22.40 | 29.40 | 34.80 | 68.30 | 45 |
|  |  | 12 | 11.00 | 24.10 | 29.20 | 40.00 | 100.00 | 43 |
|  | NCHT | 0 | 20.70 | 34.53 | 43.85 | 52.80 | 80.00 | 28 |
|  |  | 3 | 19.40 | 30.25 | 35.70 | 42.30 | 81.20 | 28 |
|  |  | 6 | 13.10 | 24.95 | 34.40 | 43.25 | 78.80 | 37 |
|  |  | 9 | 11.90 | 24.83 | 33.05 | 42.05 | 79.10 | 36 |
|  |  | 12 | 8.33 | 24.48 | 28.70 | 39.78 | 71.70 | 32 |

**Table S2 (continued).** Descriptive statistics of flow cytometry data (treatment)

| cell population | group | time of assessment  [months] | minimum | Q1 | median | Q3 | maximum | n |
| --- | --- | --- | --- | --- | --- | --- | --- | --- |
| memory Th1 CD3+CD4+CD183+CD196+CD45RO+ relative amount of CD3+CD4+CD183+CD196+ [%] | all | 0 | 20.00 | 51.68 | 60.65 | 71.93 | 89.10 | 74 |
|  |  | 3 | 0.00 | 51.95 | 64.05 | 74.65 | 94.00 | 72 |
|  |  | 6 | 0.00 | 58.88 | 66.35 | 75.88 | 91.50 | 80 |
|  |  | 9 | 20.90 | 60.45 | 69.10 | 76.45 | 93.00 | 81 |
|  |  | 12 | 0.00 | 60.00 | 71.10 | 75.90 | 91.70 | 75 |
|  | CHT | 0 | 31.60 | 53.30 | 64.65 | 73.90 | 89.10 | 46 |
|  |  | 3 | 0.00 | 51.03 | 60.95 | 77.60 | 94.00 | 44 |
|  |  | 6 | 31.00 | 61.60 | 67.80 | 76.90 | 91.50 | 43 |
|  |  | 9 | 31.70 | 65.20 | 70.60 | 77.60 | 93.00 | 45 |
|  |  | 12 | 0.00 | 60.00 | 70.80 | 75.90 | 89.00 | 43 |
|  | NCHT | 0 | 20.00 | 47.20 | 56.15 | 65.48 | 79.30 | 28 |
|  |  | 3 | 18.80 | 57.70 | 64.30 | 69.75 | 80.60 | 28 |
|  |  | 6 | 21.20 | 56.75 | 65.60 | 75.05 | 86.90 | 37 |
|  |  | 9 | 20.90 | 57.95 | 66.95 | 75.18 | 88.10 | 36 |
|  |  | 12 | 28.30 | 60.23 | 71.30 | 75.53 | 91.70 | 32 |
| Th2 CD3+CD4+CD194+CD294+ relative amount of CD3+CD4+ [%] | all | 0 | 0.03 | 0.41 | 0.84 | 2.68 | 72.90 | 74 |
|  |  | 3 | 0.06 | 0.38 | 0.91 | 2.72 | 32.40 | 72 |
|  |  | 6 | 0.06 | 0.54 | 1.14 | 3.05 | 74.00 | 80 |
|  |  | 9 | 0.09 | 0.61 | 1.51 | 3.28 | 72.60 | 81 |
|  |  | 12 | 0.07 | 0.64 | 1.61 | 3.96 | 77.70 | 75 |
|  | CHT | 0 | 0.10 | 0.41 | 0.84 | 2.75 | 72.90 | 46 |
|  |  | 3 | 0.06 | 0.30 | 0.91 | 2.72 | 32.40 | 44 |
|  |  | 6 | 0.06 | 0.53 | 1.13 | 2.99 | 74.00 | 43 |
|  |  | 9 | 0.14 | 0.74 | 1.32 | 3.00 | 72.60 | 45 |
|  |  | 12 | 0.07 | 0.50 | 1.74 | 3.96 | 77.70 | 43 |
|  | NCHT | 0 | 0.03 | 0.37 | 0.84 | 2.37 | 18.50 | 28 |
|  |  | 3 | 0.06 | 0.39 | 0.91 | 3.16 | 11.80 | 28 |
|  |  | 6 | 0.11 | 0.56 | 1.14 | 4.29 | 12.30 | 37 |
|  |  | 9 | 0.09 | 0.51 | 1.58 | 4.04 | 18.50 | 36 |
|  |  | 12 | 0.07 | 0.66 | 1.26 | 4.69 | 9.46 | 32 |
| naive Th2 CD3+CD4+CD194+CD294+CD45RO- relative amount of CD3+CD4+CD194+CD294+ [%] | all | 0 | 0.00 | 11.78 | 21.00 | 30.75 | 75.00 | 74 |
|  |  | 3 | 0.00 | 10.30 | 17.60 | 26.88 | 65.50 | 72 |
|  |  | 6 | 2.06 | 7.85 | 14.05 | 22.63 | 65.50 | 80 |
|  |  | 9 | 0.00 | 6.56 | 10.30 | 19.75 | 60.00 | 81 |
|  |  | 12 | 0.00 | 6.54 | 10.80 | 17.30 | 48.60 | 75 |
|  | CHT | 0 | 3.83 | 14.78 | 21.40 | 31.45 | 54.80 | 46 |
|  |  | 3 | 0.00 | 7.78 | 17.30 | 26.88 | 65.50 | 44 |
|  |  | 6 | 2.47 | 7.45 | 13.80 | 26.50 | 65.50 | 43 |
|  |  | 9 | 0.00 | 5.72 | 9.89 | 17.65 | 54.00 | 45 |
|  |  | 12 | 0.00 | 4.65 | 11.60 | 17.30 | 48.60 | 43 |
|  | NCHT | 0 | 0.00 | 11.63 | 20.75 | 31.65 | 75.00 | 28 |
|  |  | 3 | 4.82 | 11.78 | 17.80 | 27.10 | 51.90 | 28 |
|  |  | 6 | 2.06 | 8.34 | 14.40 | 21.15 | 42.90 | 37 |
|  |  | 9 | 3.24 | 6.96 | 12.75 | 21.48 | 60.00 | 36 |
|  |  | 12 | 3.57 | 8.41 | 10.65 | 17.68 | 30.00 | 32 |

**Table S2 (continued).** Descriptive statistics of flow cytometry data (treatment)

| cell population | group | time of assessmen  [months]t | minimum | Q1 | | median | | Q3 | | maximum | | n | |
| --- | --- | --- | --- | --- | --- | --- | --- | --- | --- | --- | --- | --- | --- |
| memory Th2 CD3+CD4+CD194+CD294+CD45RO+ relative amount of CD3+CD4+CD194+CD294+ [%] | all | 0 | 25.00 | 69.25 | | 79.00 | | 88.23 | | 100.00 | | 74 | |
|  |  | 3 | 34.50 | 73.13 | | 82.40 | | 89.50 | | 100.00 | | 72 | |
|  |  | 6 | 34.50 | 77.38 | | 85.95 | | 92.15 | | 97.90 | | 80 | |
|  |  | 9 | 40.00 | 80.25 | | 89.70 | | 93.40 | | 100.00 | | 81 | |
|  |  | 12 | 51.40 | 82.70 | | 89.20 | | 93.50 | | 100.00 | | 75 | |
|  | CHT | 0 | 45.20 | 68.55 | | 78.60 | | 85.23 | | 96.20 | | 46 | |
|  |  | 3 | 34.50 | 73.13 | | 82.70 | | 91.05 | | 100.00 | | 44 | |
|  |  | 6 | 34.50 | 73.50 | | 86.20 | | 92.50 | | 97.50 | | 43 | |
|  |  | 9 | 46.00 | 82.35 | | 90.10 | | 94.25 | | 100.00 | | 45 | |
|  |  | 12 | 51.40 | 82.70 | | 88.40 | | 95.30 | | 100.00 | | 43 | |
|  | NCHT | 0 | 25.00 | 68.35 | | 79.25 | | 88.38 | | 100.00 | | 28 | |
|  |  | 3 | 48.10 | 72.90 | | 82.20 | | 88.23 | | 95.20 | | 28 | |
|  |  | 6 | 57.10 | 78.85 | | 85.60 | | 91.65 | | 97.90 | | 37 | |
|  |  | 9 | 40.00 | 78.53 | | 87.25 | | 93.05 | | 96.80 | | 36 | |
|  |  | 12 | 70.00 | 82.33 | | 89.35 | | 91.63 | | 96.40 | | 32 | |
| Treg CD3+CD4+CD25+CD127- relative amount of CD3+CD4+ [%] | all | 0 | 1.35 | 3.49 | 4.26 | | 7.40 | | 53.70 | | 74 | |  |
|  |  | 3 | 1.35 | 3.57 | 4.77 | | 7.25 | | 39.00 | | 72 | |  |
|  |  | 6 | 1.21 | 4.34 | 5.63 | | 8.32 | | 53.60 | | 80 | |  |
|  |  | 9 | 1.60 | 4.53 | 6.20 | | 8.07 | | 33.10 | | 81 | |  |
|  |  | 12 | 1.68 | 4.09 | 6.03 | | 8.16 | | 27.30 | | 75 | |  |
|  | CHT | 0 | 1.50 | 3.50 | 4.22 | | 6.26 | | 51.80 | | 46 | |  |
|  |  | 3 | 2.19 | 3.53 | 4.26 | | 6.41 | | 25.60 | | 44 | |  |
|  |  | 6 | 1.79 | 4.04 | 5.39 | | 7.51 | | 53.60 | | 43 | |  |
|  |  | 9 | 2.22 | 4.51 | 6.00 | | 8.04 | | 33.10 | | 45 | |  |
|  |  | 12 | 2.43 | 4.10 | 6.02 | | 8.87 | | 13.10 | | 43 | |  |
|  | NCHT | 0 | 1.35 | 3.48 | 4.92 | | 17.50 | | 53.70 | | 28 | |  |
|  |  | 3 | 1.35 | 3.58 | 6.04 | | 12.63 | | 39.00 | | 28 | |  |
|  |  | 6 | 1.21 | 4.40 | 6.29 | | 11.45 | | 47.60 | | 37 | |  |
|  |  | 9 | 1.60 | 4.51 | 6.46 | | 9.09 | | 29.80 | | 36 | |  |
|  |  | 12 | 1.68 | 3.74 | 6.25 | | 8.05 | | 27.30 | | 32 | |  |
| Th1 / Th2 ratio | all | 0 | 0.01 | 0.84 | 1.23 | | 3.46 | | 18.40 | | 74 | |  |
|  |  | 3 | 0.00 | 0.80 | 1.33 | | 3.34 | | 7.86 | | 72 | |  |
|  |  | 6 | 0.00 | 0.62 | 1.51 | | 2.45 | | 29.18 | | 80 | |  |
|  |  | 9 | 0.00 | 0.53 | 1.11 | | 2.16 | | 19.65 | | 81 | |  |
|  |  | 12 | 0.00 | 0.45 | 0.95 | | 1.98 | | 24.09 | | 75 | |  |
|  | CHT | 0 | 0.01 | 0.87 | 1.22 | | 2.87 | | 6.76 | | 46 | |  |
|  |  | 3 | 0.00 | 0.74 | 1.21 | | 2.95 | | 7.86 | | 44 | |  |
|  |  | 6 | 0.00 | 0.64 | 1.61 | | 2.69 | | 10.62 | | 43 | |  |
|  |  | 9 | 0.00 | 0.46 | 1.21 | | 2.37 | | 18.82 | | 45 | |  |
|  |  | 12 | 0.00 | 0.36 | 0.91 | | 1.84 | | 12.69 | | 43 | |  |
|  | NCHT | 0 | 0.20 | 0.80 | 1.47 | | 5.33 | | 18.40 | | 28 | |  |
|  |  | 3 | 0.24 | 0.87 | 1.41 | | 4.03 | | 7.49 | | 28 | |  |
|  |  | 6 | 0.10 | 0.61 | 1.19 | | 2.36 | | 29.18 | | 37 | |  |
|  |  | 9 | 0.17 | 0.59 | 1.08 | | 1.86 | | 19.65 | | 36 | |  |
|  |  | 12 | 0.11 | 0.55 | 1.14 | | 2.16 | | 24.09 | | 32 | |  |

**Table S2 (continued).** Descriptive statistics of flow cytometry data (treatment)

| cell population | group | time of assessment  [months] | minimum | Q1 | median | Q3 | maximum | n |
| --- | --- | --- | --- | --- | --- | --- | --- | --- |
| Th / Treg ratio | all | 0 | 0.84 | 6.48 | 9.36 | 14.20 | 38.44 | 73 |
|  |  | 3 | 1.80 | 5.29 | 8.91 | 11.49 | 22.95 | 69 |
|  |  | 6 | 1.50 | 5.13 | 7.86 | 12.36 | 28.46 | 79 |
|  |  | 9 | 0.16 | 3.75 | 12.30 | 18.77 | 34.69 | 79 |
|  |  | 12 | 1.70 | 4.03 | 10.99 | 16.34 | 34.52 | 74 |
|  | CHT | 0 | 1.09 | 5.40 | 10.22 | 14.81 | 54.38 | 45 |
|  |  | 3 | 1.16 | 5.35 | 10.65 | 13.52 | 40.88 | 42 |
|  |  | 6 | 1.81 | 7.08 | 10.06 | 15.24 | 38.99 | 43 |
|  |  | 9 | 0.00 | 2.40 | 4.98 | 7.31 | 30.37 | 42 |
|  |  | 12 | 0.01 | 2.86 | 5.02 | 6.95 | 28.52 | 42 |
|  | NCHT | 0 | 0.00 | 2.34 | 4.27 | 6.99 | 23.80 | 28 |
|  |  | 3 | 0.00 | 2.60 | 3.90 | 5.66 | 26.04 | 27 |
|  |  | 6 | 0.00 | 2.55 | 4.74 | 7.06 | 25.11 | 37 |
|  |  | 9 | 0.49 | 3.64 | 5.02 | 7.31 | 17.25 | 36 |
|  |  | 12 | 1.25 | 3.80 | 6.03 | 7.22 | 24.38 | 32 |
| CTL / Treg ratio | all | 0 | 0.12 | 2.97 | 4.62 | 7.00 | 12.89 | 73 |
|  |  | 3 | 0.84 | 3.14 | 4.35 | 6.51 | 26.04 | 69 |
|  |  | 6 | 0.77 | 2.89 | 5.03 | 7.51 | 25.11 | 79 |
|  |  | 9 | 0.00 | 1.67 | 4.25 | 7.52 | 30.37 | 79 |
|  |  | 12 | 0.01 | 1.24 | 3.25 | 6.20 | 28.52 | 74 |
|  | CHT | 0 | 0.00 | 1.66 | 2.79 | 7.01 | 23.80 | 45 |
|  |  | 3 | 0.00 | 2.04 | 3.32 | 5.25 | 20.81 | 42 |
|  |  | 6 | 0.00 | 2.38 | 3.89 | 6.61 | 18.81 | 43 |
|  |  | 9 | 0.84 | 6.48 | 9.36 | 14.20 | 38.44 | 42 |
|  |  | 12 | 1.80 | 5.29 | 8.91 | 11.49 | 22.95 | 42 |
|  | NCHT | 0 | 1.50 | 5.13 | 7.86 | 12.36 | 28.46 | 28 |
|  |  | 3 | 0.16 | 3.75 | 12.30 | 18.77 | 34.69 | 27 |
|  |  | 6 | 1.70 | 4.03 | 10.99 | 16.34 | 34.52 | 37 |
|  |  | 9 | 1.09 | 5.40 | 10.22 | 14.81 | 54.38 | 36 |
|  |  | 12 | 1.16 | 5.35 | 10.65 | 13.52 | 40.88 | 32 |

**Table S3.** Descriptive statistics of multiplex immunoassay data (treatment)

| cytokine | group | time of  assessment  [months] | minimum | Q1 | median | Q3 | maximum | n |
| --- | --- | --- | --- | --- | --- | --- | --- | --- |
| IL-4 [pg/ml] | all | 0 | 2.01 | 21.05 | 34.92 | 57.40 | 1327.68 | 86 |
|  |  | 3 | 0.12 | 17.59 | 34.15 | 81.44 | 845.59 | 77 |
|  |  | 6 | 0.12 | 20.90 | 42.12 | 67.54 | 1579.89 | 80 |
|  |  | 9 | 1.88 | 18.50 | 42.71 | 89.87 | 1672.32 | 87 |
|  |  | 12 | 1.19 | 23.38 | 38.07 | 65.33 | 1714.91 | 82 |
|  | CHT | 0 | 7.97 | 20.16 | 31.56 | 67.32 | 1327.68 | 51 |
|  |  | 3 | 0.12 | 14.48 | 33.92 | 81.16 | 845.59 | 48 |
|  |  | 6 | 0.12 | 20.20 | 37.24 | 78.22 | 1579.89 | 45 |
|  |  | 9 | 1.88 | 15.89 | 37.47 | 98.82 | 1672.32 | 50 |
|  |  | 12 | 5.95 | 22.78 | 30.01 | 58.27 | 1714.91 | 46 |
|  | NCHT | 0 | 2.01 | 25.63 | 36.01 | 56.72 | 628.89 | 35 |
|  |  | 3 | 1.65 | 21.34 | 34.15 | 82.54 | 588.57 | 29 |
|  |  | 6 | 1.04 | 24.53 | 47.36 | 64.04 | 594.38 | 35 |
|  |  | 9 | 3.00 | 26.06 | 42.94 | 92.48 | 704.16 | 37 |
|  |  | 12 | 1.19 | 27.27 | 44.06 | 73.00 | 607.82 | 36 |
| IL-7 [pg/ml] | all | 0 | 1.88 | 6.31 | 9.35 | 12.10 | 20.47 | 86 |
|  |  | 3 | 1.62 | 5.54 | 8.52 | 10.93 | 23.92 | 77 |
|  |  | 6 | 1.83 | 5.68 | 8.49 | 11.41 | 25.93 | 80 |
|  |  | 9 | 1.15 | 5.20 | 8.07 | 11.19 | 24.87 | 87 |
|  |  | 12 | 1.63 | 5.54 | 8.69 | 11.17 | 67.65 | 82 |
|  | CHT | 0 | 1.94 | 6.05 | 8.96 | 12.83 | 20.47 | 51 |
|  |  | 3 | 1.65 | 5.22 | 7.74 | 10.75 | 23.92 | 48 |
|  |  | 6 | 2.07 | 5.13 | 6.99 | 10.70 | 25.93 | 45 |
|  |  | 9 | 1.15 | 4.83 | 7.42 | 10.09 | 24.87 | 50 |
|  |  | 12 | 1.87 | 5.37 | 8.13 | 9.17 | 20.08 | 46 |
|  | NCHT | 0 | 1.88 | 6.31 | 9.65 | 11.94 | 19.00 | 35 |
|  |  | 3 | 1.62 | 6.63 | 9.94 | 11.03 | 21.72 | 29 |
|  |  | 6 | 1.83 | 6.93 | 9.55 | 11.56 | 20.48 | 35 |
|  |  | 9 | 1.98 | 5.71 | 10.57 | 12.23 | 20.51 | 37 |
|  |  | 12 | 1.63 | 5.63 | 9.94 | 12.83 | 67.65 | 36 |
| IL-8 [pg/ml] | all | 0 | 0.13 | 1.84 | 3.23 | 6.14 | 80.82 | 86 |
|  |  | 3 | 0.13 | 2.50 | 3.61 | 8.44 | 75.25 | 77 |
|  |  | 6 | 0.13 | 2.48 | 3.89 | 8.58 | 118.10 | 80 |
|  |  | 9 | 0.13 | 2.35 | 3.89 | 12.03 | 157.90 | 87 |
|  |  | 12 | 0.41 | 2.29 | 3.34 | 8.70 | 189.40 | 82 |
|  | CHT | 0 | 0.13 | 1.84 | 3.56 | 7.31 | 80.82 | 51 |
|  |  | 3 | 0.13 | 2.48 | 3.36 | 8.58 | 73.67 | 48 |
|  |  | 6 | 0.13 | 2.62 | 4.05 | 10.58 | 118.06 | 45 |
|  |  | 9 | 0.13 | 2.35 | 4.10 | 11.69 | 157.91 | 50 |
|  |  | 12 | 0.87 | 2.26 | 3.68 | 7.58 | 189.40 | 46 |
|  | NCHT | 0 | 0.35 | 1.71 | 3.17 | 6.14 | 66.93 | 35 |
|  |  | 3 | 0.13 | 2.44 | 4.22 | 6.96 | 75.25 | 29 |
|  |  | 6 | 0.61 | 1.99 | 3.72 | 7.35 | 102.67 | 35 |
|  |  | 9 | 0.38 | 2.60 | 3.80 | 13.30 | 137.58 | 37 |
|  |  | 12 | 0.41 | 2.34 | 3.23 | 10.86 | 141.66 | 36 |

**Table S3 (continued).** Descriptive statistics of multiplex immunoassay data (treatment)

| cytokine | group | time of  assessment  [months] | minimum | Q1 | median | Q3 | maximum | n |
| --- | --- | --- | --- | --- | --- | --- | --- | --- |
| IL-10 [pg/ml] | all | 0 | 1.72 | 11.94 | 20.47 | 31.46 | 222.90 | 86 |
|  |  | 3 | 1.59 | 10.22 | 22.69 | 41.16 | 144.20 | 77 |
|  |  | 6 | 3.03 | 11.64 | 23.16 | 37.96 | 262.00 | 80 |
|  |  | 9 | 0.56 | 12.21 | 24.14 | 39.99 | 274.70 | 87 |
|  |  | 12 | 1.78 | 11.25 | 22.44 | 35.55 | 457.30 | 82 |
|  | CHT | 0 | 4.42 | 11.07 | 20.02 | 34.99 | 222.90 | 51 |
|  |  | 3 | 1.79 | 9.34 | 22.10 | 42.19 | 144.24 | 48 |
|  |  | 6 | 3.03 | 11.34 | 24.05 | 34.14 | 261.97 | 45 |
|  |  | 9 | 3.06 | 11.21 | 20.45 | 51.41 | 274.68 | 50 |
|  |  | 12 | 3.32 | 10.35 | 18.16 | 31.85 | 242.76 | 46 |
|  | NCHT | 0 | 1.72 | 11.94 | 23.46 | 30.18 | 116.45 | 35 |
|  |  | 3 | 1.59 | 10.82 | 23.12 | 39.35 | 101.33 | 29 |
|  |  | 6 | 3.13 | 12.89 | 21.01 | 44.30 | 95.93 | 35 |
|  |  | 9 | 0.56 | 12.93 | 27.97 | 37.00 | 118.84 | 37 |
|  |  | 12 | 1.78 | 14.21 | 26.74 | 40.95 | 457.25 | 36 |
| IL-12(p70) [pg/ml] | all | 0 | 1.09 | 3.36 | 5.03 | 8.16 | 16.97 | 86 |
|  |  | 3 | 0.65 | 2.74 | 5.03 | 7.35 | 19.17 | 77 |
|  |  | 6 | 1.07 | 2.87 | 4.89 | 7.28 | 15.03 | 80 |
|  |  | 9 | 0.38 | 2.96 | 4.56 | 7.66 | 21.64 | 87 |
|  |  | 12 | 0.87 | 3.31 | 4.49 | 7.14 | 118.10 | 82 |
|  | CHT | 0 | 1.22 | 3.22 | 5.04 | 8.33 | 16.97 | 51 |
|  |  | 3 | 0.65 | 2.47 | 4.91 | 7.23 | 13.57 | 48 |
|  |  | 6 | 1.07 | 2.67 | 4.80 | 6.55 | 15.03 | 45 |
|  |  | 9 | 0.38 | 2.64 | 4.02 | 6.57 | 11.89 | 50 |
|  |  | 12 | 0.90 | 3.17 | 4.02 | 5.74 | 31.33 | 46 |
|  | NCHT | 0 | 1.09 | 3.55 | 4.90 | 7.81 | 13.20 | 35 |
|  |  | 3 | 1.19 | 3.46 | 5.63 | 8.12 | 19.17 | 29 |
|  |  | 6 | 1.15 | 3.36 | 6.45 | 8.28 | 11.79 | 35 |
|  |  | 9 | 1.16 | 3.13 | 4.95 | 8.26 | 21.64 | 37 |
|  |  | 12 | 0.87 | 3.45 | 5.64 | 7.92 | 118.11 | 36 |
| IFN-γ [pg/ml] | all | 0 | 2.62 | 13.86 | 21.70 | 32.50 | 74.54 | 86 |
|  |  | 3 | 2.05 | 11.12 | 20.39 | 30.77 | 57.28 | 77 |
|  |  | 6 | 2.67 | 12.24 | 19.16 | 28.25 | 56.62 | 80 |
|  |  | 9 | 2.01 | 12.69 | 19.14 | 29.38 | 49.34 | 87 |
|  |  | 12 | 2.04 | 12.06 | 20.55 | 28.78 | 46.85 | 82 |
|  | CHT | 0 | 4.96 | 13.31 | 23.01 | 34.41 | 74.54 | 51 |
|  |  | 3 | 2.05 | 9.04 | 18.02 | 29.30 | 57.28 | 48 |
|  |  | 6 | 4.26 | 10.83 | 18.00 | 27.54 | 48.36 | 45 |
|  |  | 9 | 2.01 | 10.61 | 19.79 | 29.69 | 49.34 | 50 |
|  |  | 12 | 2.79 | 11.86 | 20.36 | 27.49 | 46.85 | 46 |
|  | NCHT | 0 | 2.62 | 14.15 | 21.45 | 29.50 | 49.79 | 35 |
|  |  | 3 | 4.34 | 14.31 | 22.05 | 31.45 | 49.55 | 29 |
|  |  | 6 | 2.67 | 14.69 | 19.69 | 29.48 | 56.62 | 35 |
|  |  | 9 | 2.53 | 14.25 | 18.09 | 29.09 | 44.80 | 37 |
|  |  | 12 | 2.04 | 12.79 | 21.37 | 30.39 | 44.64 | 36 |

**Table S3 (continued).** Descriptive statistics of multiplex immunoassay data (treatment)

| cytokine | group | time of  assessment  [months] | minimum | Q1 | median | Q3 | maximum | n |
| --- | --- | --- | --- | --- | --- | --- | --- | --- |
| TNF [pg/ml] | all | 0 | 2.86 | 6.35 | 8.38 | 11.27 | 18.08 | 86 |
|  |  | 3 | 1.29 | 7.26 | 8.78 | 11.32 | 22.42 | 77 |
|  |  | 6 | 2.83 | 6.61 | 9.21 | 11.95 | 21.23 | 80 |
|  |  | 9 | 1.54 | 6.48 | 8.91 | 10.94 | 20.36 | 87 |
|  |  | 12 | 2.43 | 6.40 | 8.57 | 11.09 | 18.90 | 82 |
|  | CHT | 0 | 2.86 | 6.82 | 8.98 | 11.70 | 16.55 | 51 |
|  |  | 3 | 3.20 | 7.60 | 9.67 | 12.00 | 22.42 | 48 |
|  |  | 6 | 2.83 | 6.79 | 9.88 | 12.32 | 21.21 | 45 |
|  |  | 9 | 2.25 | 6.55 | 9.00 | 11.27 | 20.36 | 50 |
|  |  | 12 | 2.43 | 6.25 | 8.50 | 10.27 | 18.90 | 46 |
|  | NCHT | 0 | 4.21 | 6.08 | 7.96 | 9.93 | 18.08 | 35 |
|  |  | 3 | 1.29 | 6.39 | 7.66 | 9.89 | 14.25 | 29 |
|  |  | 6 | 3.61 | 6.49 | 7.71 | 10.74 | 21.23 | 35 |
|  |  | 9 | 1.54 | 6.24 | 8.87 | 10.69 | 19.68 | 37 |
|  |  | 12 | 3.37 | 6.46 | 9.36 | 12.00 | 18.18 | 36 |

**Table S4.** Patient undergoing neoadjuvant chemotherapy characteristics: age. tumor entity. grading. tumor stage (TNM-classification) and type of therapy: chemotherapy (CHT) / no chemotherapy (NCHT). “NST“= No special type. *Grading not available in one NCHT patient. ^#^Fisher-Freeman-Halton’s exact test. ^‡^Fisher’s exact test

|  | | **all patients undergoing  neoadjuvant chemotherapy** | | | **pCR** | | **non-pCR** | | **p-value** |
| --- | --- | --- | --- | --- | --- | --- | --- | --- | --- |
| Age (median. min/max) | | 55.0 (27 / 75 years) | | | 56.0 (27 / 58 years) | | 55.0 (33 / 75 years) | |  |
|  | | n | percentage | n | | percentage | n | Percentage | - |
| Total | | 34 | 100.0% | 12 | | 34.3% | 22 | 65.7% |  |
| *Tumor*  *entity* | NST | 29 | 82.9% | 11 | | 91.7% | 18 | 81.8% | 0.588^#^ |
|  | invasiv lobular | 2 | 5.7% | 1 | | 8.3% | 1 | 4.5% |  |
|  | others | 3 | 8.6% | 0 | | 0.0% | 3 | 13.6% |  |
| *cT* | cT0* | 0 | 0.0% | 0 | | 0.0% | 0 | 0.0% | 0.635^#^ |
|  | cT1 | 13 | 37.1% | 5 | | 41.7% | 8 | 36.4% |  |
|  | cT2 | 18 | 51.4% | 6 | | 50.0% | 12 | 54.5% |  |
|  | cT3 | 1 | 2.9% | 1 | | 8.3% | 0 | 0.0% |  |
|  | cT4 | 2 | 5.7% | 0 | | 0.0% | 2 | 9.1% |  |
| *cN* | cN0 | 20 | 57.1% | 8 | | 66.7% | 12 | 54.5% | 0.489^‡^ |
|  | cN+ | 14 | 40.0% | 4 | | 33.3% | 10 | 45.5% |  |
| *M* | M0 | 34 | 97.1% | 12 | | 100.0% | 22 | 95.7% | - |
| *Grading* | G1 | 0 | 0.0% | 0 | | 0.0% | 0 | 0.0% | >0.999 |
|  | G2 | 11 | 31.4% | 4 | | 33.3% | 7 | 31.8% |  |
|  | G3 | 23 | 65.7% | 8 | | 66.7% | 15 | 65.2% |  |
| *Receptor* | Luminal A | 0 | 0.0% | 0 | | 0.0% | 0 | 0.0% | 0.005^#^ |
|  | Luminal B | 9 | 26.5% | 1 | | 8.3% | 9 | 40.9% |  |
|  | HER2 positive | 17 | 50.0% | 10 | | 83.3% | 7 | 31.8% |  |
|  | Triple negative | 8 | 23.5% | 2 | | 16.7% | 6 | 27.3% |  |

**Table S5.** Descriptive statistics of flow cytometry data (outcome)

| cell  population | group | time of  assessment | minimum | Q1 | median | Q3 | maximum | n |
| --- | --- | --- | --- | --- | --- | --- | --- | --- |
| T cells CD3+ relative amount of viable cells [%] | pCR | 0 | 23.30 | 29.20 | 39.30 | 45.30 | 68.60 | 11 |
|  |  | 3 | 16.30 | 25.90 | 34.80 | 67.20 | 71.80 | 9 |
|  |  | 6 | 14.70 | 20.58 | 39.50 | 46.48 | 76.90 | 10 |
|  |  | 9 | 20.90 | 25.98 | 41.20 | 47.15 | 67.80 | 10 |
|  |  | 12 | 10.20 | 19.30 | 26.00 | 50.65 | 64.60 | 9 |
|  | non-pCR | 0 | 18.5 | 38 | 54.3 | 60.75 | 64.2 | 18 |
|  |  | 3 | 14.8 | 34.3 | 39.9 | 59.4 | 65.4 | 17 |
|  |  | 6 | 1.9 | 38.75 | 48.35 | 56.7 | 74.4 | 22 |
|  |  | 9 | 15 | 35.8 | 53.2 | 57.7 | 66.8 | 19 |
|  |  | 12 | 19.3 | 32.5 | 39.6 | 55.7 | 64.4 | 15 |
| Th  CD3+CD4+ relative amount of CD3+ [%] | pCR | 0 | 41.30 | 47.50 | 61.40 | 69.50 | 76.10 | 11 |
|  |  | 3 | 20.70 | 44.65 | 51.90 | 65.35 | 75.60 | 9 |
|  |  | 6 | 25.70 | 39.18 | 47.75 | 65.28 | 73.50 | 10 |
|  |  | 9 | 41.20 | 45.85 | 51.10 | 64.73 | 82.70 | 10 |
|  |  | 12 | 23.20 | 37.75 | 48.00 | 64.15 | 65.60 | 9 |
|  | non-pCR | 0 | 42.1 | 49.18 | 61.2 | 72.1 | 84.1 | 18 |
|  |  | 3 | 24.5 | 39.35 | 54.9 | 62.25 | 83.9 | 17 |
|  |  | 6 | 26.6 | 44.58 | 53.45 | 63.1 | 78.7 | 22 |
|  |  | 9 | 19 | 39.7 | 53.5 | 62.6 | 79.4 | 19 |
|  |  | 12 | 16.7 | 39.1 | 55 | 64.7 | 79.7 | 15 |
| CTL CD3+CD8+ relative amount of CD3+ [%] | pCR | 0 | 13.90 | 17.20 | 21.80 | 27.00 | 37.40 | 11 |
|  |  | 3 | 19.80 | 20.00 | 23.90 | 38.75 | 60.60 | 9 |
|  |  | 6 | 20.10 | 20.70 | 26.10 | 35.38 | 46.60 | 10 |
|  |  | 9 | 13.30 | 19.40 | 29.05 | 36.23 | 42.60 | 10 |
|  |  | 12 | 18.60 | 22.45 | 29.20 | 34.65 | 53.30 | 9 |
|  | non-pCR | 0 | 6.37 | 18.15 | 24.95 | 37.38 | 44.2 | 18 |
|  |  | 3 | 13.5 | 20.85 | 27.3 | 40.95 | 53.4 | 17 |
|  |  | 6 | 0.65 | 21.18 | 29.55 | 37.93 | 44 | 22 |
|  |  | 9 | 13.7 | 25.8 | 27.9 | 45.3 | 57.8 | 19 |
|  |  | 12 | 6.1 | 24.5 | 32.3 | 43.3 | 58.4 | 15 |
| Treg CD3+CD4+CD25+CD127- relative amount of CD3+CD4+ [%] | pCR | 0 | 1.73 | 2.67 | 3.97 | 7.62 | 22.40 | 11 |
|  |  | 3 | 2.84 | 4.45 | 5.84 | 8.72 | 22.60 | 9 |
|  |  | 6 | 2.59 | 4.30 | 4.84 | 8.05 | 10.30 | 10 |
|  |  | 9 | 3.88 | 4.43 | 5.05 | 9.38 | 10.40 | 11 |
|  |  | 12 | 3.03 | 4.06 | 6.06 | 9.88 | 11.30 | 9 |
|  | non-pCR | 0 | 1.5 | 3.695 | 4.565 | 5.913 | 51.8 | 18 |
|  |  | 3 | 2.19 | 3.475 | 4.19 | 5.155 | 25.6 | 17 |
|  |  | 6 | 1.79 | 4.51 | 5.52 | 7.51 | 53.6 | 19 |
|  |  | 9 | 2.22 | 3.8 | 6.46 | 8.09 | 33.1 | 19 |
|  |  | 12 | 2.43 | 4.745 | 5.965 | 7.45 | 12.6 | 16 |

**Table S5 (continued).** Descriptive statistics of flow cytometry data (outcome)

| cell  population | group | time of  assessment | minimum | Q1 | median | Q3 | maximum | n |
| --- | --- | --- | --- | --- | --- | --- | --- | --- |
| naive effector Th CD3+CD4+CD45RO-CD62L- relative amount of CD3+CD4+ [%] | pCR | 0 | 9.98 | 18.40 | 33.70 | 39.00 | 53.80 | 11 |
|  |  | 3 | 3.16 | 5.77 | 13.60 | 29.40 | 38.00 | 9 |
|  |  | 6 | 3.91 | 5.21 | 9.62 | 15.13 | 36.00 | 10 |
|  |  | 9 | 1.05 | 3.93 | 12.29 | 20.73 | 23.20 | 10 |
|  |  | 12 | 1.57 | 4.33 | 8.25 | 13.80 | 27.20 | 9 |
|  | non-pCR | 0 | 5.26 | 19.3 | 27.2 | 31.43 | 60.1 | 18 |
|  |  | 3 | 2.88 | 9.63 | 18.5 | 34.8 | 51.5 | 17 |
|  |  | 6 | 2.25 | 11.3 | 17.55 | 27.1 | 39.2 | 22 |
|  |  | 9 | 1.97 | 7.88 | 12.7 | 18.3 | 41.7 | 19 |
|  |  | 12 | 1.4 | 8.23 | 13.6 | 23 | 31.7 | 15 |
| memory effector Th CD3+CD4+CD45RO+CD62L- relative amount of CD3+CD4+ [%] | pCR | 0 | 6.30 | 25.10 | 31.90 | 47.50 | 74.40 | 11 |
|  |  | 3 | 5.61 | 33.45 | 39.20 | 58.10 | 74.80 | 9 |
|  |  | 6 | 6.08 | 38.45 | 60.70 | 66.58 | 77.80 | 10 |
|  |  | 9 | 30.30 | 43.20 | 52.70 | 68.70 | 81.60 | 10 |
|  |  | 12 | 28.80 | 36.25 | 55.50 | 65.15 | 71.50 | 9 |
|  | non-pCR | 0 | 21.7 | 30.88 | 41.75 | 55.35 | 63.3 | 18 |
|  |  | 3 | 31.3 | 37.65 | 43.1 | 50.45 | 64.5 | 17 |
|  |  | 6 | 3.44 | 46.25 | 52.3 | 61.3 | 70.7 | 22 |
|  |  | 9 | 33.5 | 52.4 | 58.5 | 72.8 | 79.7 | 19 |
|  |  | 12 | 33.9 | 42.5 | 51 | 58.7 | 78.1 | 15 |
| naive central Th CD3+CD4+CD45RO-CD62L+ relative amount of CD3+CD4+ [%] | pCR | 0 | 2.38 | 10.60 | 13.70 | 22.90 | 29.40 | 11 |
|  |  | 3 | 0.72 | 3.73 | 10.60 | 17.05 | 35.40 | 9 |
|  |  | 6 | 0.33 | 2.26 | 3.95 | 11.15 | 28.90 | 10 |
|  |  | 9 | 0.29 | 1.40 | 5.27 | 12.25 | 26.70 | 10 |
|  |  | 12 | 0.64 | 2.67 | 4.49 | 14.15 | 19.80 | 9 |
|  | non-pCR | 0 | 1.14 | 3.643 | 9.16 | 19.65 | 31.5 | 18 |
|  |  | 3 | 1.42 | 4.02 | 7.13 | 12.3 | 19.8 | 17 |
|  |  | 6 | 1.34 | 2.26 | 3.81 | 10.35 | 45.5 | 22 |
|  |  | 9 | 0.51 | 1.17 | 2.08 | 8.2 | 16.7 | 19 |
|  |  | 12 | 0.47 | 2.15 | 4.27 | 15 | 18.6 | 15 |
| memory central Th CD3+CD4+CD45RO+CD62L+ relative amount of CD3+CD4+ [%] | pCR | 0 | 3.48 | 7.99 | 16.95 | 21.25 | 26.50 | 11 |
|  |  | 3 | 8.73 | 11.60 | 26.10 | 33.85 | 58.60 | 9 |
|  |  | 6 | 15.40 | 18.23 | 25.30 | 29.45 | 34.90 | 10 |
|  |  | 9 | 6.66 | 13.78 | 26.65 | 40.48 | 59.00 | 10 |
|  |  | 12 | 14.60 | 19.35 | 31.70 | 40.40 | 48.50 | 9 |
|  | non-pCR | 0 | 3.48 | 10.03 | 15.8 | 20.4 | 58.7 | 18 |
|  |  | 3 | 4.35 | 14.75 | 23.6 | 35.25 | 52.6 | 17 |
|  |  | 6 | 3.87 | 16.08 | 20.9 | 29.05 | 45.4 | 22 |
|  |  | 9 | 2.49 | 9.74 | 19.9 | 28.5 | 41.2 | 19 |
|  |  | 12 | 7.84 | 15 | 21 | 39.5 | 49.5 | 15 |

**Table S5 (continued).** Descriptive statistics of flow cytometry data (outcome)

| cell  population | group | time of  assessment | minimum | Q1 | median | Q3 | maximum | n |
| --- | --- | --- | --- | --- | --- | --- | --- | --- |
| naive effector CTL CD3+CD8+CD45RO-CD62L- relative amount of CD3+CD4+ [%] | pCR | 0 | 30.70 | 31.50 | 53.90 | 64.60 | 76.40 | 11 |
|  |  | 3 | 26.00 | 27.15 | 39.70 | 60.70 | 65.80 | 9 |
|  |  | 6 | 20.10 | 24.38 | 33.95 | 50.63 | 73.20 | 10 |
|  |  | 9 | 19.00 | 25.90 | 33.45 | 54.90 | 66.30 | 10 |
|  |  | 12 | 19.80 | 25.05 | 37.90 | 42.60 | 60.90 | 9 |
|  | non-pCR | 0 | 32.5 | 42.3 | 52.25 | 59.05 | 65.9 | 18 |
|  |  | 3 | 22.5 | 32.1 | 54 | 70.7 | 79.4 | 17 |
|  |  | 6 | 26.7 | 36.43 | 46.15 | 56.18 | 74.2 | 22 |
|  |  | 9 | 20.5 | 33.3 | 43 | 64.7 | 82.2 | 19 |
|  |  | 12 | 22.1 | 45.5 | 48.9 | 56.3 | 77.2 | 15 |
| memory effector CTL CD3+CD8+CD45RO+CD62L- relative amount of CD3+CD8+ [%] | pCR | 0 | 16.40 | 18.40 | 28.50 | 39.70 | 63.40 | 11 |
|  |  | 3 | 5.61 | 33.45 | 39.20 | 58.10 | 74.80 | 9 |
|  |  | 6 | 14.70 | 32.00 | 43.80 | 53.63 | 70.50 | 10 |
|  |  | 9 | 10.20 | 26.78 | 41.40 | 53.08 | 69.30 | 10 |
|  |  | 12 | 11.70 | 29.05 | 44.70 | 55.60 | 69.60 | 9 |
|  | non-pCR | 0 | 12.8 | 24.93 | 31.85 | 37.83 | 45.5 | 18 |
|  |  | 3 | 13.5 | 18.75 | 26.5 | 33.95 | 54.1 | 17 |
|  |  | 6 | 14.3 | 24.9 | 30.95 | 42.1 | 61.3 | 22 |
|  |  | 9 | 13.3 | 27.6 | 35.8 | 51.4 | 64.1 | 19 |
|  |  | 12 | 15.4 | 27.3 | 36.1 | 41.2 | 57.8 | 15 |
| naive central CTL CD3+CD8+CD45RO-CD62L+ relative amount of CD3+CD8+ [%] | pCR | 0 | 1.60 | 5.18 | 12.40 | 19.40 | 21.80 | 11 |
|  |  | 3 | 1.20 | 5.61 | 12.90 | 27.85 | 34.00 | 9 |
|  |  | 6 | 0.00 | 1.88 | 6.38 | 15.63 | 44.60 | 10 |
|  |  | 9 | 2.42 | 2.89 | 8.22 | 20.30 | 30.10 | 10 |
|  |  | 12 | 3.04 | 4.39 | 9.55 | 16.35 | 33.90 | 9 |
|  | non-pCR | 0 | 0 | 4.998 | 12 | 19.48 | 29.1 | 18 |
|  |  | 3 | 2.68 | 4.755 | 11.8 | 19.9 | 31.8 | 17 |
|  |  | 6 | 1.33 | 3.678 | 9.875 | 17.6 | 50 | 22 |
|  |  | 9 | 1.05 | 2.12 | 6.65 | 14.4 | 28.4 | 19 |
|  |  | 12 | 0 | 3.01 | 6.58 | 12.5 | 24.5 | 15 |
| memory central CTL CD3+CD8+CD45RO+CD62L+ relative amount of CD3+CD8+ [%] | pCR | 0 | 0.52 | 2.14 | 4.30 | 5.98 | 14.10 | 11 |
|  |  | 3 | 0.89 | 3.05 | 4.82 | 15.10 | 18.70 | 9 |
|  |  | 6 | 2.24 | 3.18 | 5.62 | 9.41 | 21.70 | 10 |
|  |  | 9 | 1.31 | 3.22 | 5.69 | 15.15 | 23.30 | 10 |
|  |  | 12 | 2.48 | 3.61 | 7.23 | 15.30 | 18.70 | 9 |
|  | non-pCR | 0 | 0 | 2.708 | 4.07 | 5.363 | 14 | 18 |
|  |  | 3 | 0.68 | 2.12 | 4.84 | 12.35 | 20.9 | 17 |
|  |  | 6 | 2.32 | 3.095 | 4.745 | 9.913 | 17 | 22 |
|  |  | 9 | 0 | 2.51 | 3.73 | 7.37 | 21.4 | 19 |
|  |  | 12 | 2.25 | 3.12 | 5.42 | 8.37 | 15.5 | 15 |

**Table S5 (continued).** Descriptive statistics of flow cytometry data (outcome)

| cell  population | group | time of  assessment | minimum | Q1 | median | Q3 | maximum | n |
| --- | --- | --- | --- | --- | --- | --- | --- | --- |
| thymus negative Th CD3+CD4+CD45RO-CD31- relative amount of CD3+CD4+ [%] | pCR | 0 | 2.89 | 6.26 | 12.90 | 18.60 | 34.20 | 11 |
|  |  | 3 | 1.33 | 4.12 | 6.99 | 11.80 | 25.20 | 9 |
|  |  | 6 | 1.38 | 2.14 | 4.80 | 7.18 | 24.10 | 10 |
|  |  | 9 | 0.43 | 1.33 | 5.74 | 8.81 | 20.20 | 10 |
|  |  | 12 | 0.87 | 1.56 | 4.67 | 6.67 | 23.50 | 9 |
|  | non-pCR | 0 | 2.83 | 9.243 | 13.1 | 18.2 | 22.5 | 18 |
|  |  | 3 | 1.58 | 6.645 | 9.12 | 14.65 | 36.3 | 17 |
|  |  | 6 | 1.92 | 5.328 | 8.09 | 11.63 | 17 | 22 |
|  |  | 9 | 1.55 | 2.95 | 7.05 | 11.2 | 21.1 | 19 |
|  |  | 12 | 0.85 | 4.1 | 7.06 | 13.4 | 16.2 | 15 |
| thymus positive Th CD3+CD4+CD45RO-CD31+ relative amount of CD3+CD4+ [%] | pCR | 0 | 6.06 | 25.00 | 33.40 | 48.80 | 61.40 | 11 |
|  |  | 3 | 2.42 | 6.98 | 17.50 | 34.35 | 46.40 | 9 |
|  |  | 6 | 2.70 | 5.00 | 13.45 | 20.83 | 32.40 | 10 |
|  |  | 9 | 1.16 | 3.49 | 12.50 | 20.38 | 26.60 | 10 |
|  |  | 12 | 1.26 | 5.15 | 12.00 | 17.50 | 22.90 | 9 |
|  | non-pCR | 0 | 5.23 | 18.03 | 21.55 | 40.28 | 45.9 | 18 |
|  |  | 3 | 2.62 | 11.7 | 18.1 | 22.75 | 42.6 | 17 |
|  |  | 6 | 1.7 | 8.365 | 14.15 | 23.33 | 49.4 | 22 |
|  |  | 9 | 2.24 | 7.15 | 11.8 | 16.6 | 23.5 | 19 |
|  |  | 12 | 1.69 | 8.58 | 12.4 | 17.5 | 36.4 | 15 |
| Th1 CD3+CD4+CD183+CD196+ relative amount of CD3+CD4+ [%] | pCR | 0 | 0.44 | 0.57 | 1.70 | 3.24 | 5.78 | 11 |
|  |  | 3 | 0.00 | 0.47 | 1.16 | 2.55 | 5.26 | 9 |
|  |  | 6 | 0.16 | 0.57 | 2.48 | 3.62 | 4.09 | 10 |
|  |  | 9 | 0.10 | 0.23 | 2.05 | 3.28 | 4.10 | 11 |
|  |  | 12 | 0.24 | 0.58 | 1.38 | 1.94 | 4.22 | 9 |
|  | non-pCR | 0 | 0.16 | 0.558 | 1.805 | 3.045 | 7.06 | 18 |
|  |  | 3 | 0.1 | 0.355 | 1.62 | 3.255 | 4.39 | 17 |
|  |  | 6 | 0.17 | 0.53 | 2.89 | 4.04 | 6.07 | 19 |
|  |  | 9 | 0.15 | 0.79 | 2.08 | 3.92 | 7.53 | 19 |
|  |  | 12 | 0.02 | 0.235 | 0.865 | 3.973 | 7.33 | 16 |
| Th2 CD3+CD4+CD194+CD294+ relative amount of CD3+CD4+ [%] | pCR | 0 | 0.17 | 0.37 | 0.93 | 2.88 | 72.90 | 11 |
|  |  | 3 | 0.15 | 0.29 | 0.81 | 3.82 | 32.40 | 9 |
|  |  | 6 | 0.37 | 0.65 | 1.19 | 2.28 | 74.00 | 10 |
|  |  | 9 | 0.36 | 0.73 | 1.24 | 2.92 | 72.60 | 11 |
|  |  | 12 | 0.50 | 0.68 | 1.40 | 3.13 | 77.70 | 9 |
|  | non-pCR | 0 | 0.1 | 0.44 | 1.075 | 4.14 | 8.45 | 18 |
|  |  | 3 | 0.062 | 0.225 | 1.13 | 4.08 | 8.61 | 17 |
|  |  | 6 | 0.06 | 0.6 | 1.33 | 3.76 | 12.5 | 19 |
|  |  | 9 | 0.27 | 0.86 | 1.71 | 4.92 | 8.72 | 19 |
|  |  | 12 | 0.072 | 0.443 | 2.99 | 6.435 | 8.6 | 16 |

**Table S5 (continued).** Descriptive statistics of flow cytometry data (outcome)

| cell  population | group | time of  assessment | minimum | Q1 | median | Q3 | maximum | n |
| --- | --- | --- | --- | --- | --- | --- | --- | --- |
| naive Th1 CD3+CD4+CD183+CD196+CD45RO- relative amount of CD3+CD4+CD183+CD196+ [%] | pCR | 0 | 22.80 | 27.90 | 40.70 | 47.90 | 54.20 | 11 |
|  |  | 3 | 0.00 | 14.10 | 44.70 | 51.35 | 59.00 | 9 |
|  |  | 6 | 16.70 | 19.78 | 31.10 | 34.58 | 48.40 | 10 |
|  |  | 9 | 8.14 | 14.80 | 31.60 | 33.70 | 42.50 | 11 |
|  |  | 12 | 11.00 | 14.20 | 21.70 | 32.95 | 55.10 | 9 |
|  | non-pCR | 0 | 15.8 | 24.3 | 28.5 | 40.08 | 66.3 | 18 |
|  |  | 3 | 6.01 | 20.7 | 30.9 | 49.6 | 56 | 17 |
|  |  | 6 | 8.51 | 22.4 | 28.3 | 36.4 | 63.1 | 19 |
|  |  | 9 | 13.7 | 22.4 | 27.4 | 36 | 56.9 | 19 |
|  |  | 12 | 21.9 | 25.98 | 30.25 | 39.18 | 50 | 16 |
| memory Th1 CD3+CD4+CD183+CD196+CD45RO+ relative amount of CD3+CD4+CD183+CD196+ [%] | pCR | 0 | 45.80 | 52.10 | 59.30 | 72.10 | 77.20 | 11 |
|  |  | 3 | 0.00 | 42.00 | 55.20 | 76.70 | 89.00 | 9 |
|  |  | 6 | 51.60 | 65.43 | 68.90 | 80.23 | 83.30 | 10 |
|  |  | 9 | 57.50 | 66.30 | 68.40 | 85.20 | 91.90 | 11 |
|  |  | 12 | 44.90 | 67.05 | 78.30 | 85.80 | 89.00 | 9 |
|  | non-pCR | 0 | 33.7 | 59.93 | 71.5 | 75.7 | 84.2 | 18 |
|  |  | 3 | 44 | 50.4 | 69.1 | 79.3 | 94 | 17 |
|  |  | 6 | 36.9 | 63.6 | 71.7 | 77.6 | 91.5 | 19 |
|  |  | 9 | 43.1 | 64 | 72.6 | 77.6 | 86.3 | 19 |
|  |  | 12 | 50 | 60.83 | 69.75 | 74.03 | 78.1 | 16 |
| naive Th2 CD3+CD4+CD194+CD294+CD45RO- relative amount of CD3+CD4+CD194+CD294+ [%] | pCR | 0 | 7.81 | 17.30 | 30.30 | 44.20 | 54.80 | 11 |
|  |  | 3 | 5.77 | 8.98 | 22.30 | 40.95 | 63.60 | 9 |
|  |  | 6 | 3.63 | 4.26 | 11.75 | 23.10 | 33.30 | 10 |
|  |  | 9 | 2.56 | 8.00 | 12.30 | 19.20 | 26.30 | 11 |
|  |  | 12 | 2.11 | 4.33 | 11.60 | 14.50 | 30.50 | 9 |
|  | non-pCR | 0 | 6.07 | 14.1 | 18.4 | 27.65 | 43.8 | 18 |
|  |  | 3 | 0 | 5.79 | 12.7 | 22.4 | 65.5 | 17 |
|  |  | 6 | 2.47 | 6.15 | 14 | 26.5 | 63.1 | 19 |
|  |  | 9 | 3.8 | 5.25 | 8.02 | 12.3 | 54 | 19 |
|  |  | 12 | 1.76 | 4.368 | 11.25 | 17.08 | 44.8 | 16 |
| memory Th2 CD3+CD4+CD194+CD294+CD45RO+ relative amount of CD3+CD4+CD194+CD294+ [%] | pCR | 0 | 45.20 | 55.80 | 69.70 | 82.70 | 92.20 | 11 |
|  |  | 3 | 36.40 | 59.05 | 77.70 | 91.00 | 94.20 | 9 |
|  |  | 6 | 66.70 | 76.90 | 88.25 | 95.78 | 96.40 | 10 |
|  |  | 9 | 73.70 | 80.80 | 87.70 | 92.00 | 97.40 | 11 |
|  |  | 12 | 69.50 | 85.50 | 88.40 | 95.65 | 97.90 | 9 |
|  | non-pCR | 0 | 56.2 | 72.35 | 81.6 | 85.9 | 93.9 | 18 |
|  |  | 3 | 34.5 | 77.6 | 87.3 | 92.9 | 99.1 | 17 |
|  |  | 6 | 36.9 | 73.5 | 86 | 93.8 | 97.5 | 19 |
|  |  | 9 | 46 | 87.7 | 92 | 94.7 | 96.2 | 19 |
|  |  | 12 | 55.2 | 82.93 | 88.75 | 95.65 | 98.2 | 16 |

**Table S5 (continued).** Descriptive statistics of flow cytometry data (outcome)

| cell  population | group | time of  assessment | minimum | | Q1 | median | Q3 | | maximum | | n |
| --- | --- | --- | --- | --- | --- | --- | --- | --- | --- | --- | --- |
| Th1 / Th2 ratio | pCR | 0 | 0.01 | | 1.21 | 1.38 | 3.79 | | 5.41 | | 11 |
|  |  | 3 | 0.00 | | 0.96 | 1.53 | 2.83 | | 6.93 | | 9 |
|  |  | 6 | 0.00 | | 0.52 | 1.86 | 3.44 | | 4.39 | | 10 |
|  |  | 9 | 0.00 | | 0.44 | 1.21 | 2.42 | | 3.87 | | 11 |
|  |  | 12 | 0.00 | | 0.41 | 0.96 | 2.23 | | 3.06 | | 9 |
|  | non-pCR | 0 | 0.2759 | | 0.71 | 0.9663 | 2.149 | | 6.2 | | 18 |
|  |  | 3 | 0.0302 | | 0.463 | 1.214 | 4.294 | | 7.864 | | 17 |
|  |  | 6 | 0.09481 | | 0.617 | 1.594 | 2.691 | | 4.667 | | 19 |
|  |  | 9 | 0.08865 | | 0.45 | 0.7213 | 1.641 | | 4.947 | | 19 |
|  |  | 12 | 0.02786 | | 0.186 | 0.6582 | 1.703 | | 12.69 | | 16 |
| Th / Treg ratio | pCR | 0 | 2.99 | 6.56 | | 14.64 | | 21.32 | | 36.18 | 11 |
|  |  | 3 | 2.05 | 7.155 | | 10.82 | | 14.6 | | 16.49 | 8 |
|  |  | 6 | 5.21 | 6.005 | | 9.16 | | 13.38 | | 16.88 | 10 |
|  |  | 9 | 3.96 | 8.508 | | 10.49 | | 13.08 | | 14.58 | 10 |
|  |  | 12 | 2.54 | 4.34 | | 7.28 | | 13.6 | | 21.65 | 9 |
|  | non-pCR | 0 | 1.18 | 7.48 | | 12.21 | | 17.5 | | 47.47 | 17 |
|  |  | 3 | 2.14 | 7.93 | | 11.9 | | 14.65 | | 36.48 | 17 |
|  |  | 6 | 1.18 | 5.64 | | 8.8 | | 13.88 | | 38.44 | 19 |
|  |  | 9 | 1.8 | 5.89 | | 8.515 | | 11.92 | | 22.95 | 18 |
|  |  | 12 | 1.5 | 5.99 | | 8.52 | | 12.59 | | 28.46 | 15 |
| CTL / Treg ratio | pCR | 0 | 0.66 | 1.25 | | 2 | | 3.75 | | 9.83 | 11 |
|  |  | 3 | 1.26 | 1.583 | | 2.185 | | 6.418 | | 29.57 | 8 |
|  |  | 6 | 1.61 | 1.645 | | 2.805 | | 4.718 | | 8.73 | 10 |
|  |  | 9 | 1.12 | 1.513 | | 2.5 | | 4.583 | | 10.75 | 10 |
|  |  | 12 | 2.07 | 3.02 | | 4.82 | | 6.965 | | 9.57 | 9 |
|  | non-pCR | 0 | 0.51 | 0.945 | | 2.46 | | 3.64 | | 17.18 | 17 |
|  |  | 3 | 0.37 | 1.66 | | 2.11 | | 4.31 | | 14.92 | 17 |
|  |  | 6 | 0.53 | 1.82 | | 4.05 | | 5.97 | | 13.26 | 19 |
|  |  | 9 | 0.6 | 2.283 | | 4.155 | | 6.173 | | 16.11 | 18 |
|  |  | 12 | 0.77 | 4.75 | | 5.37 | | 7.18 | | 21.19 | 15 |

**Table S6.** Descriptive statistics of multiplex immunoassay data (outcome)

| cytokine | group | time of  assessment | minimum | Q1 | median | Q3 | maximum | n |
| --- | --- | --- | --- | --- | --- | --- | --- | --- |
| IL-4 [pg/ml] | pCR | 0 | 16.37 | 16.8 | 31.56 | 445 | 1328 | 11 |
|  |  | 3 | 7.15 | 17.6 | 27.33 | 382 | 845.6 | 11 |
|  |  | 6 | 4.79 | 19.9 | 51.99 | 374 | 1580 | 11 |
|  |  | 9 | 4.52 | 12.2 | 28.07 | 388 | 1672 | 12 |
|  |  | 12 | 8.57 | 20.2 | 23.39 | 220 | 1666 | 11 |
|  | non-pCR | 0 | 12.04 | 20.2 | 37.85 | 88 | 784.8 | 21 |
|  |  | 3 | 5.93 | 16.6 | 35.71 | 94.5 | 530.3 | 18 |
|  |  | 6 | 9.28 | 23.6 | 31.44 | 78.2 | 818.2 | 17 |
|  |  | 9 | 9.2 | 24.4 | 73.14 | 507 | 1381 | 19 |
|  |  | 12 | 10.07 | 24.5 | 30.01 | 131 | 1715 | 18 |
| IL-7 [pg/ml] | pCR | 0 | 1.94 | 4.45 | 6.5 | 9.64 | 18.6 | 11 |
|  |  | 3 | 2.24 | 4.72 | 6.23 | 12.3 | 17.51 | 11 |
|  |  | 6 | 2.07 | 3.59 | 6.27 | 12 | 15.91 | 11 |
|  |  | 9 | 1.15 | 3.27 | 6.015 | 10.3 | 15.74 | 12 |
|  |  | 12 | 1.87 | 3.96 | 6.73 | 11.2 | 20.08 | 11 |
|  | non-pCR | 0 | 2.98 | 6.32 | 10.22 | 12.9 | 16.64 | 21 |
|  |  | 3 | 2.72 | 5.27 | 7.65 | 9.57 | 15.39 | 18 |
|  |  | 6 | 3.03 | 5.13 | 6.66 | 10.5 | 15.27 | 17 |
|  |  | 9 | 3.2 | 4.84 | 7.91 | 10.3 | 14.94 | 19 |
|  |  | 12 | 2.72 | 5.21 | 8.24 | 8.82 | 16.67 | 18 |
| IL-8 [pg/ml] | pCR | 0 | 0.13 | 0.86 | 3.89 | 37.5 | 80.82 | 11 |
|  |  | 3 | 0.13 | 2.55 | 4.45 | 25.7 | 73.67 | 11 |
|  |  | 6 | 0.13 | 1.98 | 4.05 | 39.8 | 85.84 | 11 |
|  |  | 9 | 0.13 | 1.15 | 3.43 | 57.6 | 130.9 | 12 |
|  |  | 12 | 1.11 | 1.89 | 3.07 | 34.6 | 121.7 | 11 |
|  | non-pCR | 0 | 1.1 | 2.55 | 3.17 | 7.33 | 71.1 | 21 |
|  |  | 3 | 2.33 | 2.96 | 4.315 | 10.1 | 34.55 | 18 |
|  |  | 6 | 1.5 | 3.01 | 3.78 | 9.23 | 118.1 | 17 |
|  |  | 9 | 1.85 | 3.1 | 5.24 | 22.9 | 157.9 | 19 |
|  |  | 12 | 1.03 | 2.75 | 4.595 | 13.7 | 189.4 | 18 |
| IL-10 [pg/ml] | pCR | 0 | 4.42 | 7.47 | 11.07 | 113 | 222.9 | 11 |
|  |  | 3 | 3.75 | 6.83 | 12.66 | 112 | 144.2 | 11 |
|  |  | 6 | 3.03 | 8.51 | 24.64 | 104 | 262 | 11 |
|  |  | 9 | 3.06 | 4.33 | 21.22 | 101 | 274.7 | 12 |
|  |  | 12 | 5.14 | 7.89 | 14.13 | 40.6 | 242.8 | 11 |
|  | non-pCR | 0 | 5.18 | 16.3 | 25.61 | 43.7 | 193.6 | 21 |
|  |  | 3 | 4.3 | 12.2 | 28.66 | 41.5 | 100.7 | 18 |
|  |  | 6 | 6.1 | 11.8 | 25.56 | 34.1 | 159.7 | 17 |
|  |  | 9 | 4.93 | 17.8 | 23.59 | 91.9 | 221.7 | 19 |
|  |  | 12 | 5.49 | 12.3 | 18.16 | 34.3 | 195.1 | 18 |

**Table S6 (continued).** Descriptive statistics of multiplex immunoassay data (outcome)

| cytokine | group | time of  assessment | minimum | Q1 | median | Q3 | maximum | n |
| --- | --- | --- | --- | --- | --- | --- | --- | --- |
| IL-12(p70) [pg/ml] | pCR | 0 | 1.22 | 2.6 | 4.39 | 8.19 | 16.11 | 11 |
|  |  | 3 | 1.36 | 2.42 | 2.9 | 8.59 | 11.16 | 11 |
|  |  | 6 | 1.07 | 1.92 | 4.44 | 6.61 | 8.39 | 11 |
|  |  | 9 | 0.38 | 1.55 | 3.815 | 6.13 | 9.42 | 12 |
|  |  | 12 | 0.9 | 1.53 | 4.3 | 7.76 | 31.33 | 11 |
|  | non-pCR | 0 | 2.11 | 3.21 | 5.04 | 10.3 | 16.97 | 21 |
|  |  | 3 | 1.64 | 2.76 | 4.975 | 7.52 | 11.03 | 18 |
|  |  | 6 | 1.42 | 3.16 | 4.81 | 6.55 | 7.68 | 17 |
|  |  | 9 | 1.23 | 2.94 | 4.95 | 7.87 | 11.63 | 19 |
|  |  | 12 | 2.65 | 3.34 | 3.97 | 5.4 | 14.15 | 18 |
| IFN-γ [pg/ml] | pCR | 0 | 4.96 | 11.9 | 17.7 | 34.1 | 71.32 | 11 |
|  |  | 3 | 3.59 | 7.02 | 17.28 | 47 | 50.67 | 11 |
|  |  | 6 | 4.26 | 6.24 | 25.61 | 27.8 | 44.41 | 11 |
|  |  | 9 | 2.01 | 6.94 | 20.29 | 39.2 | 45.89 | 12 |
|  |  | 12 | 3.46 | 9.08 | 19.63 | 33.4 | 44.53 | 11 |
|  | non-pCR | 0 | 7.91 | 14.5 | 23.01 | 37.4 | 74.54 | 21 |
|  |  | 3 | 4.9 | 10.9 | 17.75 | 22.8 | 57.28 | 18 |
|  |  | 6 | 6.83 | 11.8 | 15.45 | 29.3 | 48.36 | 17 |
|  |  | 9 | 4.57 | 14.9 | 20.44 | 30.8 | 49.34 | 19 |
|  |  | 12 | 2.79 | 12 | 18.45 | 27.1 | 46.85 | 18 |
| TNF [pg/ml] | pCR | 0 | 3.37 | 4.87 | 6.82 | 9.85 | 14.53 | 11 |
|  |  | 3 | 3.2 | 5.65 | 7.43 | 10.4 | 22.42 | 11 |
|  |  | 6 | 2.83 | 3.67 | 7.97 | 13.7 | 14.55 | 11 |
|  |  | 9 | 2.25 | 4.14 | 6.31 | 9.83 | 20.36 | 12 |
|  |  | 12 | 2.43 | 5.33 | 6.88 | 11.3 | 18.9 | 11 |
|  | non-pCR | 0 | 2.86 | 7.16 | 8.39 | 11.9 | 16.55 | 21 |
|  |  | 3 | 6.95 | 8.18 | 10.11 | 11.9 | 17.8 | 18 |
|  |  | 6 | 3.38 | 7.26 | 9.31 | 11.3 | 14.71 | 17 |
|  |  | 9 | 4.69 | 6.97 | 8.28 | 12.2 | 14.26 | 19 |
|  |  | 12 | 4.72 | 7.54 | 8.89 | 10.8 | 14.69 | 18 |


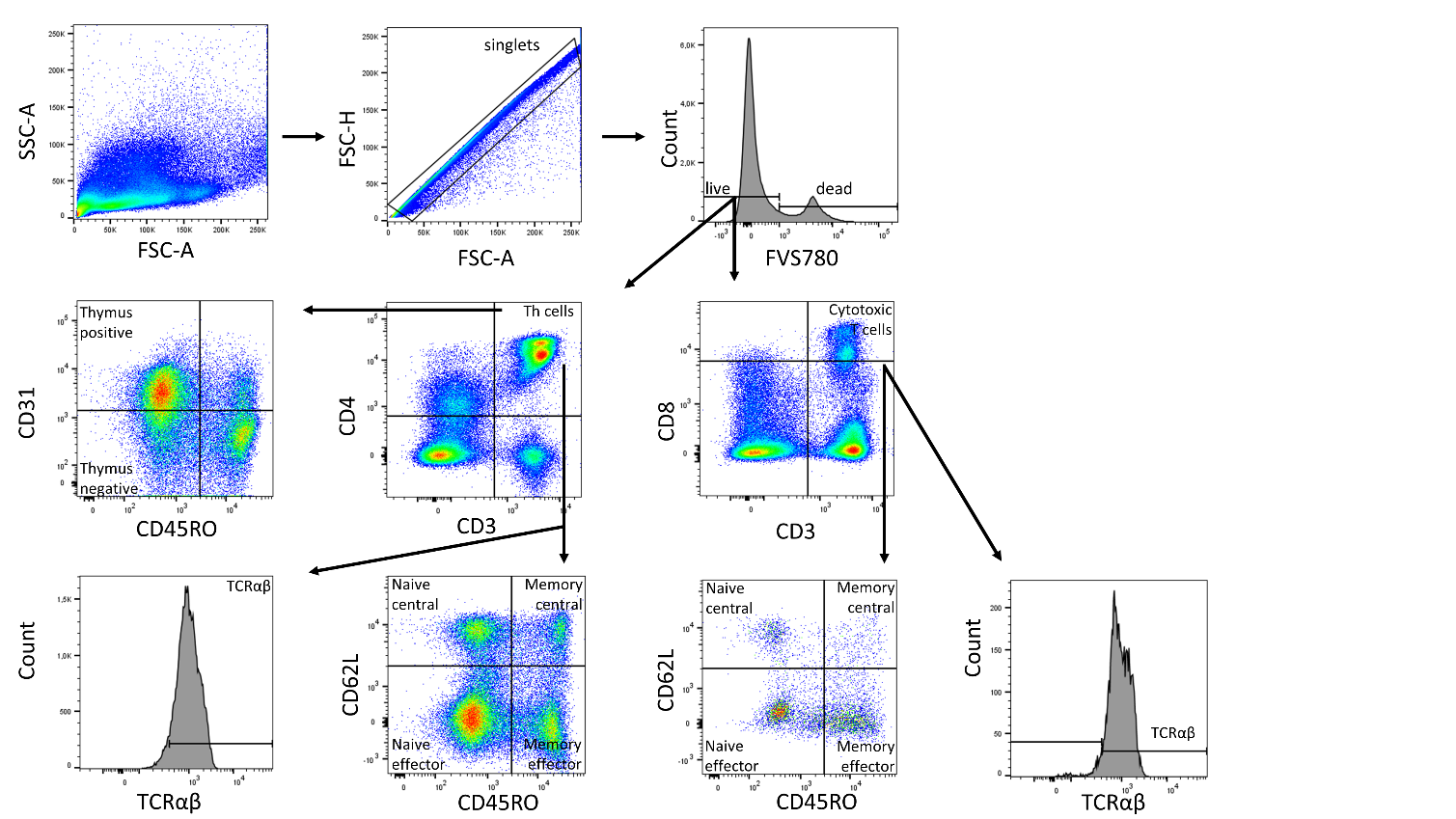


**Figure S1.** Gating strategy of T cell subpopulations (panel 1). Gating strategy of T cell subpopulations (panel 2) of a representative PBMC sample from a BEGYN-1 study participant. Analysis was performed using FlowJo (BD Biosciences). Gating was based first on morphological parameters (FSC-H/FSC-A) and FVS780 negative cells were considered as viable cells. Within the viable cells, the number of CD3+ cells was determined. The CD3+ fraction was divided into CD4+ T helper cells and CD8+ cytotoxic T cells. Both cell fractions were subdivided into naïve central (CD62L+CD45RO−), naïve effector (CD62L−CD45RO−), memory effector (CD62L−CD45RO+), and memory central (CD62L+CD45RO+) as well as TCRαβ, and Th or CTL with TCRαβ. CD4+ Th cells were further subdivided into naïve thymus-negative (CD45RO−CD31−) and positive (CD45RO−CD31+) Th cells.


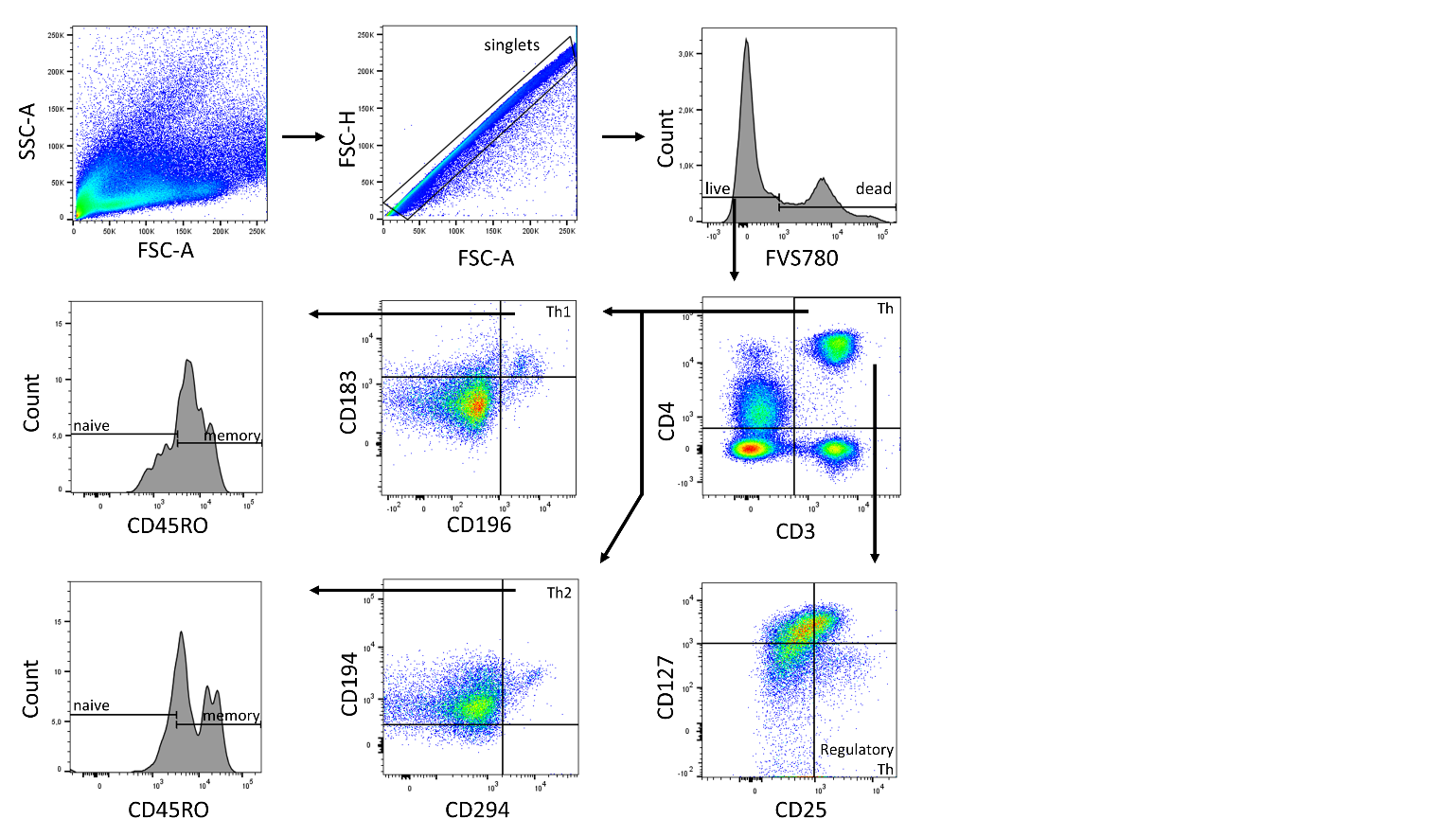


**Figure S2.** Gating strategy of T cell subpopulations (panel 2). Gating strategy of T cell subpopulations (panel 2) of a representative PBMC sample from a BEGYN-1 study participant. Analysis was performed using FlowJo (BD Biosciences). Gating was based first on morphological parameters (FSC-H/FSC-A) and FVS780 negative cells were considered as viable cells. Within the viable cells, the number of CD3+ cells was determined. CD4+ T helper cells were identified as well as Th1 cells (CD183+CD196+) and Th2 cells (CD194+CD294+) followed by their subpopulations naïve (CD45RO-) and memory (CD45RO+). Regulatory Th cells were identified as CD25+CD127-.


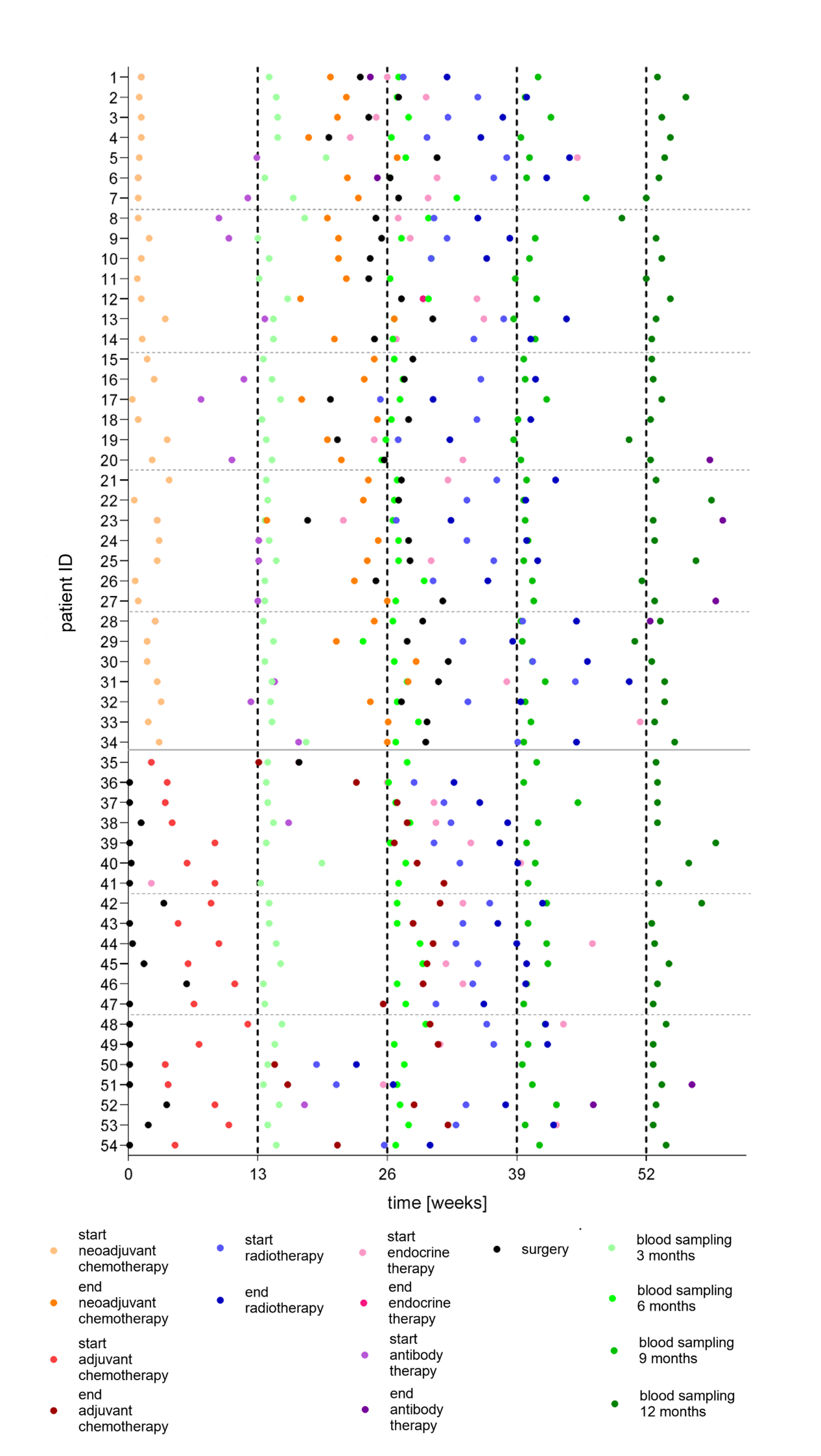


**Figure S3: Individual treatment patterns of CHT patients.** The individual therapy interventions (light orange: start of neoadjuvant chemotherapy; dark orange: end of neoadjuvant chemotherapy; light red: start of adjuvant chemotherapy, dark red: end of adjuvant chemotherapy; light blue: start of radiotherapy; dark blue: end of radiotherapy; black: surgery; light pink: start of endocrine therapy; dark pink: end of endocrine therapy; light purple: start of antibody therapy; dark purple: end of antibody therapy) and blood samples for the BEGYN-1 study (first blood sample/baseline measurement at 0 weeks, green gradient from light to dark: blood samples after 3, 6, 9 and 12 months) are shown as a function of time. The basis for this is provided by guidelines, which result in a scheme for the treatment of breast cancer, as shown in Figure 1.


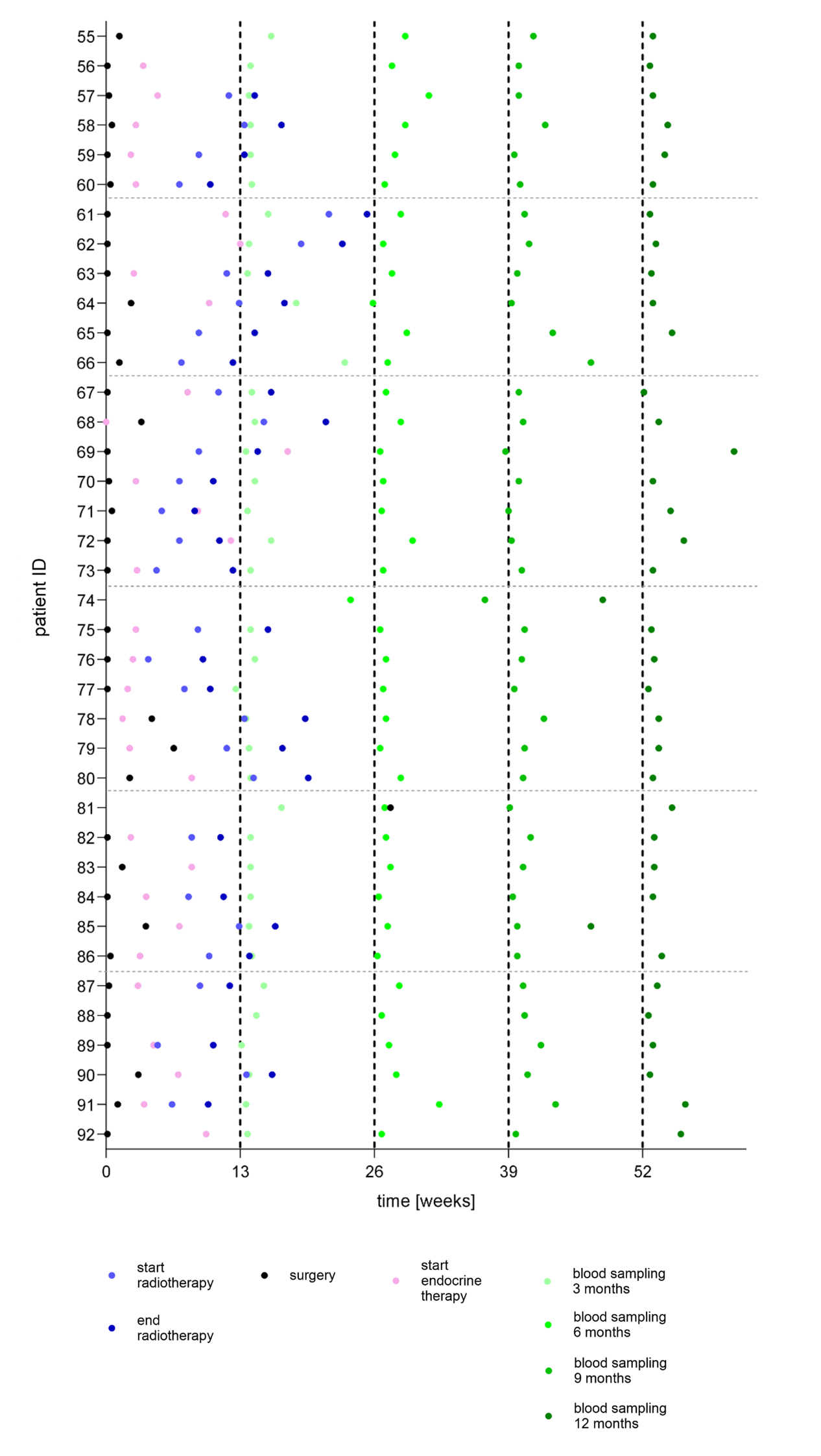


**Figure S4: Individual treatment patterns of NCHT patients.** The individual therapy interventions (light blue: start of radiotherapy; dark blue: end of radiotherapy; black: surgery; purple: start of endocrine therapy) and blood samples for the BEGYN-1 study (first blood sample/baseline measurement at 0 weeks, green gradient from light to dark: blood samples after 3, 6, 9 and 12 months) are shown as a function of time. The basis for this is provided by guidelines, which result in a scheme for the treatment of breast cancer, as shown in Figure 1.


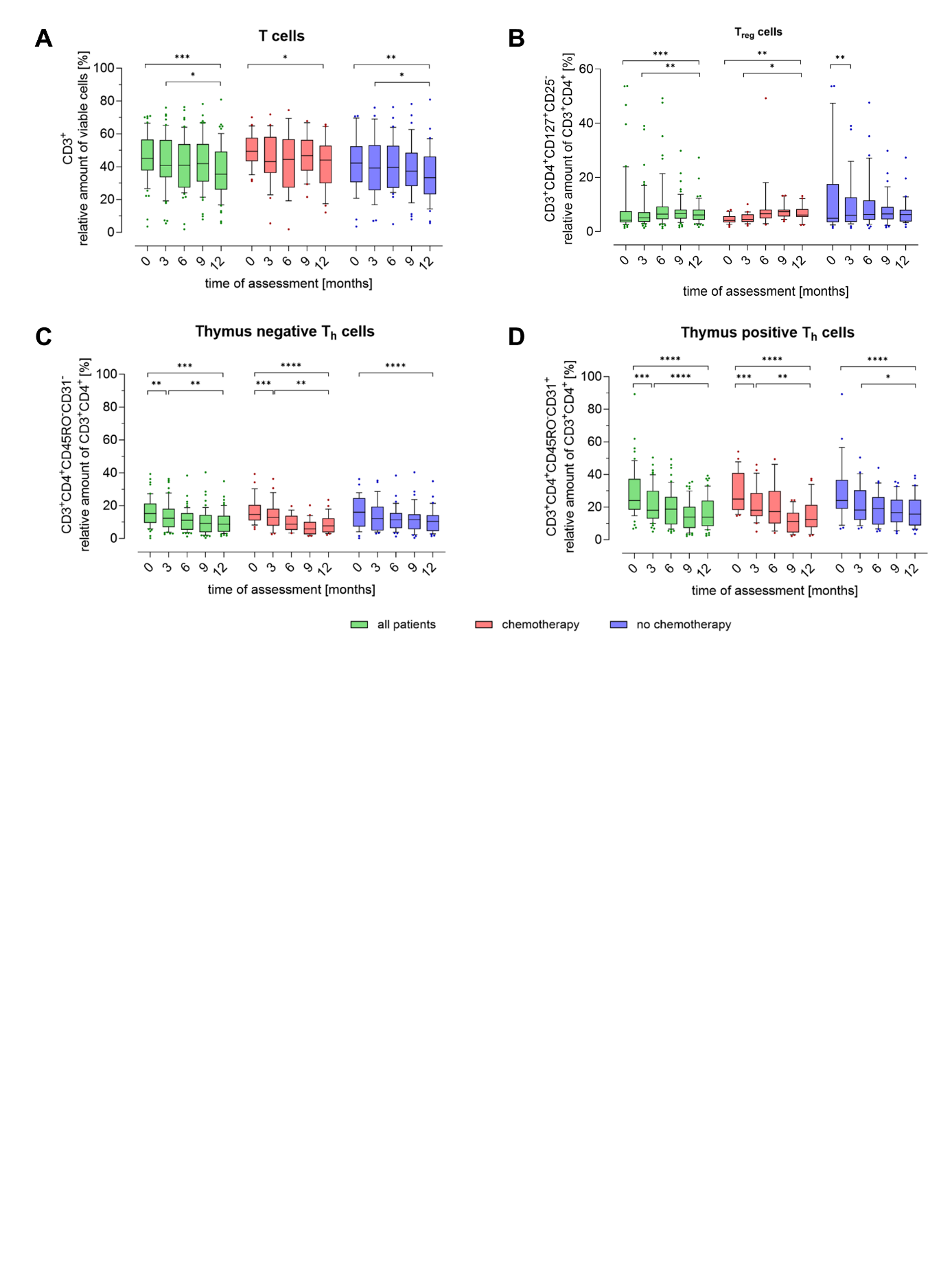


**Figure S5. Peripheral blood T cell populations in breast cancer patients receiving chemotherapy without potentially immunomodulatory therapy during one year.** Patients receiving anti-HER2 agents, Atezolizumab, Cepecitabine or Abemaciclib were excluded in comparison to Figure 2, which results in n(CHT)=25, n(NCHT)=38 and n(all)=61. The proportion of (**A**) T cells (CD3+), (**B**) regulatory T cells (Treg CD3+CD4+CD25+CD127-), (**C**) thymus negative T cells (CD3+CD4+CD45RO-CD31-), (**D**) thymus positive T cells (CD3+CD4+CD45RO-CD31+) in relation to the parent cell population was determined quarterly by flow cytometry. The first measurement (baseline, 0 months) was performed after diagnosis and before initiation of therapy. Boxes extend from the 10^th^ to the 90^th^ percentiles. Points below and above the whiskers are drawn as individual data points. * p<0.05, ** p < 0.01, *** p < 0.005, **** p < 0.001, therapy group differences were assessed using two-tailed Mann Whitney U test, differences over time within one group were assessed using two-tailed Wilcoxon matched-paired signed rank test.


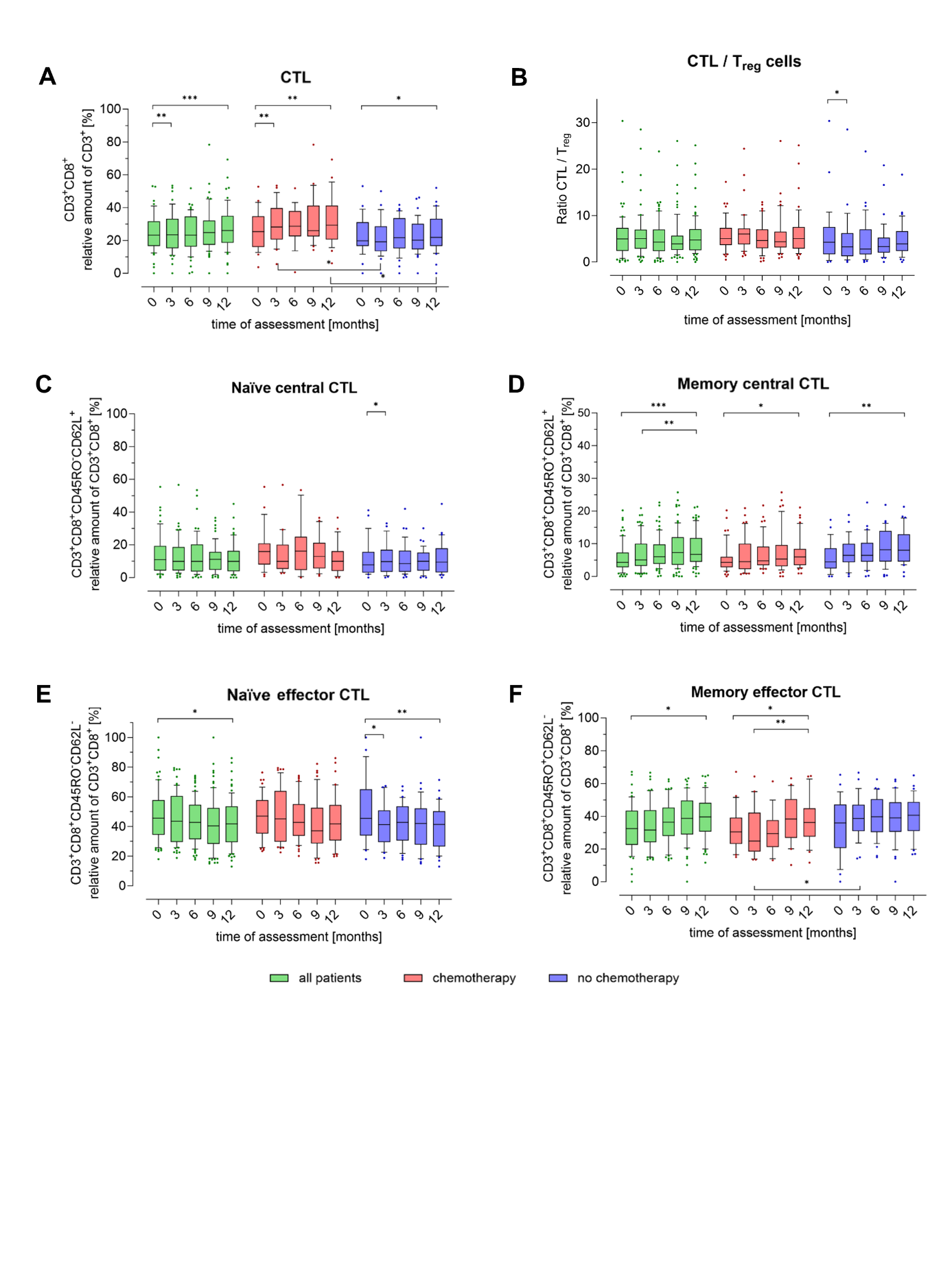


**Figure S6. Peripheral blood subpopulations of cytotoxic T cells in breast cancer patients receiving chemotherapy without potentially immunomodulatory therapy during one year.** Patients receiving anti-HER2 agents, Atezolizumab, Cepecitabine or Abemaciclib were excluded in comparison to Figure 3, which results in n(CHT)=25, n(NCHT)=38 and n(all)=61. The proportion of T cell subpopulations (**A**) cytotoxic T cells (CTL, CD3+CD8+), (**B**) shows CTL/Treg ratio over time (**C**) naïve central CTL (CD3+CD8+CD45RO-CD62L+), (**D**) memory central CTL (CD3+CD8+CD45RO+CD62L+), (**E**) naïve effector CTL (CD3+CD8+CD45RO+CD62L-) and (**F**) memory effector CTL (CD3+CD8+CD45RO+CD62L-) in relation to the parent cell population was determined quarterly by flow cytometry. The first measurement (baseline, 0 months) was performed after diagnosis and before initiation of therapy. Boxes extend from the 10^th^ to the 90^th^ percentiles. Points below and above the whiskers are drawn as individual data points. * p<0.05, ** p < 0.01, *** p < 0.005, **** p < 0.001, therapy group differences were assessed using two-tailed Mann Whitney U test, differences over time within one group were assessed using two-tailed Wilcoxon matched-paired signed rank test.


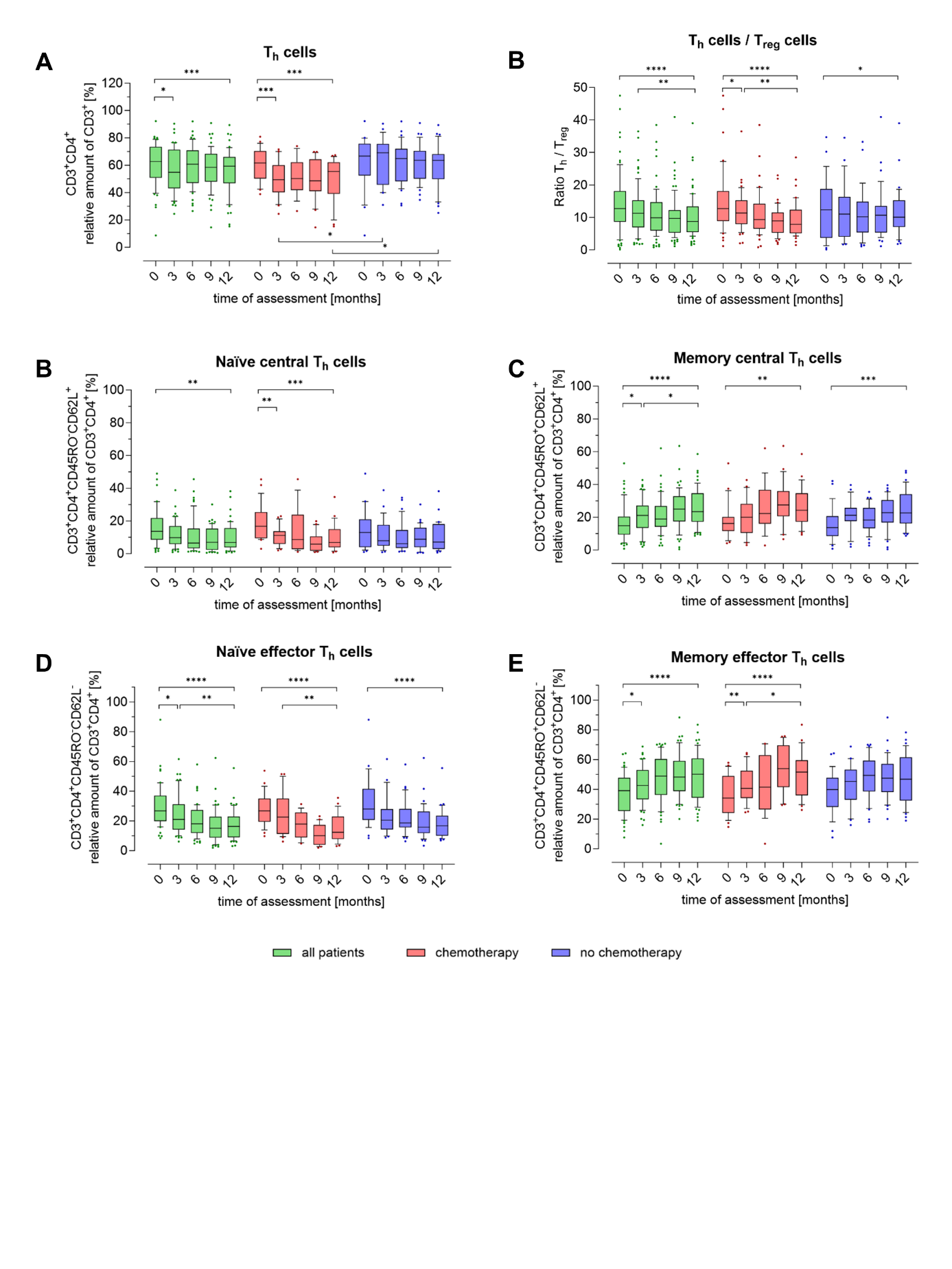


**Figure S7. Peripheral blood subpopulations of T helper cells in breast cancer patients receiving chemotherapy without potentially immunomodulatory therapy during one year.** Patients receiving anti-HER2 agents, Atezolizumab, Cepecitabine or Abemaciclib were excluded in comparison to Figure 4, which results in n(CHT)=25, n(NCHT)=38 and n(all)=61. The proportion of T cell subpopulations (**A**) T helper cells (Th, CD3+CD4+), (**B**) shows the Th/Treg ratio, (**C**) naïve central Th cells (CD3+CD4+CD45RO-CD62L+), (**D**) memory central Th cells (CD3+CD4+CD45RO+CD62L+), (**E**) naïve effector Th cells (CD3+CD4+CD45RO+CD62L-) and (**F**) memory effector Th cells (CD3+CD4+CD45RO+CD62L-) in relation to the parent cell population was determined quarterly by flow cytometry. The first measurement (baseline, 0 months) was performed after diagnosis and before initiation of therapy. Boxes extend from the 10^th^ to the 90^th^ percentiles. Points below and above the whiskers are drawn as individual data points. * p<0.05, ** p < 0.01, *** p < 0.005, **** p < 0.001, therapy group differences were assessed using two-tailed Mann Whitney U test, differences over time within one group were assessed using two-tailed Wilcoxon matched-paired signed rank test.


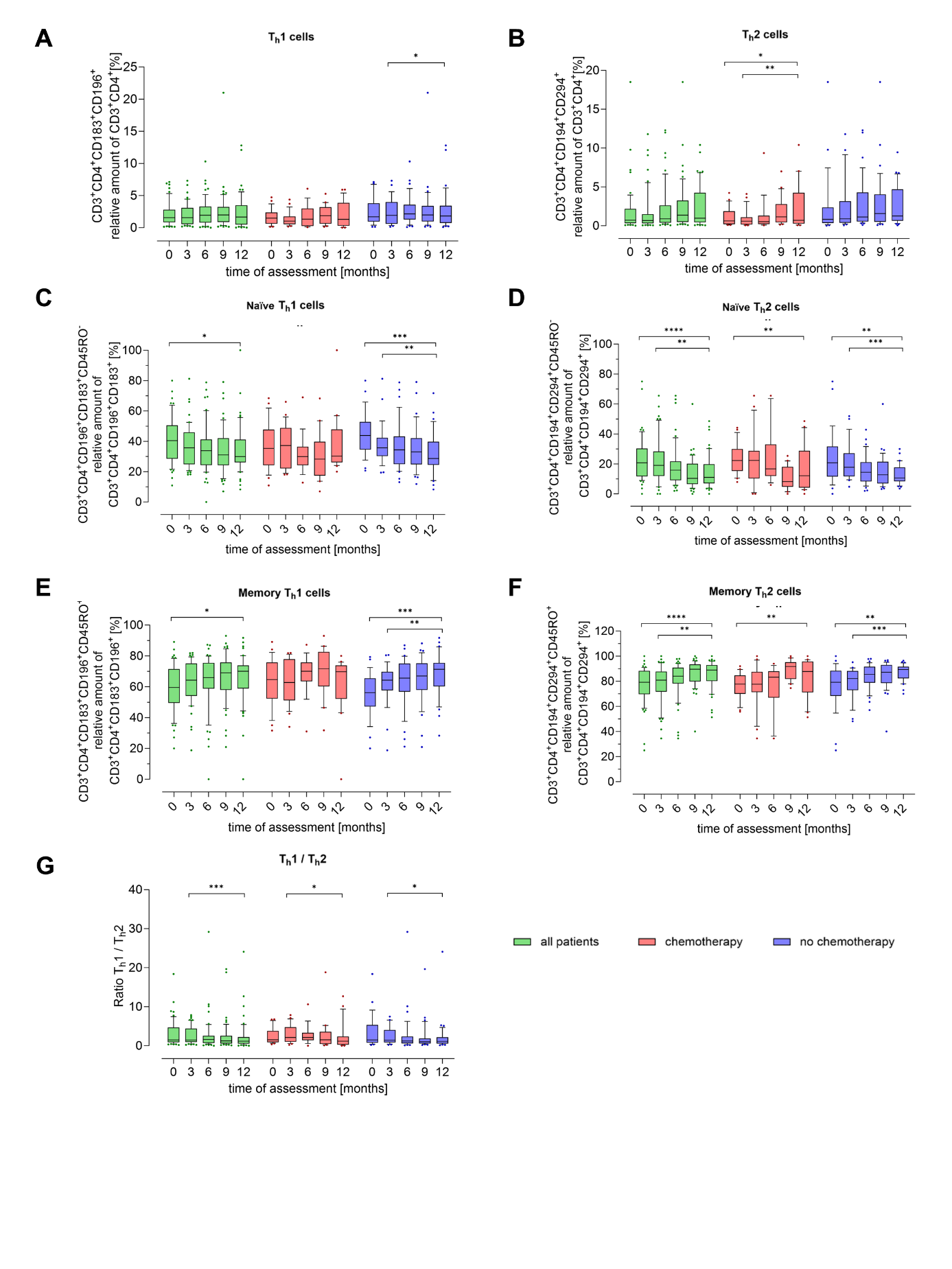


**Figure S8. Peripheral blood subpopulations of Th1 and Th2 cells in breast cancer patients receiving chemotherapy without potentially immunomodulatory therapy during one year.** Patients receiving anti-HER2 agents, Atezolizumab, Cepecitabine or Abemaciclib were excluded in comparison to Figure 5, which results in n(CHT)=25, n(NCHT)=38 and n(all)=61. The proportion of T cell subpopulations (**A**) T helper cells 1 (Th1, CD3+CD4+CD183+CD196+), (**B**) T helper cells 2 (Th2, CD3+CD4+CD194+CD294+), (**C**) naïve Th1 (CD3+CD4+CD183+CD196+CD45RO-), (**D**) naïve Th2 (CD3+CD4+CD194+CD294+CD45RO-), (**E**) memory Th1 (CD3+CD4+CD183+CD196+CD45RO+) and (**F**) memory Th2 (CD3+CD4+CD194+CD294+CD45RO+) in relation to the parent cell population was determined quarterly by flow cytometry. The first measurement (baseline, 0 months) was performed after diagnosis and before initiation of therapy. (**G**) shows the Th1/Th2 ratio over time. Boxes extend from the 10^th^ to the 90^th^ percentiles. Points below and above the whiskers are drawn as individual data points. * p<0.05, ** p < 0.01, *** p < 0.005, **** p < 0.001, therapy group differences were assessed using two-tailed Mann Whitney U test, differences over time within one group were assessed using two-tailed Wilcoxon matched-paired signed rank test.


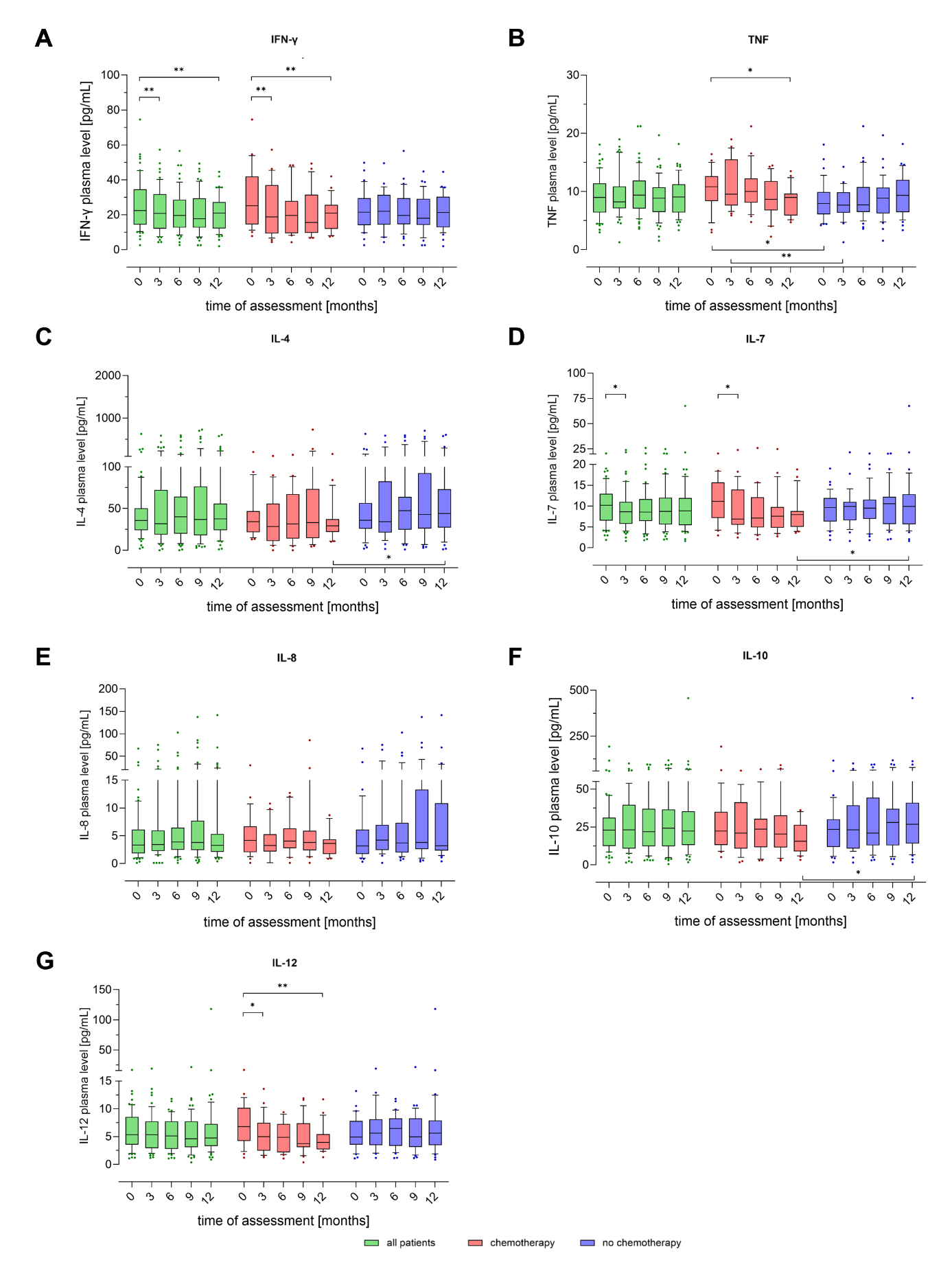


**Figure S9. Peripheral blood plasma cytokine levels in breast cancer patients receiving chemotherapy without potentially immunomodulatory therapy during one year.** Patients receiving anti-HER2 agents, Atezolizumab, Cepecitabine or Abemaciclib were excluded in comparison to Figure 6, which results in n(CHT)=25, n(NCHT)=38 and n(all)=61. The amount of (**A**) IFN-γ, (**B**) TNF, (**C**) IL-4, (**D**) IL-7, (**E**) IL-8, (**F**) IL-10 and (**G**) IL-12, was determined quarterly by multiplex cytokine assay (MAGPIX®). The first measurement (baseline, 0 months) was performed after diagnosis and before initiation of therapy. Boxes extend from the 10^th^ to the 90^th^ percentiles. Points below and above the whiskers are drawn as individual data points. * p<0.05, ** p < 0.01, *** p < 0.005, therapy group differences were assessed using two-tailed Mann Whitney U test, differences over time within one group were assessed using two-tailed Wilcoxon matched-paired signed rank test.
