# Supplementary figures and images for: Dynamics of T cell subpopulations and plasma cytokines during the first year of antineoplastic therapy in patients with breast cancer: the BEGYN-1 study
Source: Breast Cancer Res. 2025 Apr 1;27:50. doi: 10.1186/s13058-025-01997-9 (PMC11963634; doi:10.1186/s13058-025-01997-9)

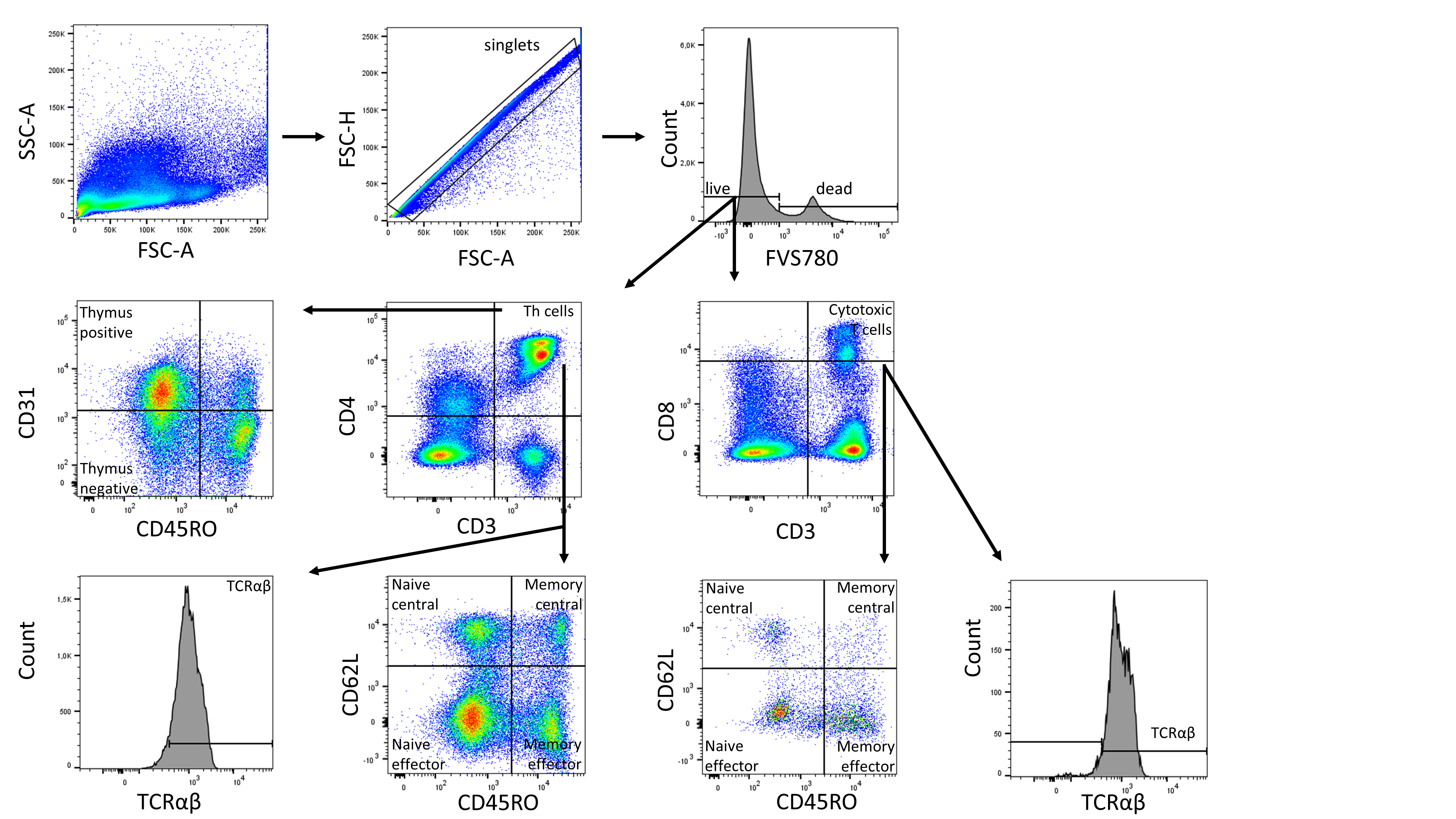

Supplement: Supplementary file 8 — Supplementary Material 8: Figure S1. Gating strategy of T cell subpopulations (panel 1). [file 13058_2025_1997_MOESM8_ESM.tif]

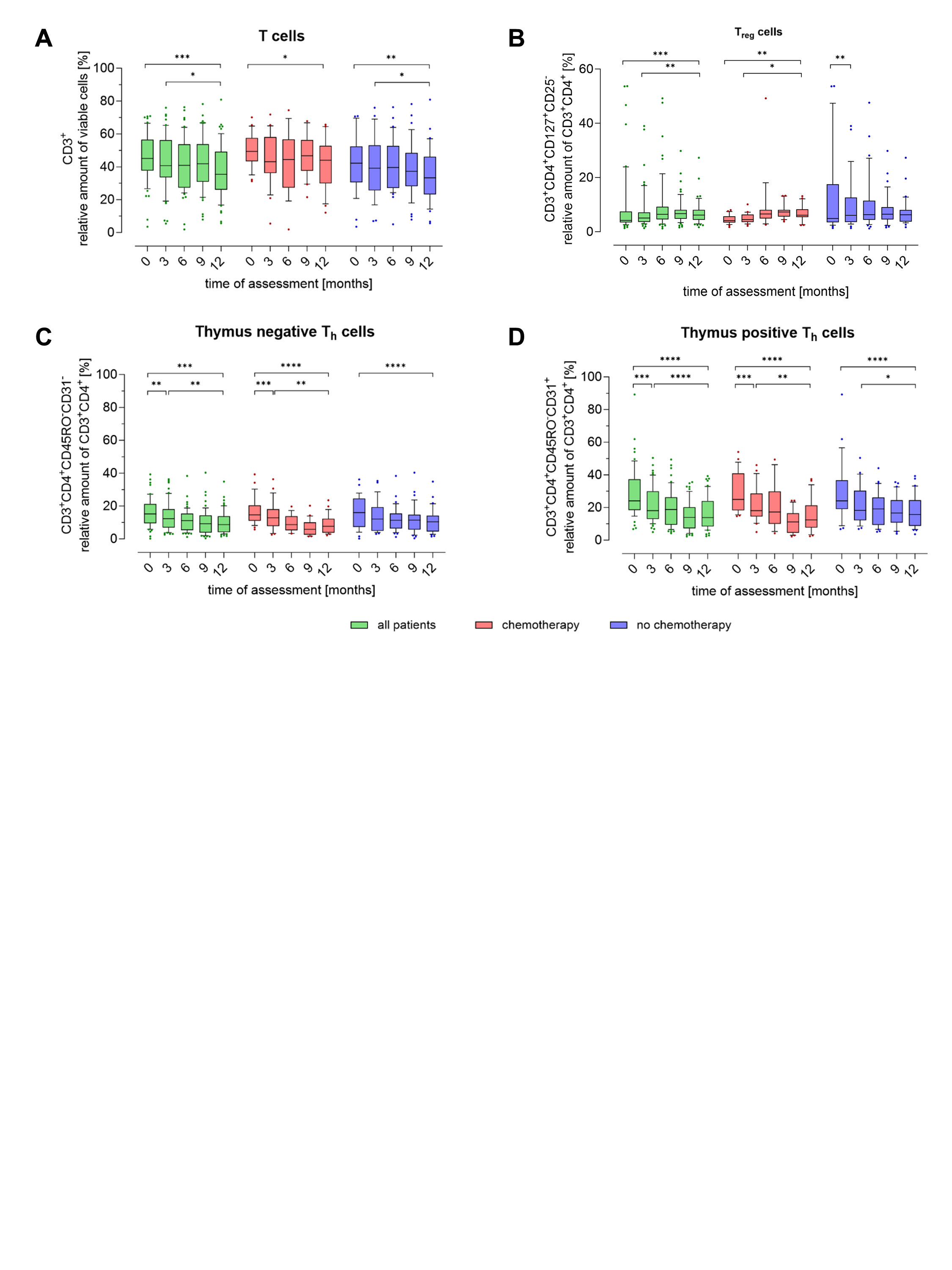

Supplement: Supplementary file 9 — Supplementary Material 9: Figure S2. Gating strategy of T cell subpopulations (panel 2). [file 13058_2025_1997_MOESM9_ESM.tif]

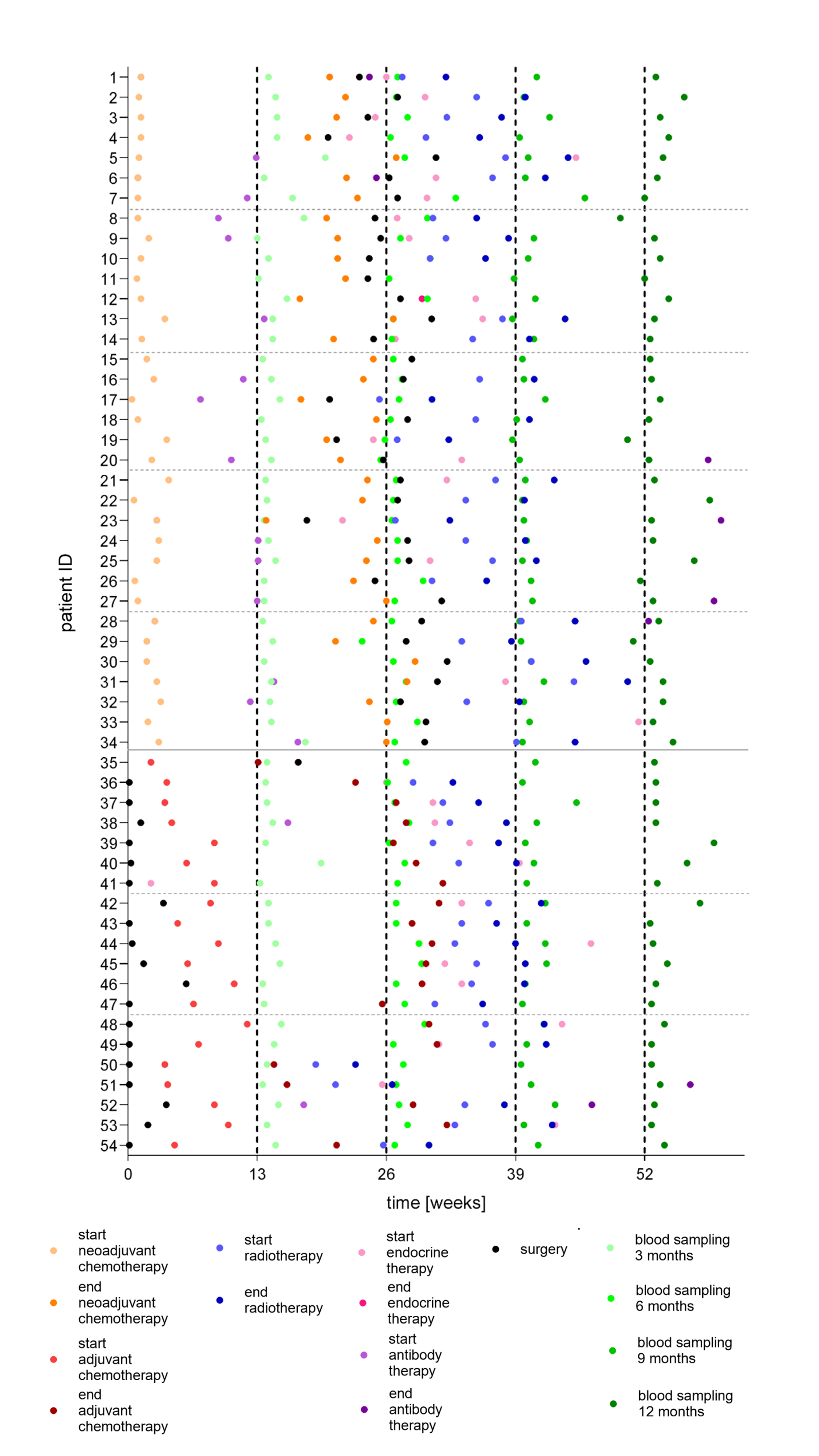

Supplement: Supplementary file 10 — Supplementary Material 10: Figure S3: Individual treatment patterns of CHT patients. [file 13058_2025_1997_MOESM10_ESM.tif]

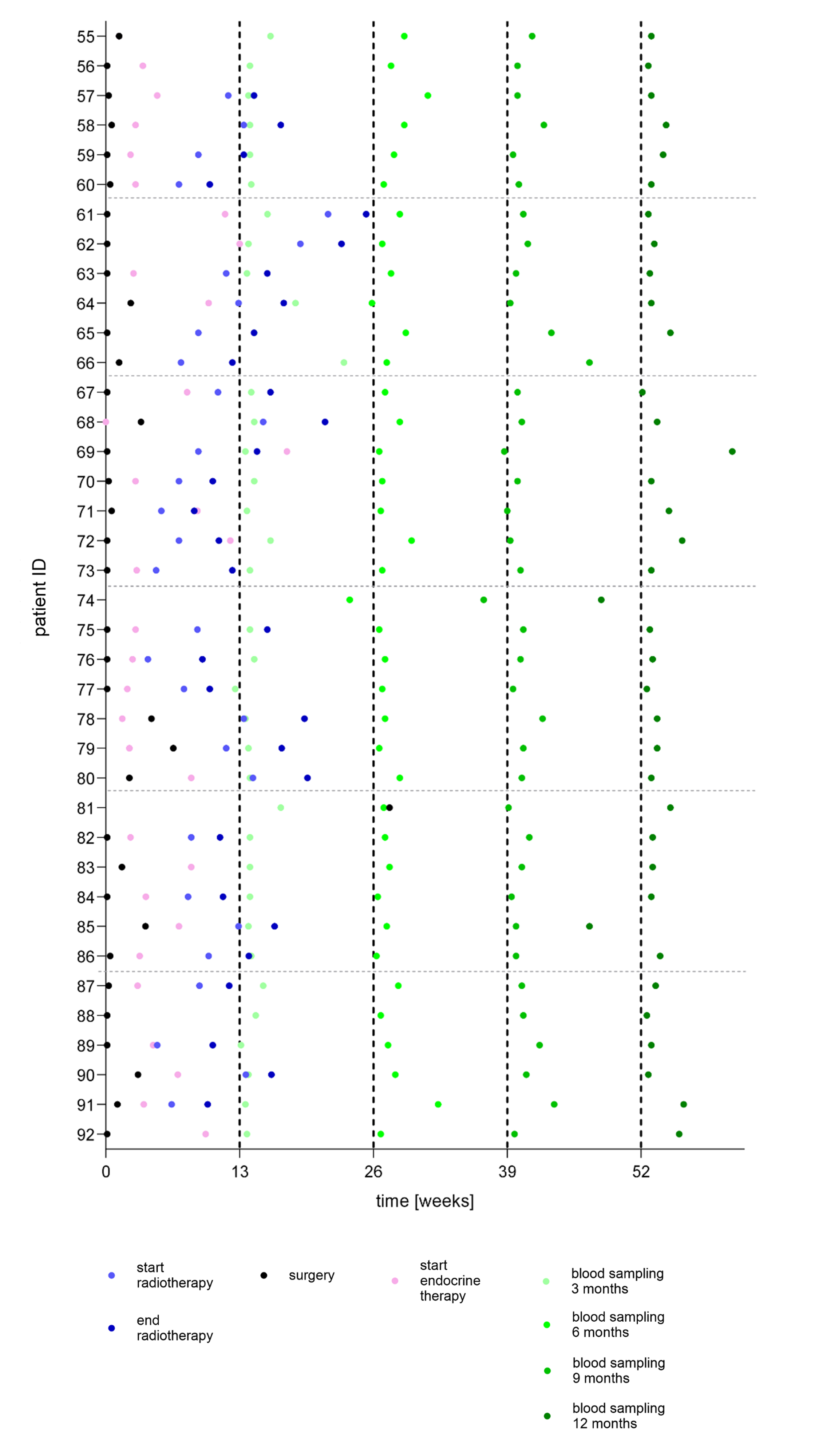

Supplement: Supplementary file 11 — Supplementary Material 11: Figure S4: Individual treatment patterns of NCHT patients. [file 13058_2025_1997_MOESM11_ESM.tif]

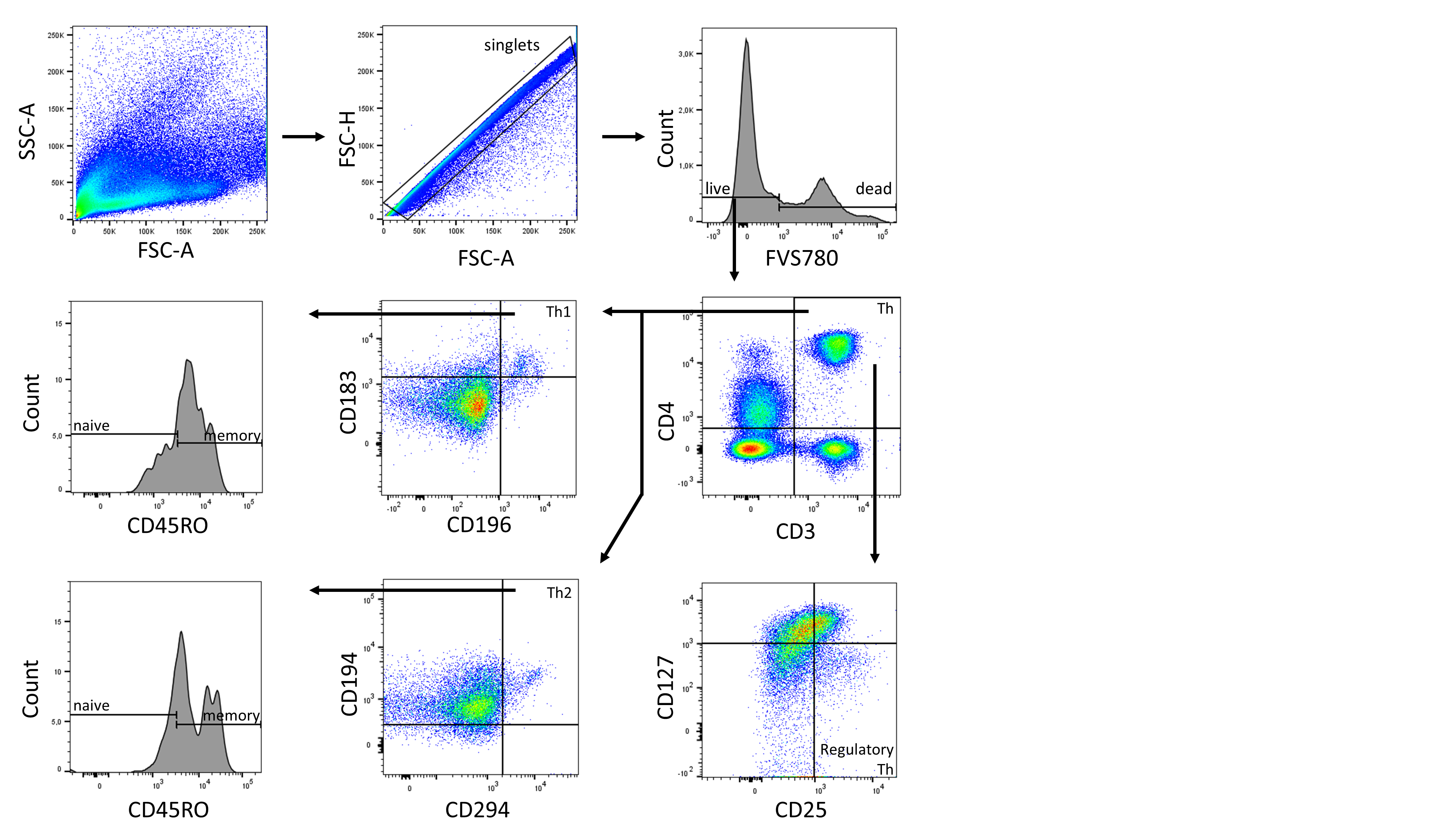

Supplement: Supplementary file 12 — Supplementary Material 12: Figure S5: Peripheral blood T cell populations in breast cancer patients receiving chemotherapy without potentially immunomodulatory therapy during one year. [file 13058_2025_1997_MOESM12_ESM.tif]

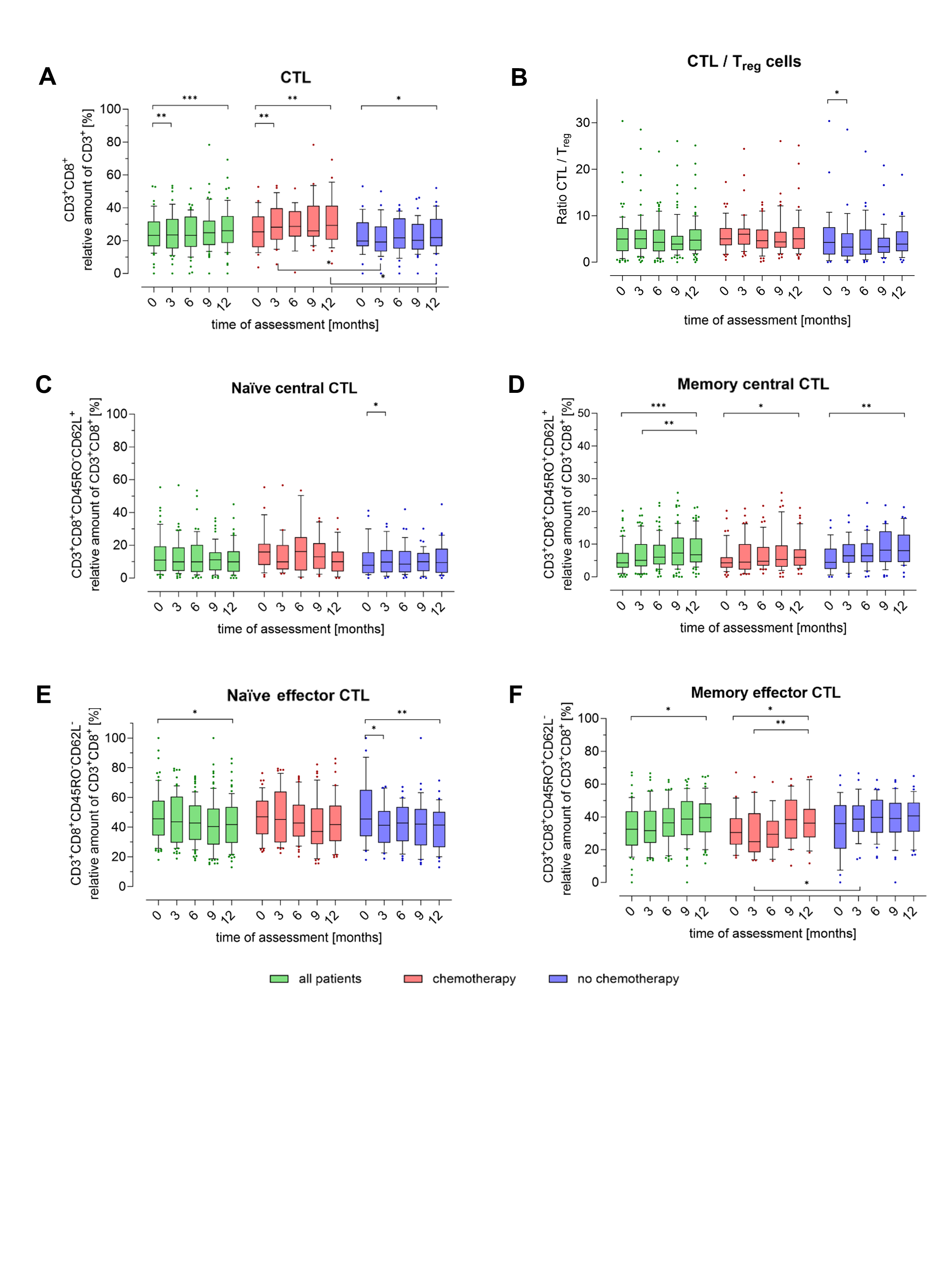

Supplement: Supplementary file 13 — Supplementary Material 13: Figure S6: Peripheral blood subpopulations of cytotoxic T cells in breast cancer patients receiving chemotherapy without potentially immunomodulatory therapy during one year [file 13058_2025_1997_MOESM13_ESM.tif]

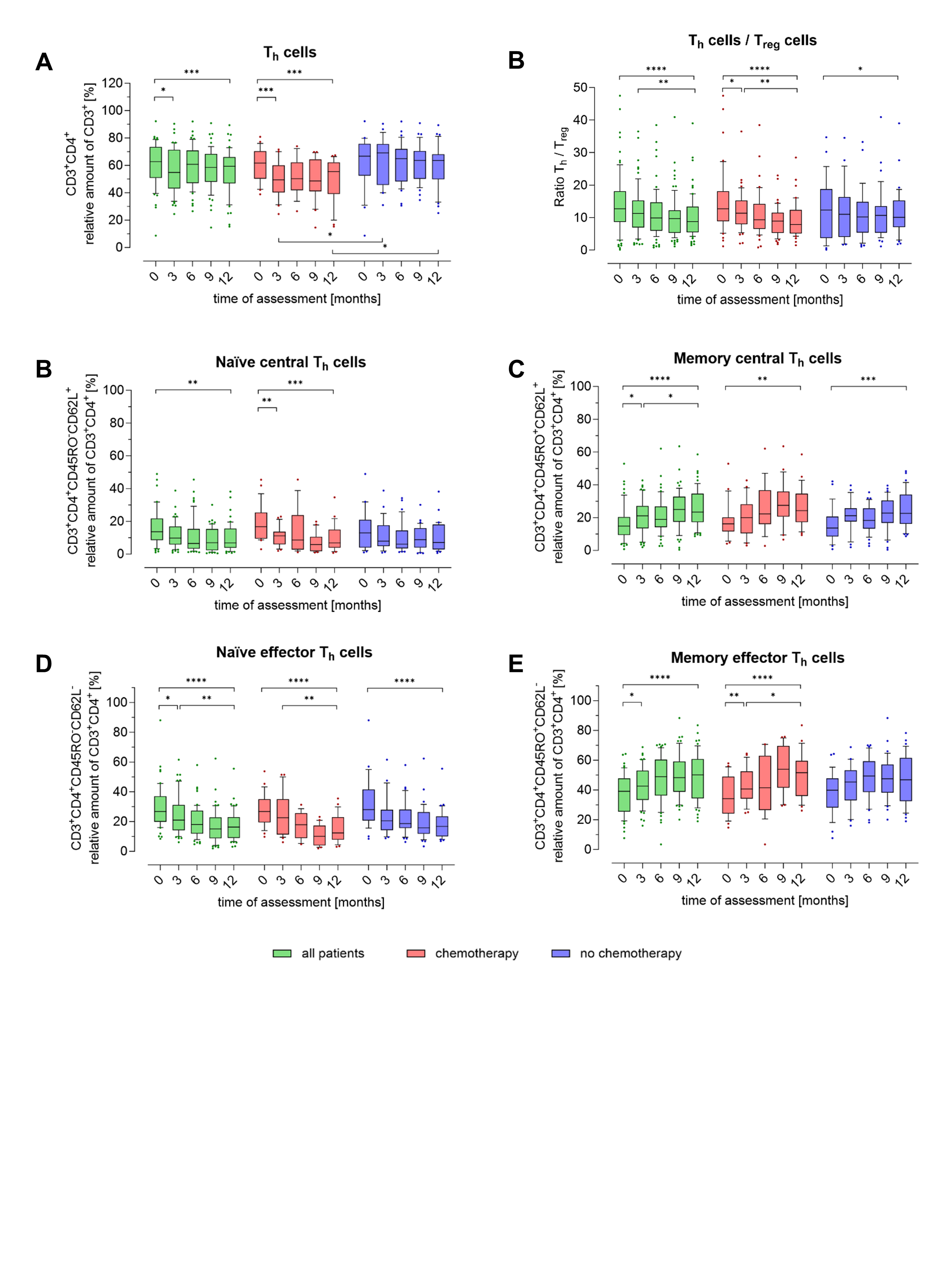

Supplement: Supplementary file 14 — Supplementary Material 14: Figure S7: Peripheral blood subpopulations of T helper cells in breast cancer patients receiving chemotherapy without potentially immunomodulatory therapy during one year. [file 13058_2025_1997_MOESM14_ESM.tif]

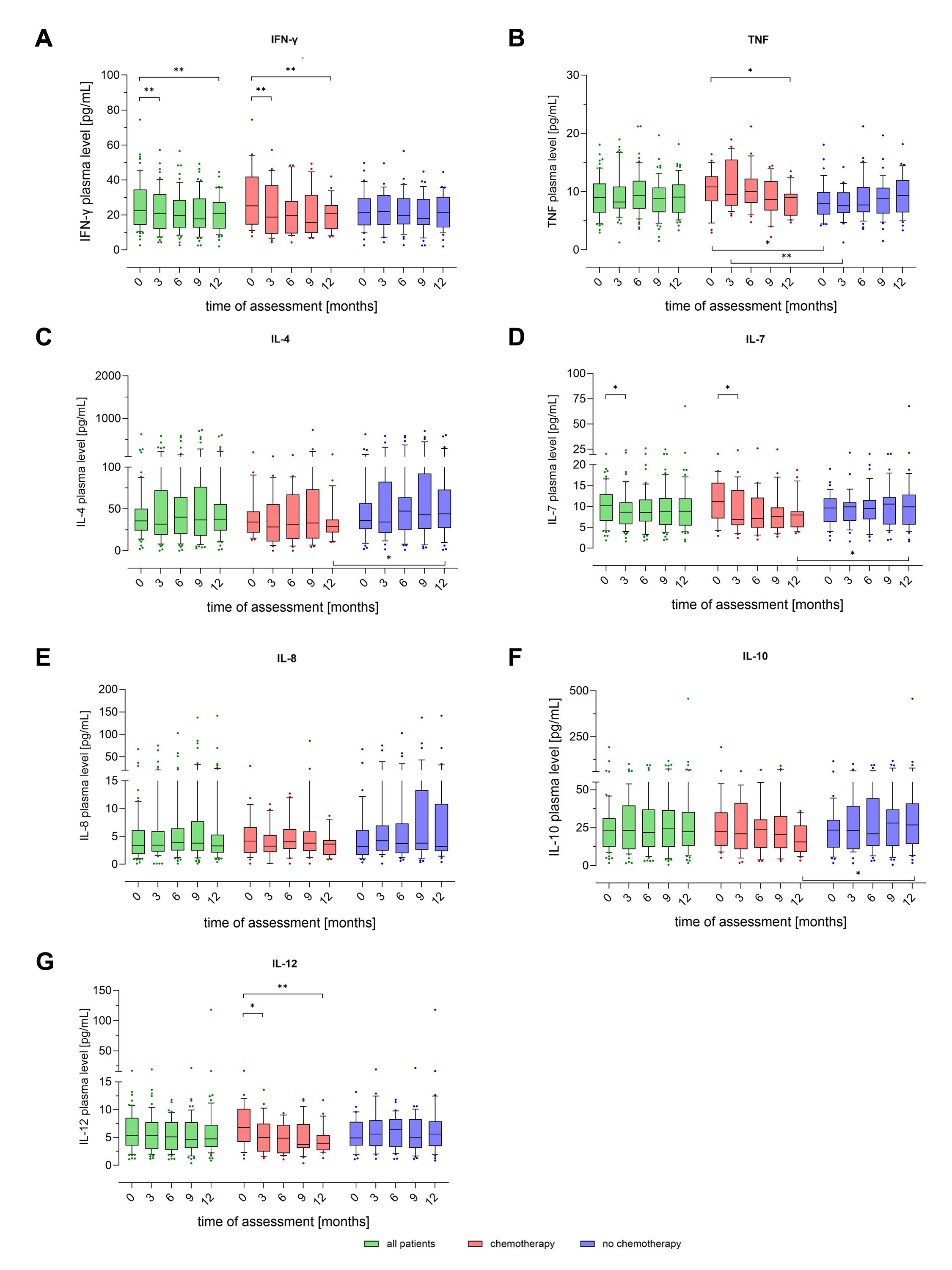

Supplement: Supplementary file 15 — Supplementary Material 15: Figure S8: Peripheral blood subpopulations of Th1 and Th2 cells in breast cancer patients receiving chemotherapy without potentially immunomodulatory therapy during one year. [file 13058_2025_1997_MOESM15_ESM.tif]

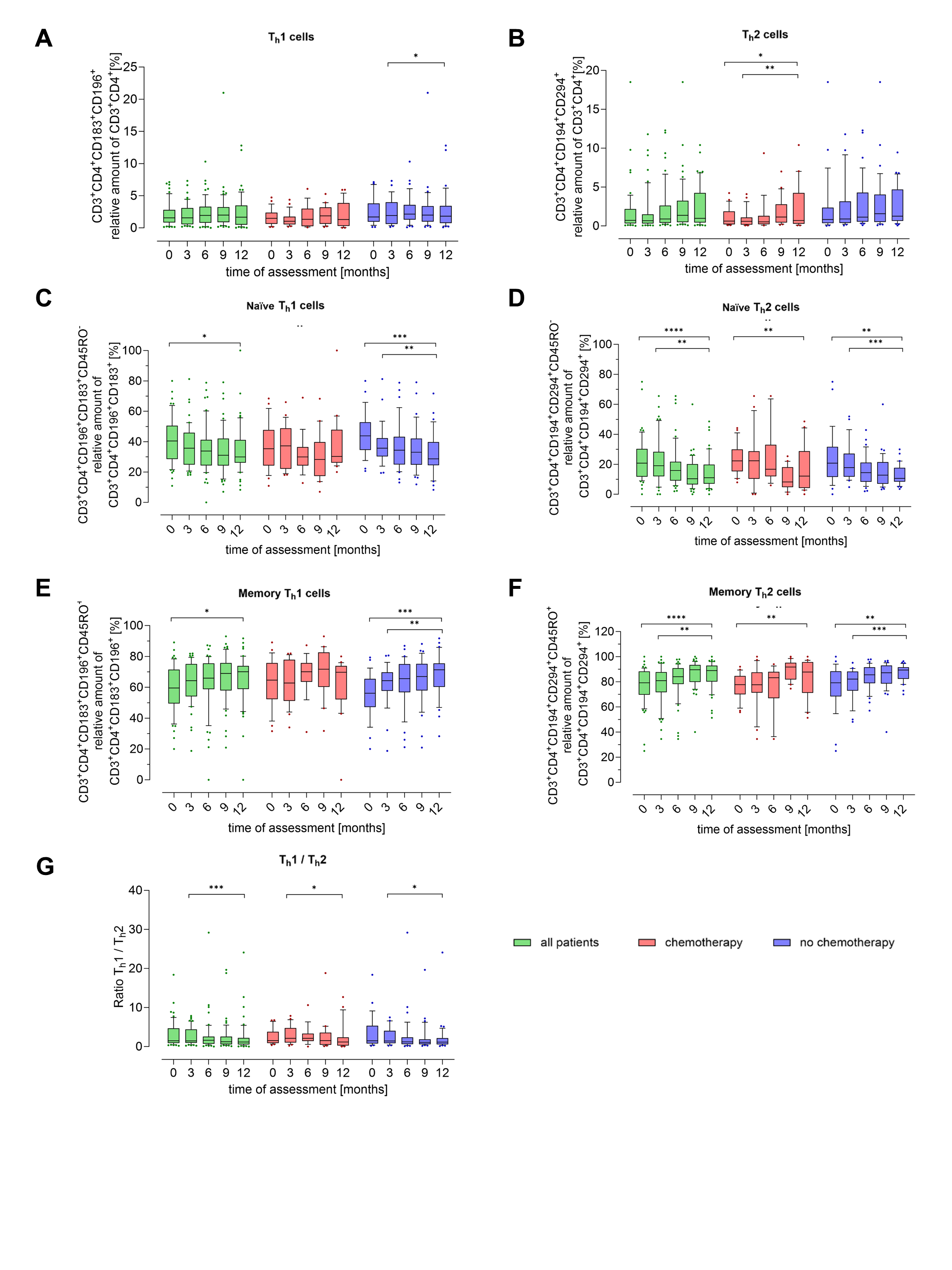

Supplement: Supplementary file 16 — Supplementary Material 16: Figure S9: Peripheral blood plasma cytokine levels in breast cancer patients receiving chemotherapy without potentially immunomodulatory therapy during one year. [file 13058_2025_1997_MOESM16_ESM.tif]
